# Synthetic scope and DFT analysis of the chiral binap–gold(I) complex-catalyzed 1,3-dipolar cycloaddition of azlactones with alkenes

**DOI:** 10.3762/bjoc.9.280

**Published:** 2013-11-11

**Authors:** María Martín-Rodríguez, Luis M Castelló, Carmen Nájera, José M Sansano, Olatz Larrañaga, Abel de Cózar, Fernando P Cossío

**Affiliations:** 1 Departamento de Química Orgánica e Instituto de Síntesis Orgánica, Universidad de Alicante, Apdo. 99, 03080-Alicante, Spain; 2 Departamento de Química Orgánica I, Facultad de Química, Universidad del País Vasco, Apdo. 1072, E-20018 San Sebastián, Spain; 3 IKERBASQUE, Basque Foundation for Science, 48011 Bilbao, Spain

**Keywords:** asymmetric catalysis, DFT, 1,3-dipolar cycloaddition, gold catalysis, NICS, NTR, oxazolones, prolines

## Abstract

The 1,3-dipolar cycloaddition between glycine-derived azlactones with maleimides is efficiently catalyzed by the dimeric chiral complex [(*S*_a_)-Binap·AuTFA]_2_. The alanine-derived oxazolone only reacts with *tert*-butyl acrylate giving anomalous regiochemistry, which is explained and supported by Natural Resonance Theory and Nucleus Independent Chemical Shifts calculations. The origin of the high enantiodiscrimination observed with maleimides and *tert*-butyl acrylate is analyzed using DFT computed at M06/Lanl2dz//ONIOM(b3lyp/Lanl2dz:UFF) level. Several applications of these cycloadducts in the synthesis of new proline derivatives with a 2,5-*trans*-arrangement and in the preparation of complex fused polycyclic molecules are described.

## Introduction

**Figure 1:**
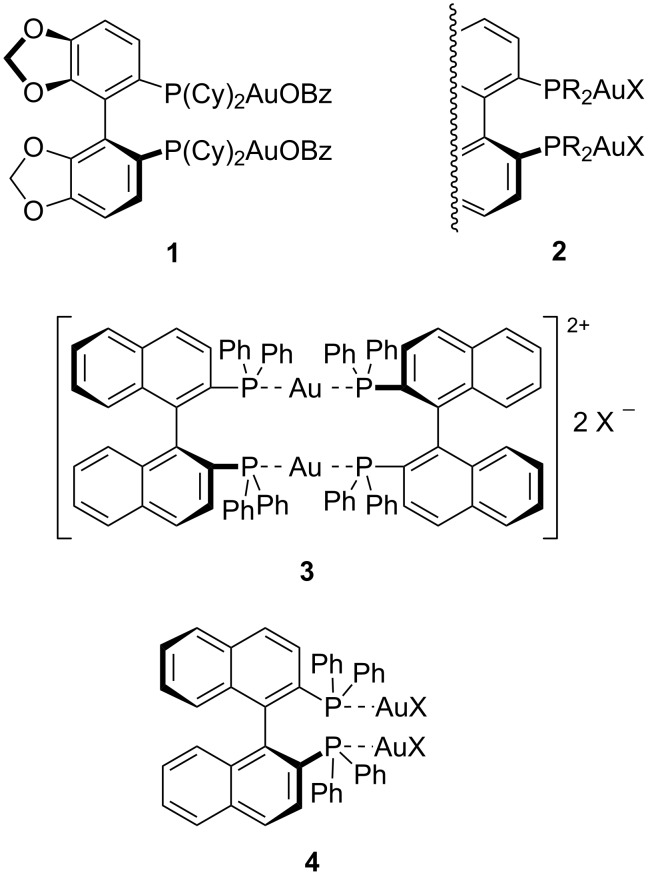
Chiral gold(I) complexes employed in 1,3-DC involving azomethine ylides.

The synthesis of α-amino acids employing an α-amino carbonyl template constitutes the most straightforward route to introduce the α-side chain [1]. As a valid example, oxazol-5-(4*H*)-ones (azlactones) are suitable heterocycles to perform this C–C bond generation based strategy affording both quaternized and non quaternized α-amino acid derivatives [2–5]. The preparation of azlactones is very simple and their reactivity is very diverse due to their functional groups [2–5]. Many enantioselective and/or diastereoselective processes have been focussed on the elaboration of enantiomerically enriched new non-proteinogenic α-amino acids, such as Michael-type additions [6–7], transition metal-catalyzed allylations [8], Mannich-type additions [9], aldol-type reactions [10], and for other different purposes [11–17]. These substrates can be easily transformed in münchnones, which are potential 1,3-dipoles, after deprotonation and imine-activation with a chiral Lewis acid. Despite of the easy access to this mesoionic heterocycles their enantioselective cycloadditions with electrophilic alkenes have not been exploited. Toste´s group published an efficient 1,3-dipolar cycloaddition (1,3-DC) between alanine, phenylalanine and allylglycine derived azlactones with maleimides and acrylates employing dimetallic (*S*)-Cy-Segphos(AuOBz)_2_ complex **1** as a catalyst (2 mol %) in the absence of base (Figure 1) [18–19]. This catalytic system was very effective but the reactions performed with (*R*)-Binap(AuOBz)_2_ (Figure 1) as catalyst offered a very low enantioselection, for example, a 8% ee was achieved in the 1,3-DC of alanine derived azlactone and *N*-phenylmaleimide (NPM).

Numerous gold-catalyzed transformations employing mild reaction conditions appeared during the last twelve years [20–22]. Initially, coordination arrangements of chiral gold complexes avoided high enantiodiscriminations but, recently, it has been demonstrated that chiral bis-gold complexes type **2** (Figure 1) are very efficient in asymmetric catalysis [23–24]. The high amount of gold per mole of catalyst and the chiral ligand itself make these processes somehow expensive.

The relative lower cost of chiral privileged ligand Binap (versus Cy-Segphos) and the good results obtained in the 1,3-DC of α-imino esters and electrophilic alkenes using the bis-gold(I) complex **3** (where the gold atom:ligand ratio is 1:1, Figure 1) [25–27] inspired us to test it in this azlactone involved cycloaddition. Previous experience in the 1,3-DC between imino esters and electrophilic alkenes revealed that the dimeric chiral gold complex **3** resulted to be unique efficient catalyst in terms of enantioselection rather than the bis-gold complex **4** [25–27]. This data is in a clear contrast to the previously mentioned result for the reactivity of azlactones [18–19]. In this work we describe a more extended study than the analogous one described in a preliminary communication [28] concerning the catalytic activity of complexes **3** and **4** in the 1,3-DC of oxazolones with electrophilic alkenes. Here, a deep DFT analysis and the application of other computational experiments (NRT, NICS) were compared to the experimentally observed results in order to clarify the enantio- and anomalous regioselectivity.

## Results and Discussion

Initially, the synthesis of oxazolones **5** was accomplished under mild reaction conditions by mixing *N*-acyl-α-amino acid derivatives in the presence of dehydrating agents such as carbodiimides [2–5]. Gold(I) complexes **3** and **4**, identified and characterized by Puddephatt’s group [X = trifluoroacetate (TFA)] [29–31], were obtained from NaAuCl_4_ and dimethyl sulfide and the corresponding amount of the chiral Binap ligand. Finally, the anion interchange was promoted by the addition of an equivalent amount of silver(I) salt. These complexes were used immediately after filtration through a celite path. Particularly, complexes **3** and **4** (X = TFA) could be isolated in 96 and 89% yield, respectively, but other gold(I) complexes (see Table 1) with different anions were generated in situ and used as catalysts in the same solution.

**Scheme 1:**
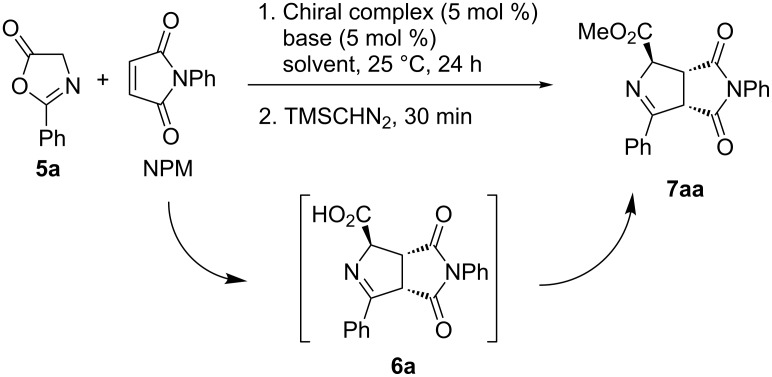
1,3-DC of azlactone **5a** and NPM.

Oxazolone derived from glycine **5a** was allowed to react with *N-*phenylmaleimide (NPM) at room temperature (25 °C approx.) using 5 mol % of the chiral catalytic complex and 5 mol % of base (Scheme 1). After completion, a large excess of trimethylsilyldiazomethane was added to obtain the methyl ester of intermediate carboxylic acid **6a** (30 min). Compound **7aa** was obtained diastereoselectively (>98:2, by ^1^H NMR spectroscopy) after purification and its absolute configuration was established according to the retention times of signals observed after HPLC analysis employing chiral columns and by comparison with the previously reported data [18–19].

Using this model reaction (Scheme 1), we tested the dimeric gold complex [(*S*_a_)-Binap·AuTFA]_2_ according to the previous experience obtained in the 1,3-DC involving imino esters and electrophilic alkenes and the reaction conditions employed by Toste’s group [18–19]. The use of fluorobenzene as solvent or co-solvent did not afford neither good conversions nor enantioselectivities, even working with the dimetallic complex **4** (X = TFA) (Table 1, entries 1–4). After the evaluation of the influence of the solvent, we concluded that toluene was the most appropriate solvent for these reactions (Table 1, entries 5–9), being the chemical yield high (90%) and the enantiodiscrimination excellent (99% ee). The presence of triethylamine as base is crucial for this transformation, it ensures both of the high conversions and enantioselections (Table 1, entries 11–14). Other different bases such as DBU, and DIPEA did not improve the result achieved by the analogous reaction carried out with triethylamine (Table 1, entries 12 and 13). Again, the presence of the chiral catalytic complex **4** (X = TFA) did not give the expected results (Table 1, entries 6 and 10). The enantiomerically pure form of **7aa** with opposite absolute configuration was isolated by working in the presence of [(*R*_a_)-Binap·AuTFA]_2_ complex (Table 1, entry 11). Surprisingly, no reaction was observed in the presence of silver(I) complex (*S*_a_)-Binap·AgTFA (Table 1, entry 15). In this section the effect of different anions of the metal complex was studied as well. In contrast with the negligible reaction observed when poor basic anion, such as perchlorate, was essayed (Table 1, entry 16), anions with basic character such as acetate or benzoate, incorporated to the chemical structure of the gold(I) catalyst, promoted the enantioselective reaction although with lower efficiency (Table 1, entries 17 and 18) [32].

**Table 1:** Optimization of the 1,3-dipolar cycloaddition of **5a** and NPM using chiral complexes.

Entry	Catalyst/X^a^	Solvent	Base	Yield^b^ (%)	ee^c^ (%)

1	(*S*_a_)-**3**/TFA	PhF	Et_3_N	<50	7
2	(*S*_a_)-**4**/TFA	PhF	Et_3_N	^___d^	<5
3	(*S*_a_)-**3**/TFA	PhF-THF	Et_3_N	^___d^	^___d^
4	(*S*_a_)-**4**/TFA	PhF-THF	Et_3_N	^___d^	<5
5	(*S*_a_)-**3**/TFA	THF	Et_3_N	76	49
6	(*S*_a_)-**4**/TFA	THF	Et_3_N	^___d^	nd
7	(*S*_a_)-**3**/TFA	DCM	Et_3_N	88	80
8	(*S*_a_)-**3**/TFA	Et_2_O	Et_3_N	85	76
9	(*S*_a_)-**3**/TFA	PhMe	Et_3_N	90	99
10	(*S*_a_)-**4**/TFA	PhMe	Et_3_N	^___d^	^___d^
11	(*R*_a_)-**3**/TFA	PhMe	Et_3_N	90	–99
12	(*S*_a_)-**3**/TFA	PhMe	DBU	70^e^	80
13	(*S*_a_)-**3**/TFA	PhMe	DIPEA	90	98
14	(*S*_a_)-**3**/TFA	PhMe	none	^___d^	^___d^
15	(*S*_a_)-Binap·AgTFA	PhMe	Et_3_N	^___d^	^___d^
16	(*S*_a_)-**3**/ClO_4_	PhMe	Et_3_N	^___d^	^___d^
17	(*S*_a_)-**3**/OAc	PhMe	Et_3_N	90	64
18	(*S*_a_)-**3**/OBz	PhMe	Et_3_N	91	74

^a^The gold catalysts were freshly generated in situ. ^b^After flash chromatography (silica gel). The observed *exo:endo* ratio was always >98:2 (^1^H NMR). ^c^Determined by using analytical chiral HPLC columns (Daicel, Chiralpak AS). ^d^Not determined.

**Scheme 2:**
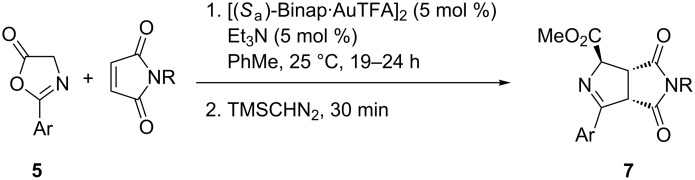
General 1,3-DC between azlactones **5** with maleimides.

The scope of the reaction was next surveyed. Firstly, azlactone **5a** was allowed to react with several maleimides (Scheme 2, and Table 2, entries 1–10). NPM and 4-acetoxyphenylmaleimide were the best entries of this series affording almost enantiomerically pure bicyclic products **7aa** and **7ae**, respectively (Table 2, entries 1 and 8). *N-*Substituted methyl, ethyl and benzylmaleimides did not afford compounds **7** with so high enantioselections. Then, a lower temperature (−20 °C) was attempted but the increment of ee for *N*-methyl- and *N-*ethylmaleimides was not very noticeable (Table 2, entries 2, 3 and 4, 5, respectively). Nevertheless, a gap of 21 units of ee was achieved in the case of the reaction involving *N*-benzylmaleimide (Table 2, compare entries 6 and 7). In the case of *N-*(4-bromophenyl)maleimide a good enantioselection was observed when the reaction was run at −20 °C furnishing enantiomerically pure **7af** in good chemical yields (Table 2, entries 9 and 10). The variation of the arene substituent of the azlactones promoted also excellent to good enantioselections in compounds **7ba** and **7ca** (Table 2, entries 11 and 12). Even working with an heteroaromatic substituent, such as 2-thienyl, compound **7da** was isolated in 95% ee (Table 2, entry 13).

**Table 2:** 1,3-Dipolar cycloaddition of azlactones **5a** with maleimides.

Entry	Ar, **5**^a^	R	Product **7**	Yield^b^ (%)	ee^c^ (%)

1	Ph, **5a**	Ph	**7aa**	90	99
2	Ph, **5a**	Me	**7ab**	90	54
3	Ph, **5a**	Me^d^	**7ab**	79	60
4	Ph, **5a**	Et	**7ac**	87	62
5	Ph, **5a**	Et^d^	**7ac**	70	70
6	Ph, **5a**	Bn	**7ad**	90	50
7	Ph, **5a**	Bn^d^	**7ad**	83	71
8	Ph, **5a**	4-(AcO)C_6_H_4_	**7ae**	90	99
9	Ph, **5a**	4-BrC_6_H_4_	**7af**	82	91
10	Ph, **5a**	4-BrC_6_H_4_^d^	**7af**	84	99
11	4-MeC_6_H_4_, **5b**	Ph	**7ba**	78	99
12	4-ClC_6_H_4_, **5c**	Ph	**7ca**	83	98
13	2-Thienyl, **5d**	Ph	**7da**	80	95

^a^The gold catalyst was freshly generated in situ. ^b^After flash chromatography (silica gel). The observed *exo:endo* ratio was always >98:2 (^1^H NMR). ^c^Determined by using analytical chiral HPLC columns (Daicel, Chiralpak AS). ^d^Reaction run at −20 °C.

**Scheme 3:**
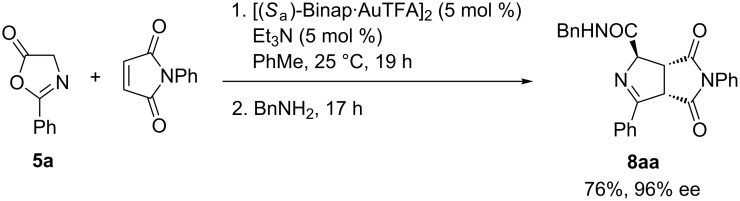
Formation of the amide **8aa**.

When benzylamine was employed as alternative quenching reagent to trimethylsilyldiazomethane, the generation of the corresponding *N*-benzylamide in 76% yield and 96% ee was achieved after 17 h at 25 °C (Scheme 3) [18–19].

**Figure 2:**
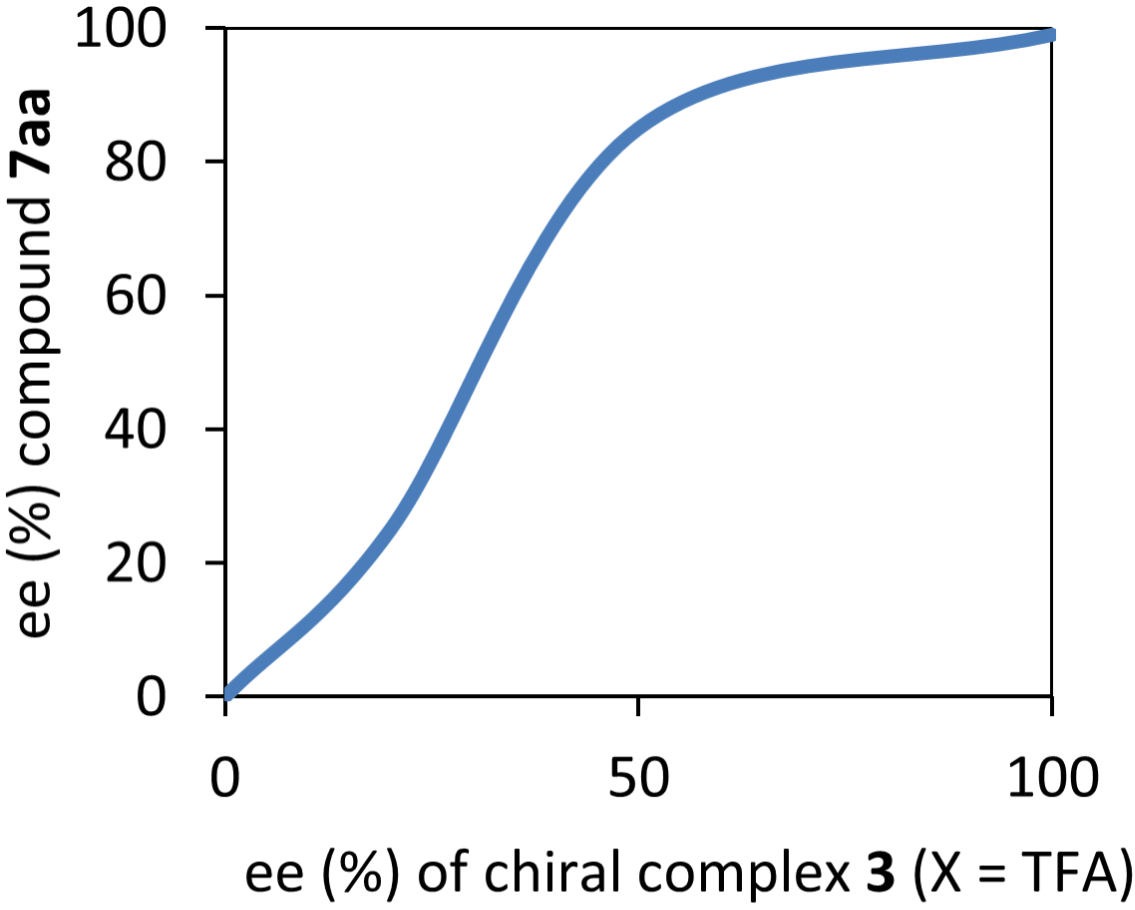
Positive non-linear effects (NLE) observed in 1,3-DC of azlactone **7aa** and NPM.

The study of the key points of the enantiodiscrimination step and mechanism for the 1,3-DC of azlactone **7aa** and NPM can be originated by the presence of a more active homochiral dimer catalyst (*S*_a_,*S*_a_)-**3** (X = TFA) with a lower TS energy with all the reaction components, rather than the corresponding heterochiral ones and even lower than homochiral dimer catalyst (*R*_a_,*R*_a_)-**3** (X = TFA). The clear positive non-linear effects (NLE) described in Figure 2 supported this hypothesis [33].

**Figure 3:**
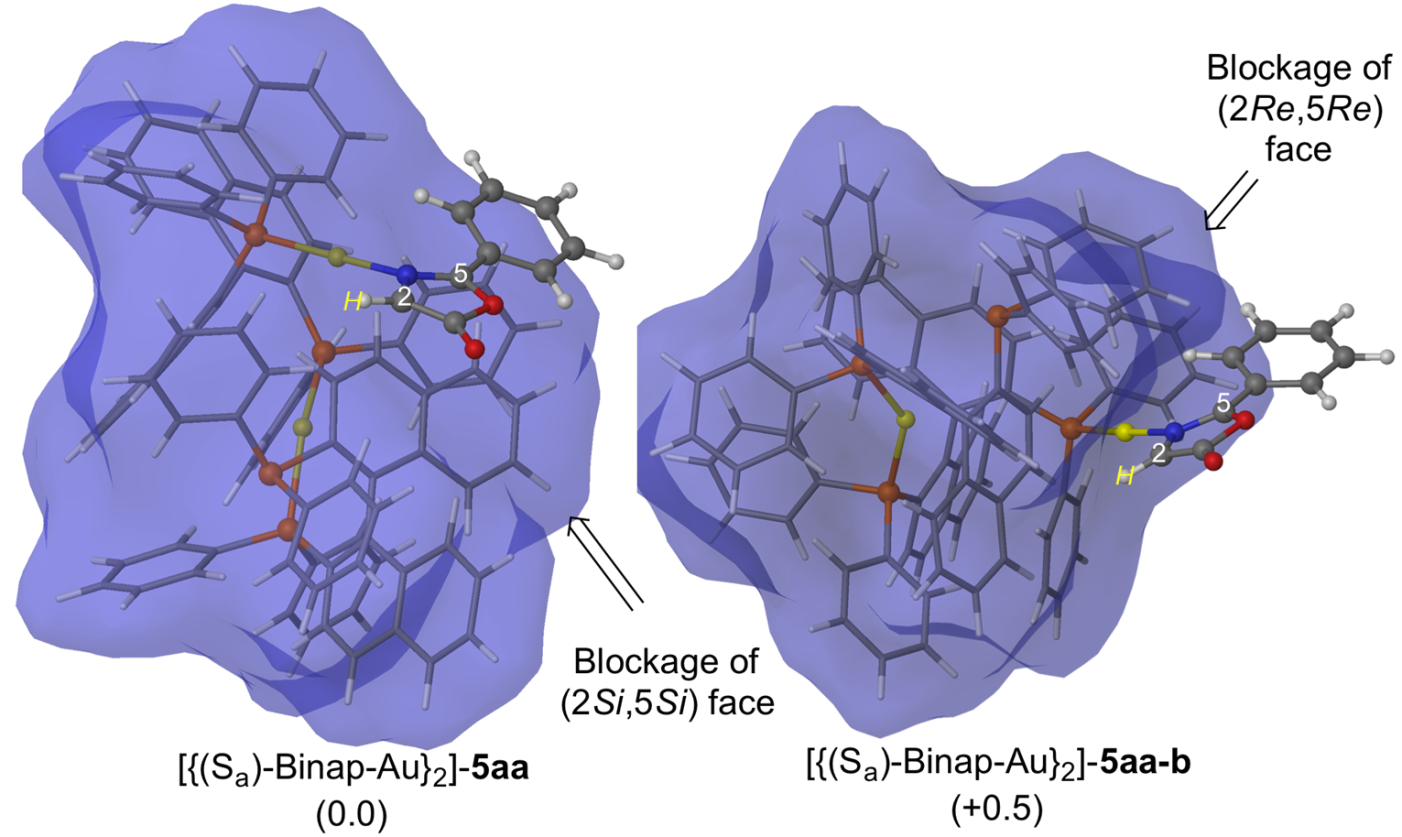
Main geometrical features and relative Gibbs free energies (in kcal mol^−1^ at 298 K) of complexes [(*S*_a_)-Binap-Au]_2_-**5aa** and [(*S*_a_)-Binap-Au]_2_-**5aa-b** computed at M06/Lanl2dz//ONIOM (b3lyp/Lanl2dz:UFF). High-level and low-level layers are represented as ball and stick and wireframe models, respectively. Blue surface represents the solvent-accessible surface with a probe radius of 1.9 Å.

Next, we studied the reaction between the oxazolone **5aa** and NPM catalyzed by [(*S*_a_)-Binap-AuTFA]_2_. In previous works, we have demonstrated that the stereoselectivity of the 1,3-DC employing chiral metallic Lewis acids arises from the blockage of one of the prochiral faces [34]. Starting from this selected conformation of the catalyst, our results show that the (2*Re*,5*Re*) prochiral face is less hindered than the other prochiral face in the most stable conformation of [{(*S*_a_)-Binap-Au}_2_]-**5aa** complex (Figure 3). As expected, the existence of dimeric gold units is crucial in the blockage of one of the prochiral faces, and therefore, in the stereochemical outcome of the final cycloadducts [26–27].

**Figure 4:**
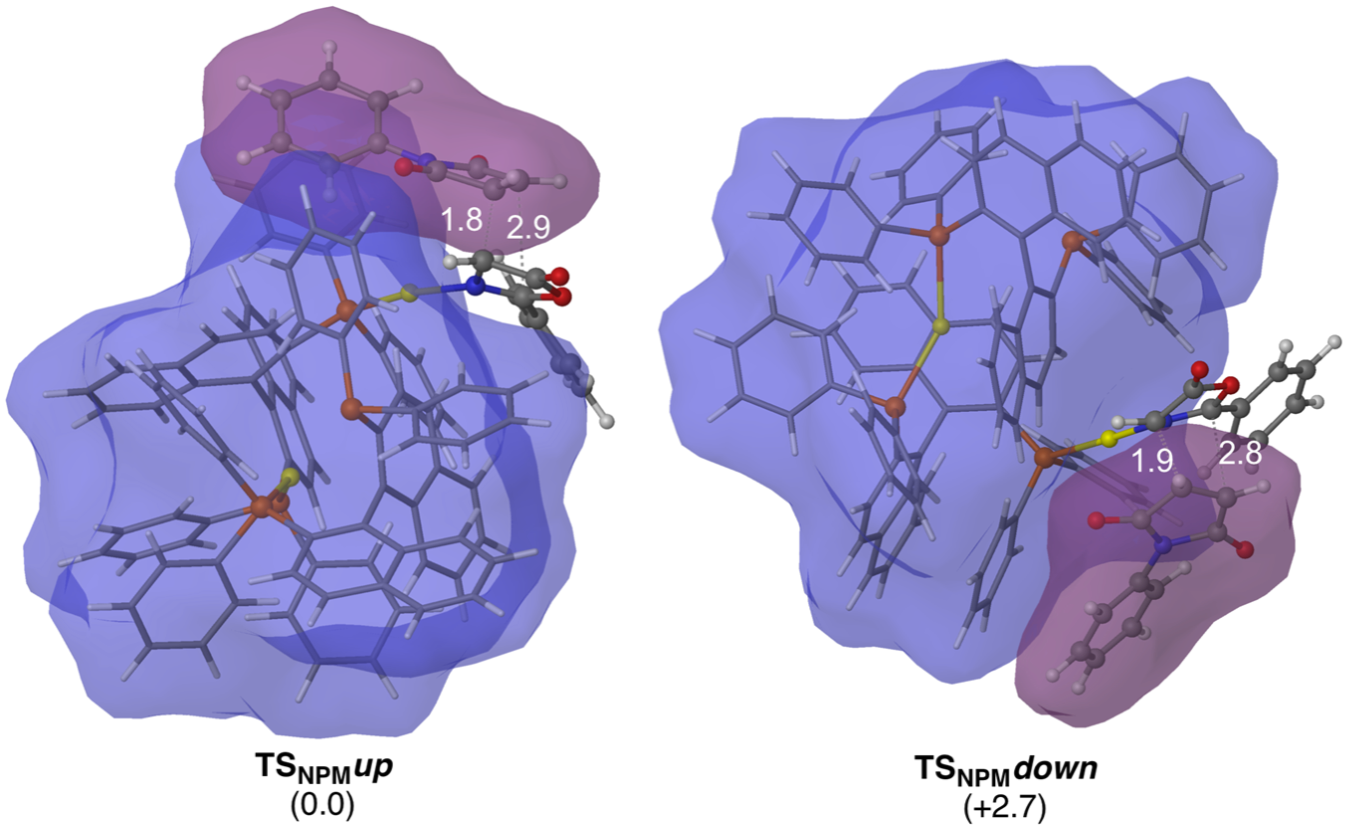
Main geometrical features and relative Gibbs free energies (in kcal mol^−1^) of the less energetic transition states associated with the 1,3-DC of **5aa** and NPM catalyzed by (*S*_a_)-Binap gold dimers computed at M06/Lanl2dz//ONIOM(b3lyp/Lanl2dz:UFF) level of theory. High-level and low level layers are represented as ball and stick and wireframe models, respectively. Distances are in Å. Blue and purple surfaces represent the solvent-accessible surface of the catalyst and NPM with a probe radius of 1.9 Å.

Refined computational results showed the *exo-*approach [35] is the preferred one. In this analysis, only that approach was considered. The less energetic computed TS are depicted on Figure 4 (see Supporting Information File 1 for further information of additional TS’s).

The computed transition structures correspond to concerted but highly asynchronous cycloadditions (Figure 4). Our calculations show that there is a different overlap between the accessible-solvent surface of the catalyst and the one of the incoming dipolarophile. That implies an increase of the 4e^−^ Pauli repulsion between the reactives in **TS_NPM_*down*** compared to **TS_NPM_*up***, and thus an increase of the activation barrier. Moreover, lower energy to deform the initial ylide (strain energy) is required in the latter TS. With that energetic diference, the computed ee is about 99%, in good agreement with the experimental results (Table 2, entry 1).

**Scheme 4:**
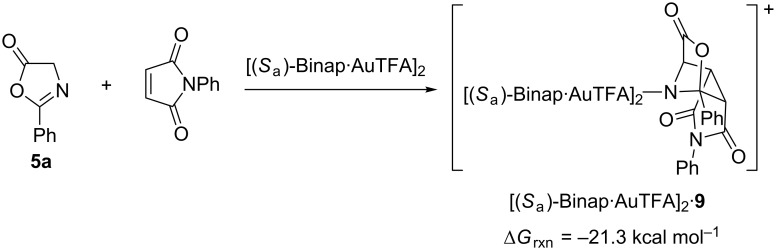
Reaction Gibbs free energy associated with the 1,3-DC of **5aa** and NPM catalyzed by (*S*_a_)-Binap gold dimers computed at M06/Lanl2dz//ONIOM (b3lyp/Lanl2dz:UFF) level of theory.

The complete reaction path of the cycloaddition process is shown in Scheme 4. We do not study computationaly the second synthetic step, namely the ring-opening of the tricyclic-cycloadduct, because that step has no relevance in the stereochemical outcome of the reaction.

**Scheme 5:**
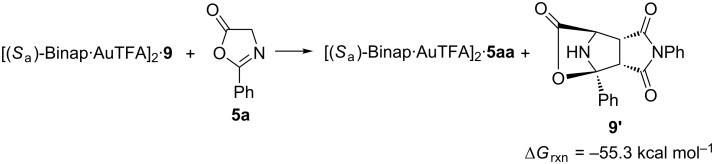
Δ*G* calculation for the recovery of the catalytic active species.

We also studied the last step of the catalytic cycle that ensures the recovery of the catalyst obtaining a favourable Gibbs energy of −55.3 kcal mol^−1^ (Scheme 5).

**Scheme 6:**
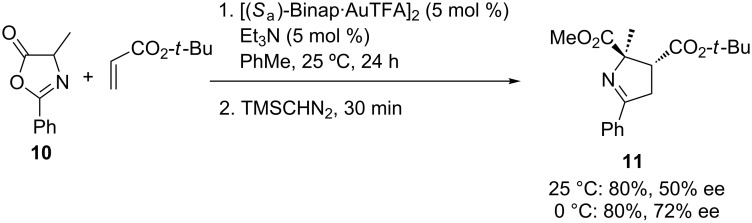
1,3-DC of azlactone **10** and *tert-*butyl acrylate.

No chemical reaction occurred when **5a** was combined with other dipolarophiles such as fumarates, maleates, vinyl phenyl sulfone, *trans*-1,2-bis(phenylsulfonyl)ethylene, chalcone, crotonaldehyde and cinnamaldehyde at the same reaction conditions [36]. Another drawback was the poor reactivity observed when α-substituted azlactones were used as starting material in the named reaction with NPM. However, the alanine-derived 4-methyloxazole-5-one **10**, surprisingly, reacted at 25 and at 0 °C with *tert*-butyl acrylate yielding cycloadduct **11** in good yields and moderate to good enantioselections (Scheme 6).

If we compare this result with previous ones obtained using α-imino esters, this last diastereoselective cycloaddition exhibited an opposite regioselection. Besides, the resulting relative configuration of Δ^1^-pyrroline **11** is equivalent to the *exo*-approach of the dipolarophile when an *endo*-transition state was the most favourable in the gold(I)-catalyzed 1,3-DC with α-imino esters and alkenes [37].

**Figure 5:**
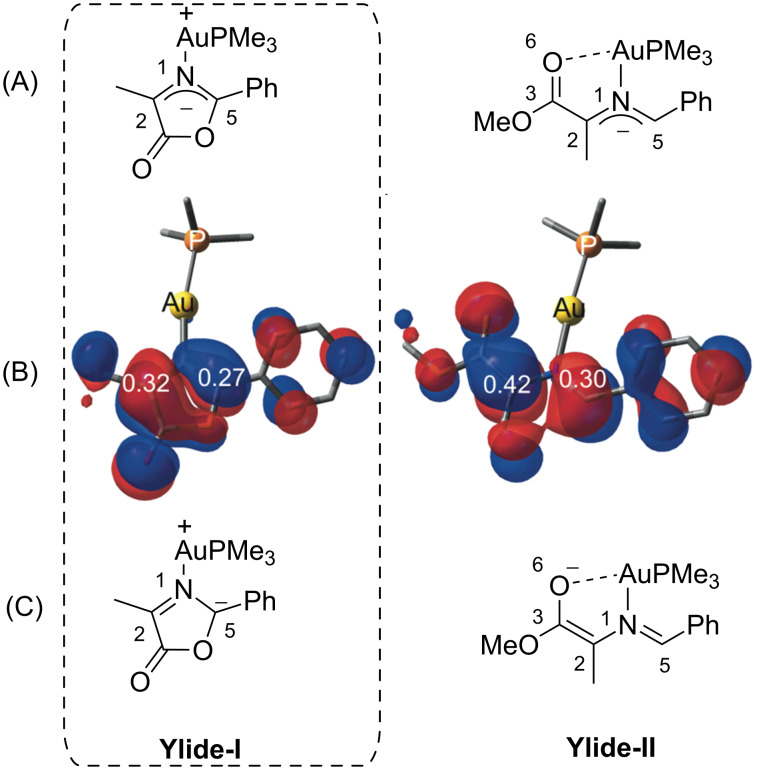
(A) Schematic representation of the model gold(I) ylides. (B) HOMO of the ylides and expansion orbital coefficient values of carbon atoms 2 and 5 computed at HF/Lanl2dz level of theory. Hydrogen atoms are omitted for clarity. (C) Most stable Lewis structures of the ylides obtained with the Natural Resonance Theory (NRT) analysis.

To gain more insight into the unexpected regioselectivity of the 1,3-DC depicted in Scheme 6, calculations within the DFT framework were performed. In the accepted mechanism of the metal catalyzed 1,3-DC of azomethine ylides and acrylates, the α-carbon atom of the azomethine ylide (C2 in Figure 5) reacts with the β-carbon of the acrylate moiety, independently of the mechanism (concerted fashion or via Michael-like transition state followed by a Mannich-like ring closure in a stepwise mechanism yields the same cycloadduct) [38]. This fact is assumed to be a consequence of the unsymmetrical electron density in the 1,3-dipole moiety, being higher in the carbon in α-position to the carboxy group (C2).

Initially, a model azomethine ylide derived from oxazolone **10** was considered (Figure 5). Moreover, an acyclic w-shaped ylide analogue (**Ylide-II**) was also studied as a reference. We chose this latter 1,3-dipole because it is known that with this kind of reactive species, the reaction yields cycloadducts possessing a standard regioselectivity in 1,3-DC with acrylates [38]. Since our goal was to understand the origins of the unusual regioselectivity observed in the reaction between dipoles of type **Ylide-I** with acrylates, trimethylphosphine was coordinated directly to the gold(I) atom in our model (Figure 5).

Analysis of atomic expansion coefficients of the HOMO of **Ylide I** reveal no significant difference between the azomethine ylides reported in Figure 5. However, Natural Resonance Theory Analysis (NRT) [39–41] shows that the negative charge in the Lewis structure of **Ylide I** is mainly placed on C5. In the case of **Ylide II**, this negative charge is placed on the oxygen of the carboxy group instead. The importance of these electronic distributions was verified by Nucleus Independent Chemical Shifts (NICS) calculations in the ring point of the oxazoline [42]. The NICS value of −7.3 ppm pointed to the aromaticity of that ring in **Ylide I**. These results explain the existence of different regioselectivities for both ylides.

**Figure 6:**
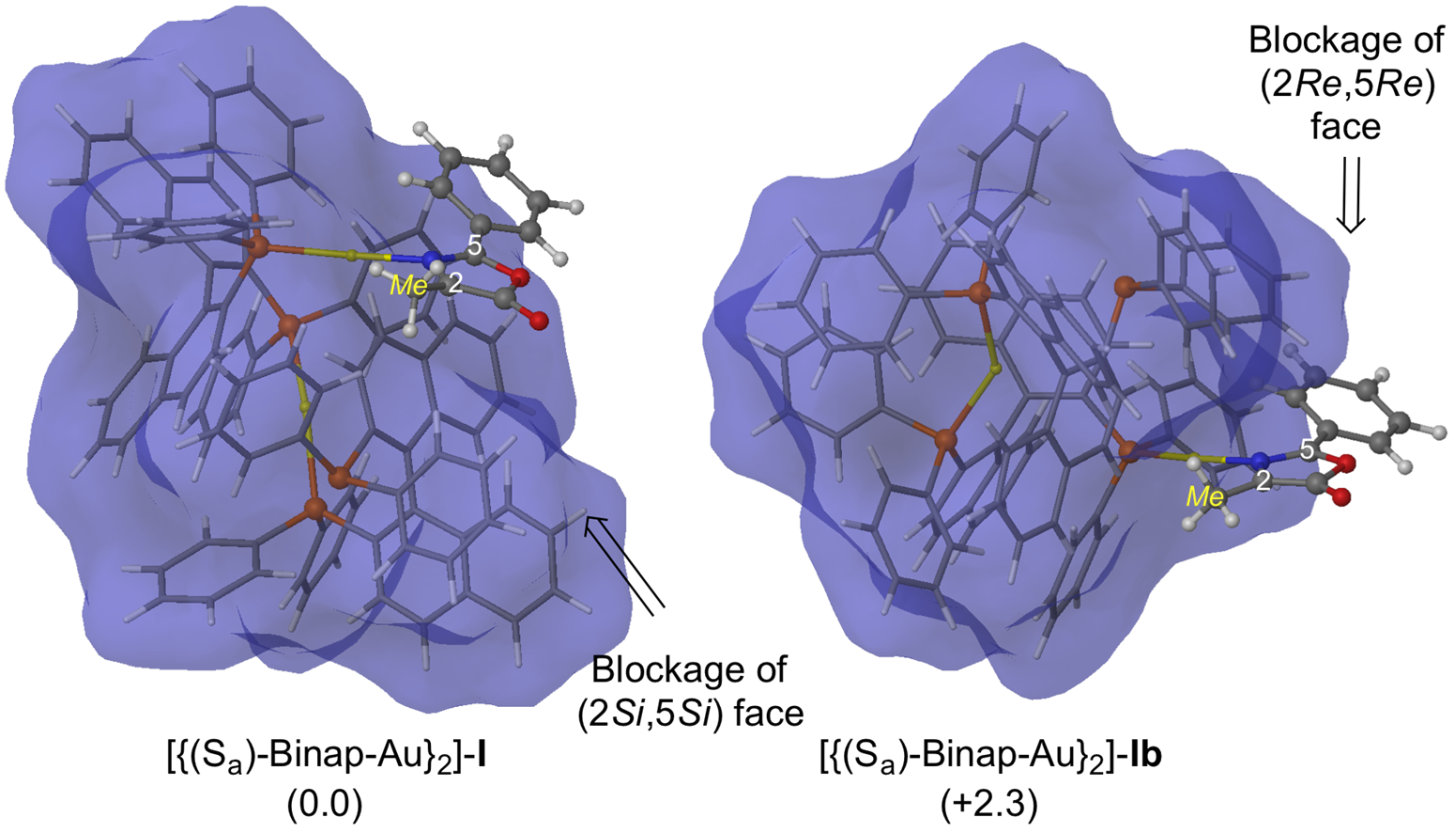
Main geometrical features and relative Gibbs free energies (in kcal mol^−1^ at 298 K) of complexes [{(*S*_a_)-Binap-Au}_2_]-**I** computed at M06/Lanl2dz//ONIOM (b3lyp/Lanl2dz:UFF). High-level and low-level layers are represented as ball and stick and wireframe models, respectively. Blue surface represents the solvent-accessible surface with a probe radius of 1.9 Å.

Following the same calculation patterns previously shown for the reaction with NPM, the results of the main geometrical features an relative Gibbs free energies were determined for the approach of the gold(I) complex·azlactone **10** to *tert-*butyl acrylate (Figure 6).

**Figure 7:**
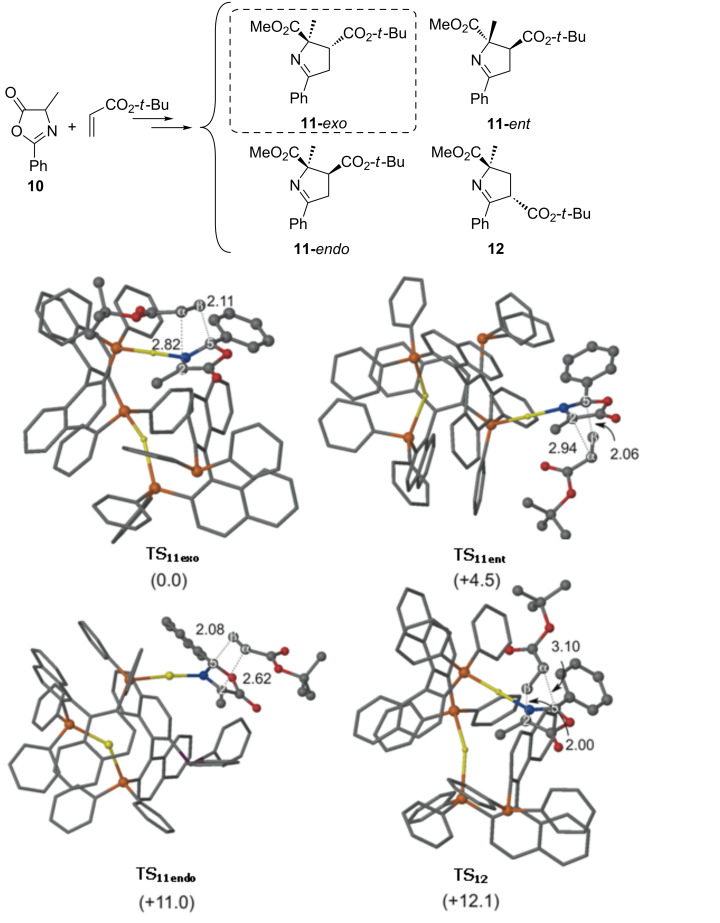
Main geometrical features and relative Gibbs free energies (in kcal mol^−1^) of the less energetic transition states associated with the 1,3-DC of **10** and *tert*-butyl acrylate catalyzed by (*S*_a_)-Binap gold dimers computed at M06/Lanl2dz//ONIOM(b3lyp/Lanl2dz:UFF). High-level and low level layers are represented as ball and stick and wireframe models, respectively. Distances are in Å. Hydrogen atoms are omitted for clarity.

In order to have a complete view of the reaction mechanism, all transition structures corresponding to the *endo-* or *exo-*approaches of the acrylate moiety as well as possible regiochemistry of the selected 1,3-DC, were considered. The main geometrical features of the less energetic transition structures are depicted in Figure 7.

Our calculations show that the less energetic transition structure associated with the 1,3-DC of **10** and *tert*-butyl acrylate is **TS_11exo_** (Figure 7), is in good agreement with the experimental results in which a high ee of the corresponding stereoisomer was observed. The formation of the enantiomer (**TS_11ent_**) was found to have an activation barrier of 4.5 kcal mol^−1^ higher in energy. That difference can be a consequence of the higher strain energy necessary to deform the initial ylide. Our calculations also pointed out the stabilizing interaction of the carboxy group of the incoming acrylate and the gold atom closest to the ylide moiety, despite the long distance (*d*_Au-C=O_ = 2.8 Å). In fact, the *exo*-approach is ca. 11 kcal mol^−1^ lower in energy than the *endo* analogue (**TS_11exo_** vs **TS_11endo_** in Figure 7). Moreover, the a priori expected regiochemistry of the cycloaduct, in which C2–Cβ and C5–Cα are new bonds (**12**), was considered. In this case, **TS_12_** is 12.1 kcal mol^−1^ higher in energy than **TS_11exo_**. It is noticeable that transition structures associated with the formation of C2–Cα and C5–Cβ bonds (**TS_11exo_**, **TS_11ent_** and **TS_11endo_**) correspond to concerted but highly asynchronous cycloadditions. On the other hand, **TS_12_** is associated with a stepwise mechanism.

**Scheme 7:**
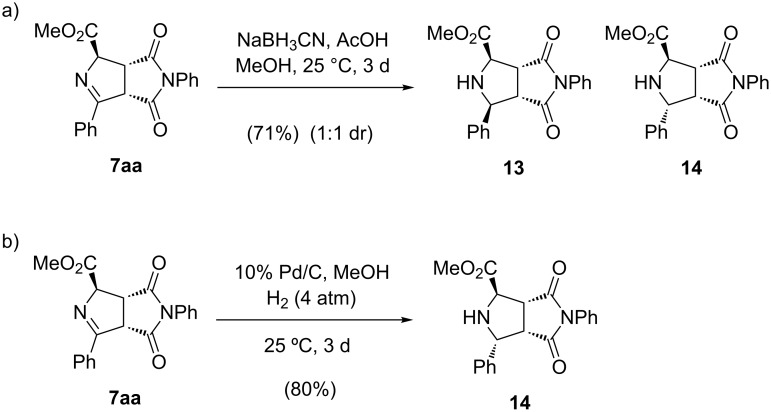
Reduction of heterocycle **7aa** under different conditions.

As possible applications of the resulting pyrrolines **7aa**, it was submitted to different transformations. For example, it could be reduced to the corresponding pyrrolidines employing sodium cyanoborohydride in acidic media. In this reaction, a 1:1 mixture of 2,5-*cis-*pyrrolidine **13** and its 5-epimer **14** (2,5-*trans*) was isolated in good chemical yield (71%) (Scheme 7, reaction a). Fortunately, 5-epimer **14** (2,5-*trans*) was diastereoselectively generated through a 10% Pd/C-catalyzed hydrogenation using 4 atmospheres of hydrogen during three days at 25 °C (Scheme 7, reaction b). This *trans-* arrangement in molecule **14** is not very easy to built because several steps were needed using other synthetic strategies [43].

**Scheme 8:**
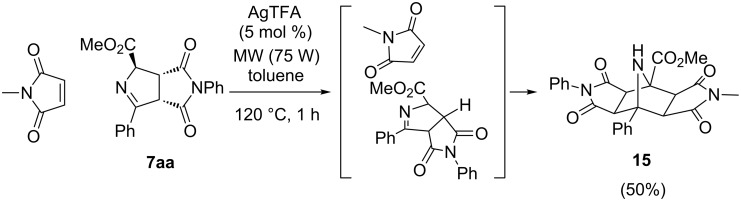
Double 1,3-DC to give polycycle **15**.

Pyrrolines also possess a typical 1,3-dipole precursor structure (azomethine ylide), so a second cycloaddition was attempted with a new equivalent of *N-*methylmaleimide. The reaction took place under microwave assisted heating (1 h, 75 W) using triethylamine as base and toluene as solvent at 120 °C. Polycyclic compound **15** was finally obtained in 50% yield as single diastereoisomer (Scheme 8). Despite being a solid product it was not possible to perform an X-ray diffraction analysis. Positive (CH derived from NPM with the CH derived from NMM) nOe experiments supported the drawn absolute configuration of **15**.

**Scheme 9:**
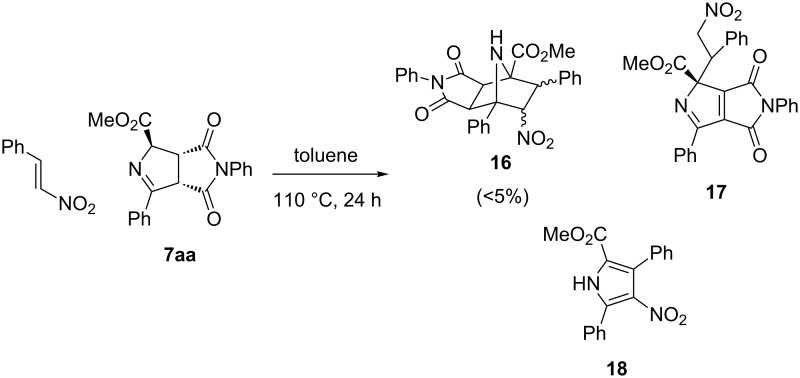
Reaction between **7aa** and nitrostyrene.

Other different dipolarophiles were attempted to react with starting **7aa** obtaining very complex mixtures including decomposed materials. In the most cases, reactions had to be refluxed for 24 h (110 °C, toluene) because microwave assisted irradiation was not as effective as occurred in the reaction with NMM. For example, the purification of the crude reaction mixture of the cycloaddition of **7aa** with β-nitrostyrene afforded an overall poor yield (~28%) of a complex 4:15:10 mixture of three compounds (**16**, **17**, and **18**) (Scheme 9) [44]. The desired compound **16** was identified (almost as unique diastereoisomer) in low chemical yield (<5%) together with two pyrrole derivatives **17** (only one stereoisomer), and **18**. The last compound was formed by a retro-cycloaddition of the pyrroline **7aa** with elimination of NMM, which was favoured by a prolonged heating [45].

## Conclusion

In this work it has been demonstrated the efficiency of the chiral [BinapAuTFA]_2_ complexes in the enantioselective 1,3-DC between azlactone derived from glycine and maleimides, especially those containing a *N-*aromatic substituent, and between alanine derived oxazolone with *tert-*butyl acrylate. In the last example the regiochemistry was totally opposite to the common trend of these cycloadditions. This behaviour has been explained for the first time using NRT, NICS, whilst DFT calculations served to justify the elevated enantioselection observed in the 1,3-DC between azlactones and maleimides. The general scope is not very wide but enantioselections obtained are quite good. Very interesting pyrrolidines with a *trans*-arrangement were obtained after hydrogenation of the pyrroline precursor.

## Supporting Information

Description of all procedures and characterization of all new compounds, as well as computational details and coordinate tables are reported in the Supporting Information.

File 1 Experimental and analytical data.
