# Supplementary material for: Synthetic scope and DFT analysis of the chiral binap–gold(I) complex-catalyzed 1,3-dipolar cycloaddition of azlactones with alkenes
Source: Beilstein J Org Chem. 2013 Nov 11;9:2422–33. doi: 10.3762/bjoc.9.280 (PMC3869218; doi:10.3762/bjoc.9.280)
Supplement: File 1 — Experimental and analytical data. [file Beilstein_J_Org_Chem-09-2422-s001.pdf]

Supporting Information
for

Synthetic scope and DFT analysis of the chiral Binap–gold(I) complex-catalyzed 1,3-dipolar cycloaddition of azlactones with alkenes

María Martín-Rodríguez[1], Luis M. Castelló,[1] Carmen Nájera*,$,1, José M. Sansano*,$,1, Olatz Larrañaga,[2] Abel de Cózar[2,3], Fernando P. Cossio*,#,2

Address: [1]Departamento de Química Orgánica e Instituto de Síntesis Orgánica, Universidad de Alicante, Apdo. 99, 03080-Alicante, Spain, [2]Departamento de Química Orgánica I, Facultad de Química, Universidad del País Vasco, Apdo. 1072, E-20018 San Sebastián, Spain and [3]IKERBASQUE, Basque Foundation for Science, 48011 Bilbao, Spain.

Email: Carmen Nájera - cnajera@ua.es; José M. Sansano - jmsansano@ua.es; Fernando P. Cossío - fp.cossio@ua.es

*Corresponding author

$Corresponding author for experimental details

#Corresponding author for computational data

Experimental and analytical data

Table of contents

1. **General S3**

2. **Computational methods S3**

3. **1,3-Dipolar cycloaddition of azlactones 5 and 10 with maleimides or *tert*-butyl acrylate. General procedure S3**

4. **Cycloaddition of NMM with 7aa. Synthesis of polycyclic structure 15. .... S6**

5. **Computational data S7**

6. **Cartesian coordinates S8**

7. **References S48**

1. General.

All reactions were carried out in the absence of light. Anhydrous solvents were freshly distilled under an argon atmosphere. Aldehydes were also distilled prior to use for the elaboration of the iminoesters [1,2,3,4,5,6]. Azlactones were prepared according to the previous procedures by other groups. Melting points were determined with a Reichert Thermovar hot plate apparatus and are uncorrected. Only the structurally most important peaks of the IR spectra (recorded on a Nicolet 510 P-FT and on a Jasco FTIR 4100) are listed. ^{1}H NMR (300 MHz) and ^{13}C NMR (75 MHz) spectra were obtained on a Bruker AC-300 using $CDCl_3$ as solvent and TMS as internal standard, unless otherwise stated. Optical rotations were measured on a Perkin Elmer 341 polarimeter. HPLC analyses were performed on a JASCO 2000-series equipped with a chiral column (detailed for each compound in the main text), using mixtures of *n*-hexane/isopropyl alcohol as mobile phase, at 25 °C. Low-resolution electron impact (EI) mass spectra were obtained at 70 eV on a Shimadzu QP-5000 and high-resolution mass spectra were obtained on a Finnigan VG Platform. HRMS (EI) were recorded on a Finnigan MAT 95S. Microanalyses were performed on a Perkin Elmer 2400 and a Carlo Erba EA1108. Analytical TLC was performed on Schleicher & Schuell F1400/LS silica gel plates and the spots were visualized under UV light (λ = 254 nm). For flash chromatography we employed Merck silica gel 60 (0.040–0.063 mm). Complexes were prepared according to the reported procedure (see text). All of the transformations performed with silver catalysts were performed in the absence of light. Microwave-assisted reactions were performed in a CEM Discover Sp reactor.

2. Computational methods.

Hybrid QM/MM calculations for optimizations of saddle points were performed in terms of ONIOM [7,8,9] method implemented in GAUSSIAN09 suite of programs [10]. Ball and stick model in Figures 6 and 7 shows atoms included in the high-level layer, and a wire model is used to represent atoms included in the low-level layer. In the high-level layer, the electron correlation was partially taken into account by using the hybrid functional B3LYP[11,12,13,14,15,16] combined with Hay–Wadt small core effective potential (ECP) [17] basis set. UFF [18] molecular mechanics force field was employed in the low-level layer. Thermal corrections of Gibbs free energies were computed at the same level of theory and were not scaled. Single point energy calculations of the optimized geometries were computed with M06 functional, because it provides reliable calculations of dispersion energies, especially in highly branched systems [19,20,21]. All stationary points were characterized by harmonic analysis. Reactant intermediates and cycloadducts have positive definite Hessian matrices. Transition structures show only one negative eigenvalue in their diagonalized force constant matrices, and their associated eigenvectors were confirmed to correspond to the motion along the reaction coordinate under consideration. Natural Resonance Theory analyses (NRT) were computed by means of the NBO 5.0 program included in the Gaussian09 suite [22,23,24].

3. 1,3-Dipolar cycloaddition of azlactones 5 and 10 with maleimides or *tert*-butyl acrylate. General procedure [25]**.**

To a solution of the in situ prepared chiral gold complex[26,27,28] (0.05 mmol) in toluene (2 mL) was added, at room temperature, a solution of the azlactone (0.5

mmol) and the dipolarophile (0.55 mmol) in toluene (2 mL). Triethylamine (7μL, 0.05 mmol) was added and the mixture stirred at the corresponding temperature for 1 d (see, Tables). Once the reaction was completed trimethylsilyl diazomethane (1 mL of 2 M solution in hexanes, 2 mmol) was added at 0 °C and the mixture was stirred at 25 °C for 30 min. The reaction was filtered off through a Celite pad and the organic filtrate was directly evaporated and the residue was purified by recrystallization or by flash chromatography yielding pure pyrrolines **7** or **11**.
Alternatively, instead of trimethylsilyl diazomethane, benzylamine (214 μL, 2 mmol) was added and the mixture was stirred at 25 °C for 17 h. The reaction was filtered off through a Celite pad and the organic filtrate was directly evaporated and the residue was purified by recrystallization or by flash chromatography yielding pure compound **8aa**.

(1*R*,3a*S*,6a*R*)-Methyl 4,6-dioxo-3,5-diphenyl-1,3a,4,5,6,6a-hexahydropyrrolo[3,4-*c*]pyrrole-1-carboxylate (7aa) [5,6].

(1*R*,3a*S*,6a*R*)-Methyl 5-methyl-4,6-dioxo-3-phenyl-1,3a,4,5,6,6a-hexahydro-pyrrolo[3,4-*c*]pyrrole-1-carboxylate (7ab) [25]**:** Colourless oil; $[\alpha]_D^{20}$ = +39º (*c* 0.9, CH_2Cl_2, 60% *ee* HPLC); HPLC (Chiralpak AD-H; 1 mL/min; *n*-hexane/*i*PrOH, 80:20; λ = 220 nm) t_{Rmaj} 22 , t_{Rmin} 25 min. R_f 0.56 (*n*-hexane/ethyl acetate 1:1); IR (neat) ν_{max}: 1699,1652.7 cm^{-1}; ^{1}H RMN (400 MHz, $CDCl_3$) δ_H: 2.96 (NC*H*$_3$), 3.84 (s, 3H, CO_2Me), 4.09 (dd, *J* = 8.4, 2.8 Hz, 1H, C*H*CCHN), 4.77 (dd, *J* = 8.4, 2.8 Hz, 1H, C*H*CN), 5.26 (t, *J* = 2.8 Hz,1H, C*H*CO_2Me), 7.62-7.36 (m, 3H, Ar*H*), and 8.22–8.13 (m, 2H, Ar*H*); ^{13}C RMN ($CDCl_3$) δ_C: 25.4 (*C*H_3N), 47.4 (*C*HCHCO_2Me), 53.2 (*C*HCN), 56.5 (OCH_3), 77.2 (*C*HCO_2CH_3), 128.5, 129.7, 131.2, 132.1, (Ar*C*), 169.3 (CN), 170.4, 172.5 (2xN*C*O), and 176.3 (CO_2Me); MS (EI) *m/z* (%): 286 (M^+, 21); HRMS-ESI required for $C_{15}H_{14}N_2O_4$: 286.0954; found: 286.0953.

(1*R*,3a*S*,6a*R*)-Methyl 5-ethyl-4,6-dioxo-3-phenyl-1,3a,4,5,6,6a-hexahydro-pyrrolo[3,4-*c*]pyrrole-1-carboxylate (7ac) [25]**:** Colourless oil; $[\alpha]_D^{20}$ = +45º (*c* 1.1, CH_2Cl_2, 70% *ee* HPLC); HPLC (Chiralpak AD-H; 1 mL/min; *n*-hexane/*i*PrOH, 80:20; λ = 220 nm) t_{Rmaj} 22 , t_{Rmin} 25 min. R_f 0.56 (*n*-hexane/ethyl acetate 1:1); IR (neat) ν_{max}: 1699, 1653 cm^{-1}; ^{1}H RMN δ_H: 1.13 (t, *J* = 7.2 Hz, 3H, C*H*$_3$CH$_2$), 3.55-3.48 (m, 2H, CH$_3$C*H*$_2$), 3.84 (s, 3H, CO_2Me), 4.07 (dd, *J*= 8.4, 3.1 Hz, 1H, C*H*CONEt), 4.75 (dd, *J* = 8.4, 2.5 Hz, 1H, C*H*CN), 5.25 (t, *J* = 2.8 Hz, 1H, C*H*CO_2Me), 7.62-7.36 (m, 3H, ArC*H*), and 8.22–8.13 (m, 2H, ArC*H*); ^{13}C RMN ($CDCl_3$) δ_C: 12.9 (*C*H$_3$CH$_2$), 34.4 (CH$_3$*C*H$_2$), 47.4 (*C*HCHCO_2Me), 53.2 (*C*HCN), 56.5 (OCH_3), 77.2 (*C*HCO_2CH_3), 128.5, 129.8, 131.2, 132.1, (Ar*C*H), 169.4 (CN), 170.5, 172.3 (N*C*O), and 176.1 (CO_2Me); MS (EI) *m/z* (%): 300 (M^+, 10); HRMS-ESI required for $C_{29}H_{25}NO_3$: 300.1011; found: 300.1108.

(1*R*,3a*S*,6a*R*)-Methyl 5-benzyl-4,6-dioxo-3-phenyl-1,3a,4,5,6,6a-hexahydro-pyrrolo[3,4-*c*]pyrrole-1-carboxylate (7ad): Colourless oil; $[\alpha]_D^{20}$ +21º (*c* 0.7, CH_2Cl_2, 50% *ee* HPLC); HPLC (Chiralpak OD-H; 1 mL/min; *n*-hexane/*i*PrOH, 80:20; λ = 220 nm): t_{Rmaj} 17, t_{Rmin} 32 min.; R_f 0.56 (n-hexane/ethyl acetate, 1:1); IR (neat) ν_{max}: 1702, 1655 cm^{-1}; ^{1}H RMN (300 MHz, $CDCl_3$) δ_H: 3.84 (s, 3H, CO_2Me), 4.11-4.02 (m, 1H, C*H*CONBz), 4.58-4.53 (m, 1H, NHC*H*$_2$Ph) 4.61 (d, *J* = 4.5 Hz, 1H, NC*H*$_2$Ph), 4.74 (dd, *J* = 8.5, 2.5 Hz, 1H, C*H*CN), 5.24 (t, *J* = 2.9 Hz,1H, C*H*CO_2Me), 7.55-7.38 (m, 5H, Ar*H*), 7.84-7.78 (m, 3H, ArC*H*), and 8.20-8.11 (m, 2H, ArC*H*); ^{13}C RMN ($CDCl_3$) δ_c: 42.91 (N*C*H$_2$Ph), 47.36 (*C*HCHCO_2Me), 53.15 (*C*HCN), 56.41 (OCH_3), 76.64 (*C*HCO_2CH_3), 127.0, 128.1, 128.4, 128.6, 128.7, 128.9, 132.0, 135.0, (Ar*C*), 169.24 (*C*N), 170.34, 172.17 (2xN*C*O), and 175.88 (*C*O_2Me); MS (EI) *m/z* (%): 362.1 (M^+, 100); HRMS-ESI required for $C_{21}H_{18}N_2O_4$: 362.1235; found: 362.1246.

(1*R*,3a*S*,6a*R*)-Methyl 5-(4-acetoxyphenyl)-4,6-dioxo-3-phenyl-1,3a,4,5,6,6a-hexahydropyrrolo[3,4-*c*]pyrrole-1-carboxylate (7ae) [25]**:** Pale yellow oil; $[\alpha]_D^{20}$ = +37° (*c* 0.7, CH_2Cl_2, 99% *ee* HPLC); HPLC (Chiralpak AD-H; 1 mL/min; *n*-hexane/*i*PrOH, 50:50; λ = 220 nm) t_{Rmaj} 60, t_{Rmin} 67 min. R_f 0.56 (*n*-hexane/ethyl acetate, 1:1); IR (neat) ν_{max}: 1716, 1684 cm^{-1}; ^{1}H RMN (300 MHz, $CDCl_3$) δ_H: 2.60 (s, 3H, CH_3CO), 3.87 (s, 3H, OMe), 4.30 (dd, *J*= 8.6, 2.7 Hz, 1H, C*H*CONAr), 4.94 (dd, *J* = 8.6, 2.7 Hz, 1H, C*H*CN), 5.41 (t, *J* = 2.7 Hz, 1H, C*H*CO_2Me), 7.59-7.43 (m, 5H, Ar*H*), 8.01(d, *J* = 8.7 Hz, 2H, Ar*H*), and 8.20 (d, *J* = 6.7 Hz, 2H, Ar*H*); ^{13}C RMN ($CDCl_3$) δ_c: 26.7 (CH_3CO), 47.5 (*C*HCHCO_2Me), 53.3 (*C*HCN), 56.4 (OCH_3), 77.23 (*C*HCO_2CH_3), 126.3, 128.6, 128.6, 129.8, 131.0, 132.3, 135.2, 136.9 (Ar*C*), 169.1 (CN), 170.2, 171.02 (2xN*C*O), 174.9 (CO_2Me), and 196.9 ($COCH_3$); MS (EI) *m/z* (%): 406 (M^+, 0.28); HRMS-ESI required for $C_{21}H_{18}N_2O_4$: 406.1284; found: 406.1278.

(1*R*,3a*S*,6a*R*)-Methyl 5-(4-bromophenyl)-4,6-dioxo-3-phenyl-1,3a,4,5,6,6a-hexahydropyrrolo[3,4-*c*]pyrrole-1-carboxylate (7af) [25]**:** Pale yellow oil; $[\alpha]_D^{20}$ = +30° (*c* 0.7, CH_2Cl_2, 99% *ee* HPLC); HPLC (Chiralpak AD-H; 1 mL/min; *n*-hexane/*i*PrOH, 50:50; λ = 220 nm): t_{Rmaj} 60, t_{Rmin} 67 min. R_f 0.56 (*n*-hexane/ethyl acetate, 1:1); IR (neat) ν_{max}: 1714, 1654 cm^{-1}; ^{1}H RMN (300 MHz, $CDCl_3$) δ_H: 3.84 (s, 3H, CO_2Me), 4.25 (dd, *J* = 8.6, 2.7 Hz, 1H, C*H*CONAr), 4.90 (dd, *J* = 8.6, 2.7 Hz, 1H, C*H*CN), 5.37 (t, *J* = 2.7 Hz, 1H, C*H*CO_2Me), 7.64-7.36 (m, 7H, Ar*H*), 8.28-8.12 (m, 2H, Ar*H*); ^{13}C RMN ($CDCl_3$) δ_c: 26.7 (CH_3CO), 47.4 (*C*HCHCO_2Me), 53.2 (*C*HCN), 56.3 (OCH_3), 76.9 (*C*HCO_2CH_3), 136.9, 132.3, 132.1, 131.6, 129.7, 128.9, 128.4, 128.1, 127.7, 127.6, 127.0 (Ar*C*H), 169.1 (CN), 170.2, 171.0 (2xN*C*O), and 176.0 (CO_2Me); MS (EI) *m/z* (%): 428.0, 426.0 (M^+, 8); HRMS-ESI required for $C_{20}H_{15}BrNO_3$: 427.2481; found: 427.2498.

(1*R*,3a*S*,6a*R*)-Methyl 4,6-dioxo-5-phenyl-3-(*p*-tolyl)-1,3a,4,5,6,6a-hexahydro-pyrrolo[3,4-*c*]pyrrole-1-carboxylate (7ba) [25]**:** Pale yellow oil; $[\alpha]_D^{20}$ = +50° (*c* 1.1, CH_2Cl_2, 99% *ee* HPLC); HPLC (Chiralpak AD-H; 1 mL/min; *n*-hexane/*i*PrOH, 85:15; λ = 220 nm) t_{Rmaj} 53, t_{Rmin} 49 min. R_f 0.56 (*n*-hexane/ethyl acetate, 1:1); IR (neat) ν_{max}: 1715, 1654 cm^{-1}; ^{1}H RMN (300 MHz, $CDCl_3$) δ_H: 2.40 (s, 3H, C*H*$_3$Ar), 3.85 (s, 3H, CO_2*Me*), 4.24 (dd, *J* = 8.6, 2.7 Hz, 1H, C*H*CONPh), 4.92-4.85 (m, 1H, C*H*CN), 5.38 (deform t, *J* = 2.7 Hz, 1H, C*H*CO_2Me), 7.31-7.19 (m, 5H, Ar*H*), 7.54-7.31 (m, 3H, Ar*H*), 8.16-8.03 (m, 1H, Ar*H*); ^{13}C RMN ($CDCl_3$) δ_c: 21.6 (C*H*$_3$Ar), 47.5 (*C*HCHCO_2Me), 53.2 (*C*HCN), 56.4 (OCH_3), 77.0 (*C*HCO_2CH_3), 126.3, 127.6, 128.1, 128.5, 128.7 128.9, 132.0, 142.7 (Ar*C*), 169.2 (CN), 170.3, 171.6 (2xN*C*O), and 175.4 (CO_2Me); MS (EI) *m/z* (%): 362 (M^+, 28); HRMS-ESI required for $C_{21}H_{18}N_2O_4$: 362.1247; found: 362.1248.

(1*R*,3a*S*,6a*R*)-Methyl 3-(4-chlorophenyl)-4,6-dioxo-5-phenyl-1,3a,4,5,6,6a-hexahydropyrrolo[3,4-*c*]pyrrole-1-carboxylate (7ca) [25]**:** Pale yellow oil; $[\alpha]_D^{20}$ = +17° (*c* 1.1, CH_2Cl_2, 98% *ee* HPLC); HPLC (Chiralpak AD-H; 1 mL/min; *n*-hexane/*i*PrOH, 70:30; λ = 220 nm): t_{Rmaj} 28, t_{Rmin} 49 min. R_f 0.56 (*n*-hexane/ethyl acetate, 1:1); IR (neat) ν_{max}: 1716, 1683 cm^{-1}; ^{1}H RMN (400 MHz, $CDCl_3$) δ_H: 3.87 (s, 3H, CO_2Me), 4.28 (dd, *J* = 8.6, 2.7 Hz, 1H, C*H*CONPh), 4.85 (m, 1H, C*H*CN), 5.41 (app t, *J* = 2.7 Hz, 1H, C*H*CO_2Me), 7.52-7.34 (m, 8H, Ar*H*), 8.26-8.20 (m, 1H, Ar*H*); ^{13}C RMN ($CDCl_3$) δ_c: 47.4 (*C*HCHCO_2Me), 53.4 (*C*HCN), 56.4 (OCH_3), 77.2 (*C*HCO_2CH_3), 125.1, 126.3, 126.6, 127.5, 128.2, 129.2, 129.3, 129.9, (Ar*C*H), 168.2 (CN), 170.1, 171.3 (2xN*C*O), and 175.1 (CO_2Me); MS (EI) *m/z* (%): 382 (M^+, 1); HRMS-ESI required for $C_{29}H_{25}NO_3$: 382.0711; found: 382.0705.

(1*R*,3a*S*,6a*R*)-Methyl 4,6-dioxo-5-phenyl-3-(thien-2-yl)-1,3a,4,5,6,6a-hexahydro-pyrrolo[3,4-*c*]pyrrole-1-carboxylate (7da) [25]**:** Colourless oil; $[\alpha]_D^{20}$ = +37° (*c* 0.7,

CH_2Cl_2, 95% *ee* HPLC); HPLC (Chiralpak AD-H; 1 mL/min; *n*-hexane/*i*PrOH, 70:30; λ = 220 nm) t_{Rmaj} 44, t_{Rmin} 34 min. R_f 0.56 (*n*-hexane/ethyl acetate, 1:1); IR (neat) ν_{max}: 1714, 1654 cm^{-1}; ^1H RMN (400 MHz, $CDCl_3$) δ_H: 3.86 (s, 3H, CO_2Me), 4.25 (dd, *J* = 8.6, 2.7 Hz, 1H, C*H*CONPh), 4.78 (dd, *J* = 8.6, 2.7, 1H, C*H*CN), 5.37 (app t, *J* = 2.7 Hz, 1H, C*H*CO_2Me), 7.15 (dd, *J* = 5.0, 3.8 Hz, 1H, SCHC*H*), 7.44 (m, 6H, Ar*H*), 8.11 (d, *J* = 5.0 Hz, 1H, CHS); ^{13}C RMN ($CDCl_3$) δ_c: 47.9 (*C*HCHCO_2Me), 53.2 (*C*HCN), 57.0 (OCH_3), 76.8 (*C*HCO_2CH3), 125.4, 126.3, 126.9, 127.1, 127.6, 127.9, 128.8, 134.3 (Ar*C*), 163.4 (CN), 170.3, 171.4 (N*C*O), 175.1 (CO_2Me); MS (EI) *m/z* (%): 354.1 (M^+, 22); HRMS-ESI required for $C_{18}H_{14}SN_2O_3$: 354.0674; found: 354.0684.

(1*R*,3a*S*,6a*R*)-*N*-benzyl-4,6-dioxo-3,5-diphenyl-1,3a,4,5,6,6a-hexahydro-pyrrolo[3,4-c]pyrrole-1-carboxamide (8aa) [25]**:** Colourless oil; $[\alpha]_D^{20}$ = +17º (*c* 0.7, CH_2Cl_2, 96% *ee* HPLC); HPLC (Chiralpak AD-H; 1 mL/min; *n*-hexane/*i*PrOH, 70:30; λ = 220 nm) t_{Rmaj} 21, t_{Rmin} 38 min. R_f 0.56 (*n*-hexane/ethyl acetate, 1:1); IR (neat) ν_{max}: 1711, 1669 cm^{-1}; ^1H RMN (300 MHz, $CDCl_3$) δ_H: 4.38 (dd, *J* = 14.7, 5.3 Hz, 1H, NHC*H_2*Ph), 4.50 (dd, *J* = 8.7, 2.8 Hz, 1H C*H*CONPh), 4.72-4.62 (m, 1H, NC*H_2*Ph), 4.83 (dd, *J* = 8.7, 2.8 Hz, 1H, C*H*CN), 5.31 (t, *J* = 2.8 Hz, 1H, C*H*CO_2Me), 6.86 (m, 1H, N*H*), 7.53-7.43 (m, 9H, Ar*H*), and 8.19-8.14 (m, 6H, Ar*H*); ^{13}C RMN ($CDCl_3$) δ_c: 36.6 (*C*H_2NH), 46.8 (*C*HCHCO_2Me), 51.9 (*C*HCN), 56.0 (OCH_3), 77.2 (*C*HCO_2CH_3), 126.3, 127.6, 127.8, 128.3, 128.6, 128.7, 129.1, 129.2, 129.7, 131.5, 132.2, (Ar*C*H), 162.8, 165.4 (CN, CONH), 168.2 and 169.0 (N*C*O). MS (EI) *m/z* (%): 423 (M^+, 47); HRMS-ESI required for $C_{26}H_{21}N_3O_3$: 423.1602; found: 423.1583.

(2*R*,3*R*)-3-*tert*-Butyl 2-methyl 2-methyl-5-phenyl-3,4-dihydro-2*H*-pyrrole-2,3-dicarboxylate (11) [5,6].

(1*R*,3*S*,3a*S*,6a*R*)-Methyl 4,6-dioxo-3,5-diphenyloctahydropyrrolo[3,4-*c*]pyrrole-1-carboxylate (13) [29,30].

(1*R*,3*R*,3a*S*,6a*R*)-Methyl 4,6-dioxo-3,5-diphenyloctahydropyrrolo[3,4-*c*]pyrrole-1-carboxylate (14) [25]**:** Colourless oil; $[\alpha]_D^{20}$ = –8º (*c* 0.7, CH_2Cl_2); R_f 0.51 (*n*-hexane/ethyl acetate, 1:5); IR (neat) ν_{max}: 1714 cm^{-1}. ^1H RMN (400 MHz, $CDCl_3$) δ_H: 1.60 (br. s, 1H, N*H*), 3.52 (m, 1H, C*H*CHPh), 3.84 (s, 3H, CO_2Me), 3.85 (d, *J* = 2.4 Hz, 1H, C*H*CHCO_2Me), 4.54 (s, 1H, C*H*CO_2Me), 4.75 (d, *J* = 9.0 Hz, 1H, C*H*Ph), 7.38 (m, 8H, Ar*H*), 7.17 (m, 2H, Ar*H*). ^{13}C RMN ($CDCl_3$) δ_c: 48.5 (CO_2Me), 52.7 (PhCH*C*H), 60.4 (CO_2MeCH*C*H), 61.8 (CO_2Me*C*H), 63.4 (Ph*C*H), 126.1, 127.1, 127.4, 128.1, 128.4, 129.0, 131.7, 137.5 (Ar*C*), 172.9, 173.9 (2xNCO), 176.5 (CO_2Me). MS (EI) *m/z* (%): 350 (M^+, 21); HRMS-ESI required for $C_{20}H_{18}N_2O_4$: 350.3679; found: 350.3670.

4. Cycloaddition of NMM with 7aa. Synthesis of polycyclic structure 15.

A solution of **7aa** (87 mg, 0.25 mmol) and AgTFA (3 mg, 0.012 mmol) in toluene (2 mL) was submitted to a microwave radiation (75 W) for 1h at 120 ºC. Solvent was evaporated and the crude residue was purified by flash chromatography yielding pale yellow prisms. Mp: 250 ºC; $[\alpha]_D^{20}$ = –30º (*c* 1, CH_2Cl_2, 99% *ee* HPLC); HPLC (Chiralpak AD; 1 mL/min; *n*-hexane/*i*PrOH, 60:40; λ = 220 nm) t_{Rmaj} 28, t_{Rmin} 21 min. R_f 0.3 (*n*-hexane/ethyl acetate, 1:9); IR (CH_2Cl_2) ν_{max}: 3045, 1702 cm^{-1}. ^1H NMR (400 MHz, $CDCl_3$) δ_H: 1.75 (s, 1H, NH), 2.88 (s, 3H, NMe), 3.45, 3.58 (2m, 4H, 4xC*H*CO_2Me), 4.00 (s, 3H, CO_2Me), 7.17-7.07 (m, 4H, Ph), and 7.44-725 (m, 6H, Ar*H*); ^{13}C NMR ($CDCl_3$) δ_c: 25.4 (NMe), 52.3, 52.8 (2x*C*HCONMe), 52.9 (CO_2Me), 53.7, 53.8 (2x*C*HCONPh), 72.6, 75.2 (q., CCO_2Me, Ph*C*), 126.0, 128.8, 129.0, 129.7, 130.3, 131.1, 132.0, 133.7 (PhC), 167.4 (*C*O_2Me) 171,5, 172.5, 172.8, 173.5 (4xNCO); MS (EI) *m/z* (%): 459 (M^+, 1); HRMS-ESI required for $C_{25}H_{21}N_3O_6$: 459.1430; found: 459.1439.

5. Computational data

Table 1. Total electronic energies[a] (E, in a.u.), thermal corrections to Gibbs free energies[b](TCGFE, in a.u.), and number of imaginary frequencies[c] (NIMAG) of all stationary points discussed in the main text for the ethereal system.

Structure	E	TCGFE	NIMAG(ν)
**5a**	-551.911206	0.108555	0
**NPM**	-589.991535	0.113260	0
**[{(S$_a$)-Binap-Au}$_2$]-5aa**	-4236.847170	1.357812	0
**[{(S$_a$)-Binap-Au}$_2$]-5aa-b**	-4236.843405	1.354717	0
**TS$_{NMP}$*up***	-4826.856597	1.491039	1 (-261.2728)
**TS$_{NMP}$*down***	-4826.858264	1.496986	1 (-349.5224)
**TS$_{NMP}$*up*-b**	-4826.833709	1.495947	1 (-322.2863)
**TS$_{NMP}$*down*-b**	-4826.860985	1.500072	1 (-353.7280)
**[{(S$_a$)-Binap-Au}$_2$]-9**	-4826.898723	1.497138	0
**9’**	-1141.945819	0.250590	0
**Ylide-I**	-852.705878	0.229632	0
**Ylide-II**	-893.219721	0.270140	0
**[{(S$_a$)-Binap-Au}$_2$]-I**	-4276.128453	1.384178	0
**[{(S$_a$)-Binap-Au}$_2$]-Ib**	-4276.123234	1.382661	0
**tert-Bu Acrylate**	-424.0685862	0.143811	0
**TS10**	-4700.210453	1.548466	1 (-361.6730)
**TS10ent**	-4700.208708	1.553944	1 (-367.3943)
**TS10exo**	-4700.191352	1.546897	1 (-411.5175)
**TS10exo-ent**	-4700.186737	1.549827	1 (-407.1185)
**TS11**	-4700.187216	1.544521	1 (-334.5296)
**TS11ent**	-4700.196605	1.553337	1 (-306.2290)

[a]Computed at the M06/Lanl2dz//ONIOM(B3LYP/Lanl2dz:UFF) level of theory. [b,c]Computed at 218.15 K at the ONIOM(B3LYP/Lanl2dz:UFF) level of theory. [d]If NIMAG=1, the imaginary frequency ν (in parentheses) is given in cm^{-1}

6.Cartesian coordinates

Optimized at the ONIOM(B3LYP/Lanl2dz:UFF level) of all the stationary points discussed in the main text.

5a

Center Number	Atomic Number	Atomic Type	Coordinates (Angstroms) X	Y	Z
1	8	0	-1.443041	-0.843828	0.001700
2	6	0	-2.796657	-0.388917	0.002669
3	6	0	-2.707945	1.144035	0.002163
4	7	0	-1.263094	1.461143	0.001002
5	6	0	-0.617839	0.335322	0.000489
6	1	0	-3.203214	1.559022	0.887681
7	1	0	-3.204569	1.558535	-0.882825
8	6	0	0.828804	0.109533	-0.000808
9	6	0	1.354720	-1.202393	-0.001022
10	6	0	2.746853	-1.394712	-0.002263
11	6	0	3.614741	-0.285486	-0.003296
12	6	0	3.088334	1.023600	-0.003081
13	6	0	1.700211	1.224317	-0.001838
14	1	0	0.676640	-2.049842	-0.000222
15	1	0	3.152508	-2.402982	-0.002428
16	1	0	4.691368	-0.437702	-0.004258
17	1	0	3.758817	1.879057	-0.003877
18	1	0	1.276777	2.224514	-0.001650
19	8	0	-3.753709	-1.149972	0.003611

NPM

Center Number	Atomic Number	Atomic Type	Coordinates (Angstroms) X	Y	Z
1	6	0	1.593618	1.141132	0.220081
2	6	0	3.016105	0.662336	0.128334
3	6	0	3.016053	-0.662457	-0.128362
4	6	0	1.593526	-1.141138	-0.220130
5	7	0	0.767297	0.000029	-0.000042
6	6	0	-0.673271	0.000052	-0.000003
7	8	0	1.209293	-2.300490	-0.445360
8	8	0	1.209486	2.300495	0.445433
9	1	0	3.848761	-1.339833	-0.261271
10	1	0	3.848870	1.339635	0.261279
11	6	0	-1.375384	1.102215	-0.530459
12	6	0	-2.780744	1.098720	-0.521235
13	6	0	-3.489718	-0.000006	0.000029
14	6	0	-2.780686	-1.098713	0.521257
15	6	0	-1.375327	-1.102151	0.530440
16	1	0	-0.835528	1.953844	-0.927896
17	1	0	-3.318195	1.952769	-0.925451
18	1	0	-4.576730	-0.000029	0.000036
19	1	0	-3.318093	-1.952791	0.925469
20	1	0	-0.835422	-1.953775	0.927832

[{(S_a)-Binap-Au}$_2$]-5aa

Center Number	Atomic Number	Atomic Type	Coordinates (Angstroms) X	Y	Z
1	6	0	2.022923	-1.229845	-0.291851
2	6	0	0.564630	-1.336094	0.003868
3	6	0	0.089799	-1.180472	1.250641
4	6	0	1.010453	-0.902471	2.362680

5	6	0	0.535462	-0.832850	3.759262
6	6	0	1.391505	-0.530517	4.749196
7	6	0	2.740021	-0.251201	4.487981
8	6	0	3.238038	-0.300300	3.240006
9	6	0	2.359901	-0.673260	2.097942
10	6	0	2.863565	-0.815542	0.702603
11	6	0	4.269470	-0.433091	0.413799
12	6	0	4.564017	0.751668	-0.189971
13	6	0	5.899076	1.084953	-0.503689
14	6	0	6.926781	0.266868	-0.227264
15	6	0	6.684453	-1.011422	0.461381
16	6	0	5.376895	-1.354370	0.804187
17	6	0	5.162468	-2.621459	1.550148
18	6	0	6.208465	-3.412266	1.850980
19	6	0	7.518659	-3.069852	1.481231
20	6	0	7.786116	-1.930801	0.818323
21	6	0	4.628149	-4.832222	-0.907642
22	6	0	5.859017	-4.691731	-1.553169
23	6	0	6.114469	-3.558820	-2.329358
24	6	0	5.135913	-2.570484	-2.468876
25	6	0	3.896578	-2.704278	-1.827998
26	6	0	3.652340	-3.838988	-1.034392
27	15	0	2.654453	-1.362996	-2.047388
28	6	0	1.406996	-2.354479	-2.978673
29	6	0	0.654893	-3.395802	-2.412007
30	6	0	-0.260763	-4.112572	-3.190077
31	6	0	-0.412971	-3.815261	-4.545623
32	6	0	0.357393	-2.806734	-5.127055
33	6	0	1.267796	-2.085732	-4.348644
34	15	0	3.256745	1.993030	-0.573122
35	6	0	3.792492	3.425964	0.427344
36	6	0	3.268723	3.563896	1.718382
37	6	0	3.620816	4.660566	2.509340
38	6	0	4.510415	5.619412	2.017194
39	6	0	5.053026	5.477266	0.736552
40	6	0	4.699398	4.381825	-0.057630
41	6	0	3.454004	2.508726	-2.318402
42	6	0	4.125662	1.693301	-3.243218
43	6	0	4.285773	2.112239	-4.566965
44	6	0	3.786718	3.351606	-4.974193
45	6	0	3.117191	4.168116	-4.060010
46	6	0	2.932524	3.740189	-2.741796
47	1	0	2.584836	2.821063	2.107326
48	1	0	3.204493	4.766398	3.502779
49	1	0	4.783207	6.469114	2.629444
50	1	0	5.747417	6.216568	0.358957
51	1	0	5.132514	4.286819	-1.043784
52	1	0	2.403476	4.379542	-2.047475
53	1	0	2.732750	5.129114	-4.375337
54	1	0	3.918556	3.679040	-5.997240
55	1	0	4.803866	1.478747	-5.275247
56	1	0	4.530821	0.737008	-2.947675
57	1	0	0.750240	-3.638252	-1.370425
58	1	0	-0.856467	-4.897261	-2.743649
59	1	0	-1.124642	-4.368081	-5.145061
60	1	0	0.247140	-2.580445	-6.179569
61	1	0	1.861599	-1.308638	-4.813724
62	1	0	6.114514	2.024993	-0.993429
63	1	0	7.937980	0.558063	-0.494770
64	1	0	8.809209	-1.687989	0.548061
65	1	0	8.331068	-3.737467	1.738491
66	1	0	6.036370	-4.337661	2.385752
67	1	0	4.164480	-2.926846	1.846832
68	1	0	4.285974	-0.068899	3.082781
69	1	0	3.393114	0.011082	5.310562
70	1	0	1.029980	-0.486357	5.768575
71	1	0	-0.505675	-1.022496	3.986755
72	1	0	-0.977271	-1.221851	1.439320
73	1	0	-0.139714	-1.483375	-0.799338
74	1	0	6.618260	-5.454955	-1.442907
75	1	0	4.438314	-5.699860	-0.289130
76	1	0	2.734748	-3.944677	-0.477990
77	1	0	5.345877	-1.697569	-3.073593
78	1	0	7.071116	-3.445751	-2.822494
79	79	0	-2.065318	-2.906732	0.088713
80	79	0	0.897283	1.626148	0.123689
81	6	0	-2.225514	0.189855	-1.846465

82	6	0	-1.288862	-0.166041	-2.940571
83	6	0	-0.589792	0.758640	-3.609593
84	6	0	-0.737004	2.183233	-3.285460
85	6	0	-0.093791	3.217640	-4.123995
86	6	0	-0.303895	4.519489	-3.867353
87	6	0	-1.126526	4.931988	-2.811470
88	6	0	-1.746081	4.043800	-2.015525
89	6	0	-1.560112	2.580492	-2.227985
90	6	0	-2.300885	1.553080	-1.437489
91	6	0	-3.260790	2.044716	-0.426811
92	6	0	-2.869160	2.439141	0.818930
93	6	0	-3.906547	2.980510	1.750268
94	6	0	-5.172953	3.175213	1.358511
95	6	0	-5.567927	2.899942	0.050314
96	6	0	-4.651053	2.374284	-0.870967
97	6	0	-5.097959	2.226025	-2.281305
98	6	0	-6.366510	2.507664	-2.625280
99	6	0	-7.296108	2.970744	-1.683464
100	6	0	-6.951982	3.176191	-0.400735
101	6	0	-7.088323	-0.298192	-0.062471
102	6	0	-6.936404	-0.060303	1.306094
103	6	0	-5.673467	-0.157366	1.894713
104	6	0	-4.562173	-0.483811	1.113127
105	6	0	-4.699354	-0.694372	-0.264855
106	6	0	-5.976834	-0.624413	-0.846285
107	15	0	-3.226984	-1.236986	-1.199727
108	6	0	-3.849658	-2.070395	-2.704336
109	6	0	-3.769844	-3.466746	-2.787407
110	6	0	-4.178315	-4.127666	-3.949605
111	6	0	-4.663503	-3.395794	-5.036797
112	6	0	-4.738054	-2.002138	-4.962871
113	6	0	-4.327713	-1.339759	-3.802258
114	15	0	-1.092038	2.513211	1.357442
115	6	0	-0.744949	4.313029	1.303469
116	6	0	0.253432	4.778107	0.436104
117	6	0	0.572925	6.136978	0.386864
118	6	0	-0.099796	7.042238	1.210596
119	6	0	-1.087655	6.586534	2.087842
120	6	0	-1.407505	5.226083	2.139477
121	6	0	-1.028175	2.163895	3.157757
122	6	0	-0.017112	2.739278	3.943700
123	6	0	0.004997	2.537107	5.326510
124	6	0	-0.979959	1.755511	5.934916
125	6	0	-1.977876	1.162906	5.158125
126	6	0	-1.992616	1.350473	3.772861
127	1	0	0.779482	4.090476	-0.210283
128	1	0	1.342639	6.486636	-0.289091
129	1	0	0.148643	8.094940	1.174216
130	1	0	-1.598841	7.286577	2.735912
131	1	0	-2.145555	4.892840	2.851619
132	1	0	-2.763853	0.872315	3.189602
133	1	0	-2.739590	0.557164	5.629096
134	1	0	-0.965699	1.603173	7.006211
135	1	0	0.782627	2.991157	5.926766
136	1	0	0.739301	3.365642	3.492764
137	1	0	-3.384566	-4.043111	-1.956076
138	1	0	-4.112673	-5.206270	-4.009497
139	1	0	-4.977052	-3.907739	-5.937285
140	1	0	-5.107170	-1.434645	-5.807278
141	1	0	-4.374462	-0.263022	-3.765320
142	1	0	-3.649217	3.225878	2.772317
143	1	0	-5.889840	3.572107	2.067127
144	1	0	-7.692664	3.547786	0.301321
145	1	0	-8.309602	3.180088	-2.001182
146	1	0	-6.676631	2.382901	-3.654985
147	1	0	-4.403189	1.907762	-3.048614
148	1	0	-2.385014	4.414604	-1.221355
149	1	0	-1.270969	5.990866	-2.637960
150	1	0	0.168325	5.267425	-4.491108
151	1	0	0.541546	2.926900	-4.955177
152	1	0	0.058170	0.459039	-4.426102
153	1	0	-1.196788	-1.203241	-3.235936
154	1	0	-7.796821	0.196805	1.909890
155	1	0	-8.067934	-0.228170	-0.517099
156	1	0	-6.124969	-0.810850	-1.898253
157	1	0	-3.591329	-0.579060	1.578887
158	1	0	-5.555068	0.021449	2.955301

159	6	0	-0.898729	-4.351878	2.522717
160	6	0	-0.259711	-5.283476	0.583103
161	6	0	0.559368	-5.920755	1.537482
162	8	0	0.075923	-5.250775	2.845293
163	6	0	-1.577569	-3.635638	3.595105
164	6	0	-2.808880	-2.954604	3.414144
165	6	0	-1.003037	-3.671330	4.893918
166	6	0	-3.427081	-2.300357	4.490663
167	1	0	-3.309796	-2.982101	2.449363
168	6	0	-1.627531	-3.017077	5.966236
169	1	0	-0.077753	-4.219273	5.039749
170	6	0	-2.839369	-2.324011	5.772178
171	1	0	-4.383448	-1.803891	4.343943
172	1	0	-1.176847	-3.056461	6.954502
173	7	0	-1.099409	-4.333849	1.186678
174	1	0	-0.308779	-5.524953	-0.462665
175	8	0	1.463091	-6.763441	1.583114
176	1	0	-3.328893	-1.831984	6.608637

[{(S_a)-Binap-Au}$_2$]-5aa-b

Center Number	Atomic Number	Atomic Type	Coordinates (Angstroms) X	Y	Z
1	6	0	1.138854	2.146795	-0.122724
2	6	0	-0.158816	1.508164	-0.483908
3	6	0	-0.333912	0.918836	-1.675357
4	6	0	0.762220	0.882322	-2.653405
5	6	0	0.551900	0.378680	-4.026974
6	6	0	1.579084	0.346025	-4.893327
7	6	0	2.868403	0.749011	-4.516285
8	6	0	3.124217	1.223255	-3.284422
9	6	0	2.025234	1.352694	-2.290886
10	6	0	2.207338	2.007457	-0.963214
11	6	0	3.554813	2.524813	-0.594754
12	6	0	4.328082	1.890796	0.330624
13	6	0	5.581037	2.417948	0.708611
14	6	0	6.063096	3.559067	0.193387
15	6	0	5.294569	4.285162	-0.830522
16	6	0	4.066913	3.768319	-1.245890
17	6	0	3.340322	4.488688	-2.324316
18	6	0	3.853467	5.616706	-2.847849
19	6	0	5.078327	6.140612	-2.406447
20	6	0	5.796725	5.532008	-1.446659
21	6	0	1.422822	6.579346	-0.535062
22	6	0	2.292905	7.343838	0.245377
23	6	0	2.863867	6.794082	1.395476
24	6	0	2.559023	5.482412	1.770341
25	6	0	1.682770	4.708851	0.997125
26	6	0	1.126118	5.262861	-0.169850
27	15	0	1.356922	2.976334	1.531064
28	6	0	-0.356099	3.192412	2.176231
29	6	0	-1.387663	3.813497	1.449587
30	6	0	-2.642940	4.005287	2.033639
31	6	0	-2.887750	3.563974	3.335201
32	6	0	-1.867803	2.951066	4.065142
33	6	0	-0.604236	2.780213	3.492641
34	15	0	3.813656	0.298732	1.091891
35	6	0	5.243722	-0.779321	0.714444
36	6	0	5.381883	-1.254817	-0.596059
37	6	0	6.426292	-2.122290	-0.926454
38	6	0	7.348119	-2.509009	0.049490
39	6	0	7.231276	-2.019616	1.353433
40	6	0	6.185355	-1.153348	1.687320
41	6	0	3.772266	0.501451	2.910971
42	6	0	3.892411	1.764613	3.514321
43	6	0	3.860870	1.887032	4.906283
44	6	0	3.720106	0.750855	5.706116
45	6	0	3.601814	-0.508164	5.113683
46	6	0	3.610547	-0.631330	3.720982
47	1	0	4.672214	-0.959054	-1.358386
48	1	0	6.519461	-2.495615	-1.938068
49	1	0	8.156368	-3.181873	-0.205878

50	1	0	7.952508	-2.310897	2.105970
51	1	0	6.126966	-0.775714	2.697405
52	1	0	3.514870	-1.612221	3.273955
53	1	0	3.498925	-1.388891	5.734014
54	1	0	3.703635	0.845837	6.784064
55	1	0	3.949980	2.863340	5.364889
56	1	0	4.004045	2.658852	2.919878
57	1	0	-1.241608	4.138502	0.434613
58	1	0	-3.425757	4.501988	1.478698
59	1	0	-3.864132	3.705446	3.780281
60	1	0	-2.052104	2.618089	5.078245
61	1	0	0.186388	2.328438	4.078816
62	1	0	6.177955	1.896865	1.446785
63	1	0	7.027800	3.935636	0.519622
64	1	0	6.743788	5.956023	-1.127176
65	1	0	5.453179	7.053306	-2.851894
66	1	0	3.307276	6.136359	-3.624790
67	1	0	2.386137	4.124123	-2.689187
68	1	0	4.137179	1.518951	-3.032469
69	1	0	3.674700	0.675749	-5.234954
70	1	0	1.413382	-0.017413	-5.899366
71	1	0	-0.431582	0.040841	-4.339829
72	1	0	-1.288947	0.478456	-1.926759
73	1	0	-0.978891	1.495789	0.218662
74	1	0	2.531575	8.358409	-0.045914
75	1	0	0.996265	6.998539	-1.437129
76	1	0	0.496831	4.676127	-0.823132
77	1	0	3.009810	5.067293	2.662874
78	1	0	3.543752	7.383880	1.996399
79	79	0	-3.324135	1.113052	-0.763788
80	79	0	1.872934	-0.909681	0.115233
81	6	0	-1.846078	-1.507366	1.153179
82	6	0	-1.555117	-0.496571	2.201829
83	6	0	-0.688591	-0.739547	3.192788
84	6	0	0.021778	-2.024197	3.264232
85	6	0	0.846305	-2.365901	4.442878
86	6	0	1.458087	-3.559591	4.514944
87	6	0	1.318432	-4.513570	3.498164
88	6	0	0.564855	-4.276165	2.410391
89	6	0	-0.128432	-2.968072	2.243867
90	6	0	-1.051794	-2.687360	1.106186
91	6	0	-1.243010	-3.755964	0.101463
92	6	0	-0.351749	-3.965179	-0.910092
93	6	0	-0.593016	-5.104779	-1.846766
94	6	0	-1.610700	-5.958123	-1.669292
95	6	0	-2.471497	-5.830197	-0.579776
96	6	0	-2.307084	-4.780014	0.335099
97	6	0	-3.191856	-4.759002	1.529129
98	6	0	-4.161628	-5.678493	1.672731
99	6	0	-4.355665	-6.693580	0.725324
100	6	0	-3.566705	-6.803112	-0.357135
101	6	0	-4.999028	-4.157269	-2.131489
102	6	0	-4.242635	-4.114241	-3.304007
103	6	0	-3.201465	-3.192356	-3.427891
104	6	0	-2.907470	-2.319741	-2.374831
105	6	0	-3.651847	-2.363756	-1.188557
106	6	0	-4.712539	-3.281852	-1.080117
107	15	0	-3.329590	-1.115670	0.113331
108	6	0	-4.780144	-1.187462	1.233144
109	6	0	-6.025129	-0.772348	0.737081
110	6	0	-7.157254	-0.796838	1.555963
111	6	0	-7.052272	-1.225540	2.880950
112	6	0	-5.814406	-1.628633	3.387718
113	6	0	-4.680056	-1.606699	2.570571
114	15	0	1.235873	-3.015268	-1.078424
115	6	0	2.473119	-4.195523	-0.419111
116	6	0	3.260151	-3.805501	0.673377
117	6	0	4.238024	-4.662628	1.183495
118	6	0	4.439793	-5.916068	0.601332
119	6	0	3.665981	-6.309728	-0.493992
120	6	0	2.687830	-5.451933	-1.007094
121	6	0	1.683350	-2.942732	-2.856193
122	6	0	3.033720	-2.914762	-3.238554
123	6	0	3.385089	-2.896820	-4.591338
124	6	0	2.390518	-2.903488	-5.572360
125	6	0	1.043920	-2.906793	-5.199718
126	6	0	0.692577	-2.907241	-3.846766

127	1	0	3.110609	-2.841753	1.138184
128	1	0	4.838760	-4.353958	2.029337
129	1	0	5.198427	-6.579809	0.995187
130	1	0	3.830509	-7.276560	-0.951702
131	1	0	2.125242	-5.762261	-1.873550
132	1	0	-0.349122	-2.887612	-3.574520
133	1	0	0.272246	-2.892816	-5.958348
134	1	0	2.662592	-2.892449	-6.619708
135	1	0	4.428417	-2.883351	-4.878608
136	1	0	3.815709	-2.927392	-2.492804
137	1	0	-6.117893	-0.430562	-0.286250
138	1	0	-8.114381	-0.476932	1.164911
139	1	0	-7.928553	-1.241207	3.515901
140	1	0	-5.732725	-1.955463	4.416288
141	1	0	-3.736975	-1.914367	2.993663
142	1	0	0.060127	-5.261944	-2.695817
143	1	0	-1.748369	-6.769237	-2.374069
144	1	0	-3.736835	-7.607628	-1.066646
145	1	0	-5.151360	-7.411790	0.877013
146	1	0	-4.803599	-5.640034	2.543667
147	1	0	-3.056600	-4.013082	2.300835
148	1	0	0.473597	-5.053537	1.659302
149	1	0	1.822314	-5.466786	3.596431
150	1	0	2.064573	-3.794286	5.380384
151	1	0	0.952296	-1.654450	5.255761
152	1	0	-0.540720	-0.002038	3.973219
153	1	0	-2.098958	0.443325	2.201608
154	1	0	-4.465308	-4.792433	-4.117364
155	1	0	-5.806165	-4.871755	-2.034932
156	1	0	-5.310613	-3.342418	-0.182581
157	1	0	-2.105986	-1.605220	-2.488440
158	1	0	-2.626921	-3.150932	-4.343762
159	6	0	-4.388981	3.938516	-1.167445
160	6	0	-2.465027	3.615192	-2.277966
161	6	0	-2.805648	4.948243	-2.586935
162	8	0	-4.129076	5.113543	-1.809146
163	6	0	-5.598084	3.822642	-0.355876
164	6	0	-5.742300	2.881429	0.695632
165	6	0	-6.661694	4.728171	-0.607608
166	6	0	-6.924374	2.825988	1.449623
167	1	0	-4.915408	2.226565	0.962291
168	6	0	-7.842524	4.665943	0.147247
169	1	0	-6.544716	5.468550	-1.392668
170	6	0	-7.984485	3.712323	1.175466
171	1	0	-7.012204	2.112954	2.266040
172	1	0	-8.649843	5.362666	-0.062184
173	7	0	-3.421916	3.034478	-1.429891
174	1	0	-1.628267	3.075942	-2.681367
175	8	0	-2.355608	5.897666	-3.240177
176	1	0	-8.897756	3.671887	1.763097

$\mathbf{TS_{NMP}}$***up***

Center Number	Atomic Number	Atomic Type	Coordinates (Angstroms) X	Y	Z
1	7	0	3.181885	2.855977	0.907360
2	6	0	3.530774	3.075642	2.181544
3	8	0	3.482856	4.420775	2.506018
4	6	0	3.174961	5.151996	1.260097
5	6	0	3.288698	4.110792	0.211377
6	6	0	3.693274	2.095180	3.259515
7	6	0	4.504878	0.947855	3.077457
8	6	0	4.599774	-0.004422	4.106459
9	6	0	3.896836	0.178086	5.314370
10	6	0	3.117888	1.337353	5.506364
11	6	0	3.024351	2.301211	4.489213
12	8	0	3.008122	6.363446	1.253170
13	79	0	2.940023	0.922021	0.237683
14	15	0	2.698027	-1.436582	-0.009063
15	6	0	1.192803	-1.937970	-0.946967
16	6	0	0.845465	-1.069319	-2.091301
17	6	0	0.104589	-1.517587	-3.115424

18	6	0	-0.394286	-2.908683	-3.151130
19	6	0	-0.169016	-3.777875	-2.077244
20	6	0	0.544644	-3.286305	-0.872432
21	6	0	-0.638387	-5.182403	-2.204988
22	6	0	-1.283973	-5.581617	-3.314387
23	6	0	-1.530472	-4.699663	-4.376295
24	6	0	-1.120434	-3.420243	-4.335641
25	6	0	0.537933	-4.101684	0.366254
26	6	0	-0.465392	-4.011418	1.284327
27	6	0	-0.376178	-4.845658	2.522514
28	6	0	0.650240	-5.681550	2.733349
29	6	0	1.651907	-5.843254	1.776303
30	6	0	1.608745	-5.120608	0.575458
31	6	0	2.622229	-5.441850	-0.459999
32	6	0	3.605727	-6.320022	-0.199593
33	6	0	3.687195	-6.984464	1.032411
34	6	0	2.768501	-6.792616	1.994649
35	15	0	-2.034664	-3.078058	0.961374
36	6	0	-2.982729	-3.031880	2.531472
37	6	0	-4.315949	-3.473734	2.602996
38	6	0	-5.016948	-3.406354	3.810638
39	6	0	-4.397341	-2.894135	4.952699
40	6	0	-3.081444	-2.432393	4.883527
41	6	0	-2.386059	-2.477967	3.671716
42	6	0	2.600731	-2.170868	1.666971
43	6	0	1.612021	-1.708975	2.546074
44	6	0	1.570488	-2.162945	3.868008
45	6	0	2.510636	-3.090658	4.319032
46	6	0	3.492199	-3.566884	3.448241
47	6	0	3.539810	-3.110772	2.127569
48	79	0	-2.313249	-0.863822	-0.105221
49	15	0	-4.062288	0.458894	-1.226526
50	6	0	-3.712051	0.872111	-2.971460
51	6	0	-4.065867	2.112924	-3.525478
52	6	0	-3.744279	2.406715	-4.853859
53	6	0	-3.078644	1.462246	-5.639018
54	6	0	-2.739008	0.220070	-5.098087
55	6	0	-3.056434	-0.075050	-3.769098
56	6	0	-4.723180	1.942715	-0.366110
57	6	0	-3.983787	2.612856	0.560733
58	6	0	-4.553737	3.823503	1.220441
59	6	0	-5.840911	4.232154	0.872053
60	6	0	-6.590933	3.448987	-0.124456
61	6	0	-6.028307	2.370043	-0.691718
62	6	0	-3.799892	4.652484	2.197853
63	6	0	-4.362125	5.757388	2.719853
64	6	0	-5.660366	6.158029	2.366788
65	6	0	-6.398220	5.455028	1.489176
66	6	0	-2.588869	2.216849	0.871247
67	6	0	-1.535040	2.579730	0.007284
68	6	0	-0.141922	2.212639	0.374474
69	6	0	0.132269	1.659271	1.566382
70	6	0	-0.941746	1.373306	2.532517
71	6	0	-2.275510	1.594897	2.181903
72	6	0	-3.320924	1.288322	3.192737
73	6	0	-2.969569	0.871466	4.421894
74	6	0	-1.624924	0.685577	4.777685
75	6	0	-0.631302	0.901930	3.899771
76	15	0	-1.915917	3.639040	-1.471065
77	6	0	-0.351723	3.558635	-2.439349
78	6	0	0.395697	4.705202	-2.763995
79	6	0	1.497766	4.607586	-3.618845
80	6	0	1.843215	3.376086	-4.180343
81	6	0	1.089833	2.239351	-3.884896
82	6	0	-0.007891	2.332684	-3.025351
83	6	0	-1.775380	5.261955	-0.618645
84	6	0	-2.830953	6.174250	-0.747994
85	6	0	-2.798153	7.392774	-0.064173
86	6	0	-1.709933	7.706601	0.754180
87	6	0	-0.651557	6.803645	0.886194
88	6	0	-0.681487	5.584037	0.203924
89	6	0	-5.474943	-0.701911	-1.243699
90	6	0	-5.861214	-1.297166	-0.034601
91	6	0	-6.893848	-2.238377	-0.010102
92	6	0	-7.549115	-2.587916	-1.193474
93	6	0	-7.174823	-1.992762	-2.401405
94	6	0	-6.140555	-1.051953	-2.429964

95	6	0	-2.981716	-4.230777	-0.092011
96	6	0	-3.579911	-3.752649	-1.264912
97	6	0	-4.337587	-4.608847	-2.068106
98	6	0	-4.490119	-5.950879	-1.708903
99	6	0	-3.885865	-6.436367	-0.545060
100	6	0	-3.133988	-5.579082	0.264430
101	1	0	-5.350174	-1.043832	0.886229
102	1	0	-7.181665	-2.700870	0.925156
103	1	0	-8.346439	-3.319451	-1.174693
104	1	0	-7.683765	-2.263912	-3.317251
105	1	0	-5.868311	-0.607240	-3.376232
106	1	0	-2.789528	-1.039682	-3.358890
107	1	0	-2.227424	-0.511754	-5.708522
108	1	0	-2.828615	1.691818	-6.666626
109	1	0	-4.008272	3.368728	-5.273509
110	1	0	-4.572137	2.863125	-2.936641
111	1	0	0.118809	5.681202	-2.394497
112	1	0	2.072994	5.491574	-3.861856
113	1	0	2.689699	3.304265	-4.850975
114	1	0	1.349191	1.289115	-4.333059
115	1	0	-0.606722	1.453331	-2.828951
116	1	0	-6.600317	1.825895	-1.432427
117	1	0	-7.594667	3.749198	-0.409534
118	1	0	-7.398193	5.789824	1.230829
119	1	0	-6.074320	7.054969	2.809601
120	1	0	-3.798867	6.356030	3.424439
121	1	0	-2.788493	4.387140	2.487140
122	1	0	-4.372606	1.412563	2.955629
123	1	0	-3.741268	0.666836	5.153119
124	1	0	-1.384491	0.347739	5.777637
125	1	0	0.399330	0.745951	4.201764
126	1	0	1.153525	1.427585	1.836059
127	1	0	0.679005	2.427054	-0.297601
128	1	0	-1.687675	8.648266	1.286959
129	1	0	0.190830	7.047755	1.519923
130	1	0	0.148698	4.901857	0.314025
131	1	0	-3.688195	5.932500	-1.363950
132	1	0	-3.621082	8.088987	-0.160833
133	1	0	-3.455692	-2.720557	-1.559716
134	1	0	-4.799476	-4.233478	-2.972058
135	1	0	-5.073090	-6.614945	-2.333665
136	1	0	-4.004011	-7.475832	-0.268040
137	1	0	-2.682580	-5.962473	1.169669
138	1	0	-1.375530	-2.099399	3.623305
139	1	0	-2.604066	-2.027990	5.766372
140	1	0	-4.939248	-2.847079	5.888333
141	1	0	-6.042235	-3.749670	3.860102
142	1	0	-4.827176	-3.856787	1.731911
143	1	0	-1.157031	-4.808064	3.270531
144	1	0	0.680094	-6.260503	3.648536
145	1	0	2.849843	-7.336018	2.931491
146	1	0	4.496888	-7.682502	1.203033
147	1	0	4.342707	-6.532379	-0.963802
148	1	0	2.558555	-4.998486	-1.444063
149	1	0	-0.470846	-5.895887	-1.404757
150	1	0	-1.627921	-6.605484	-3.388235
151	1	0	-2.059525	-5.060070	-5.249300
152	1	0	-1.318103	-2.765807	-5.178231
153	1	0	-0.094513	-0.857856	-3.953722
154	1	0	1.251711	-0.065101	-2.127077
155	1	0	2.476189	-3.443125	5.341525
156	1	0	4.213798	-4.295328	3.794372
157	1	0	4.296259	-3.515928	1.474452
158	1	0	0.873572	-0.998725	2.205592
159	1	0	0.814598	-1.792440	4.546837
160	1	0	5.121778	0.844648	2.187028
161	1	0	2.440516	3.205287	4.637254
162	1	0	5.251288	-0.865209	3.979123
163	1	0	2.598887	1.495090	6.448013
164	1	0	2.711587	4.237733	-0.698699
165	1	0	3.979671	-0.558639	6.109555
166	6	0	5.055804	4.261086	-0.300590
167	6	0	5.010916	3.431117	-1.577576
168	1	0	5.122631	5.336697	-0.464197
169	6	0	5.946050	3.562517	0.595119
170	6	0	6.232623	2.266181	0.070099
171	1	0	6.372070	3.934693	1.514843

172	8	0	4.464889	3.761977	-2.651404
173	7	0	5.637326	2.215464	-1.293312
174	8	0	6.812744	1.256225	0.560746
175	6	0	5.846984	1.179902	-2.256610
176	6	0	7.111244	0.566354	-2.378112
177	6	0	7.329711	-0.379596	-3.394930
178	6	0	6.298263	-0.722391	-4.291312
179	6	0	5.031358	-0.122462	-4.152567
180	6	0	4.802482	0.822723	-3.137953
181	1	0	7.904882	0.832142	-1.689353
182	1	0	8.309658	-0.839888	-3.492513
183	1	0	6.481662	-1.440508	-5.086514
184	1	0	4.232056	-0.368760	-4.848153
185	1	0	3.843521	1.324492	-3.061411
186	6	0	4.149235	-2.154487	-0.862733
187	6	0	5.426298	-1.968945	-0.312802
188	6	0	4.019027	-2.852468	-2.073423
189	6	0	6.543371	-2.560752	-0.908298
190	1	0	5.550700	-1.392840	0.595508
191	6	0	5.137323	-3.444870	-2.667207
192	1	0	3.058411	-2.955860	-2.556912
193	6	0	6.396713	-3.311773	-2.077172
194	1	0	7.522685	-2.433870	-0.465816
195	1	0	5.028488	-4.001389	-3.588949
196	1	0	7.262169	-3.767780	-2.539815

TS$_{NMP}$*down*

Center Number	Atomic Number	Atomic Type	Coordinates (Angstroms) X	Y	Z
1	7	0	-2.890134	2.666213	-0.861033
2	6	0	-3.477363	3.801782	-0.433593
3	8	0	-2.968067	4.906287	-1.122214
4	6	0	-2.119326	4.397512	-2.198105
5	6	0	-2.339809	2.949253	-2.143664
6	6	0	-4.291082	4.042488	0.748434
7	6	0	-5.157819	3.042877	1.255065
8	6	0	-5.907873	3.283922	2.415018
9	6	0	-5.816074	4.526619	3.072606
10	6	0	-4.979572	5.536102	2.553073
11	6	0	-4.224501	5.302597	1.394238
12	8	0	-1.501070	5.146643	-2.952064
13	79	0	-2.811082	0.825205	0.033788
14	15	0	-2.648829	-1.329411	1.077355
15	6	0	-3.823300	-1.309256	2.484261
16	6	0	-5.080398	-0.722470	2.290235
17	6	0	-6.009299	-0.675407	3.332753
18	6	0	-5.687144	-1.216467	4.579585
19	6	0	-4.435794	-1.804288	4.782770
20	6	0	-3.504767	-1.851369	3.740522
21	6	0	-0.982546	-1.720349	1.806544
22	6	0	-0.545738	-0.776793	2.866905
23	6	0	0.533904	-1.009375	3.622744
24	6	0	1.345194	-2.214719	3.414852
25	6	0	1.037692	-3.097287	2.376045
26	6	0	-0.138961	-2.827318	1.496645
27	6	0	1.847729	-4.344569	2.274917
28	6	0	2.847179	-4.580752	3.142258
29	6	0	3.146168	-3.684587	4.176474
30	6	0	2.446467	-2.550276	4.342723
31	6	0	-0.500027	-3.875913	0.519511
32	6	0	0.147113	-4.012713	-0.675069
33	6	0	-0.228714	-5.160462	-1.557059
34	6	0	-1.103951	-6.095291	-1.164474
35	6	0	-1.689549	-6.042445	0.098922
36	6	0	-1.401312	-4.982540	0.970484
37	6	0	-1.978928	-5.039441	2.339819
38	6	0	-2.806063	-6.039984	2.688183
39	6	0	-3.132203	-7.067393	1.792064
40	6	0	-2.615795	-7.106609	0.552134
41	15	0	1.592993	-2.962600	-1.189575
42	6	0	1.558751	-2.788643	-3.015175

43	6	0	2.759356	-2.602755	-3.718083
44	6	0	2.756443	-2.522484	-5.113497
45	6	0	1.553576	-2.620642	-5.817237
46	6	0	0.350801	-2.777644	-5.123807
47	6	0	0.351755	-2.847756	-3.727568
48	6	0	-3.264986	-2.645416	-0.034890
49	6	0	-2.854583	-2.655493	-1.374370
50	6	0	-3.334916	-3.630070	-2.254012
51	6	0	-4.245938	-4.589104	-1.805899
52	6	0	-4.695427	-4.559525	-0.484025
53	6	0	-4.215023	-3.587382	0.398061
54	79	0	2.463288	-0.902010	-0.098688
55	15	0	4.455551	0.431916	0.522895
56	6	0	4.793758	0.497233	2.320705
57	6	0	4.902223	1.720376	3.003628
58	6	0	5.163810	1.740382	4.376477
59	6	0	5.329637	0.542670	5.075279
60	6	0	5.224556	-0.676764	4.403007
61	6	0	4.939178	-0.700879	3.034488
62	6	0	4.664779	2.123687	-0.172008
63	6	0	3.655432	2.752261	-0.835009
64	6	0	3.886991	4.107357	-1.413192
65	6	0	5.125676	4.716579	-1.213579
66	6	0	6.170637	3.981213	-0.480730
67	6	0	5.919436	2.748626	-0.011305
68	6	0	2.861162	4.851381	-2.188551
69	6	0	3.129376	6.085958	-2.650397
70	6	0	4.372238	6.699598	-2.427900
71	6	0	5.351913	6.076137	-1.749317
72	6	0	2.321807	2.113958	-0.973242
73	6	0	1.458636	2.051723	0.081994
74	6	0	0.243042	1.199612	-0.030950
75	6	0	-0.081829	0.606634	-1.189865
76	6	0	0.740321	0.821147	-2.389961
77	6	0	1.949530	1.504941	-2.279950
78	6	0	2.783014	1.644387	-3.503588
79	6	0	2.325491	1.196942	-4.686112
80	6	0	1.072047	0.578699	-4.799601
81	6	0	0.292778	0.371318	-3.724528
82	15	0	1.913044	2.894759	1.681515
83	6	0	0.444009	2.742978	2.784780
84	6	0	-0.872930	3.040683	2.390749
85	6	0	-1.919775	2.948405	3.314469
86	6	0	-1.662948	2.585802	4.637335
87	6	0	-0.355761	2.315088	5.042235
88	6	0	0.691835	2.397456	4.121138
89	6	0	1.712718	4.650427	1.169007
90	6	0	2.621986	5.592505	1.668032
91	6	0	2.530188	6.934331	1.287003
92	6	0	1.527863	7.344199	0.404328
93	6	0	0.623435	6.410304	-0.106838
94	6	0	0.719952	5.065919	0.263695
95	6	0	5.844416	-0.480503	-0.239131
96	6	0	5.699676	-0.916181	-1.562532
97	6	0	6.709024	-1.664354	-2.173931
98	6	0	7.876624	-1.968969	-1.469529
99	6	0	8.038362	-1.517195	-0.156658
100	6	0	7.027949	-0.771327	0.458708
101	6	0	3.016573	-4.092493	-0.945570
102	6	0	4.026727	-3.715709	-0.048829
103	6	0	5.147267	-4.528225	0.138854
104	6	0	5.270459	-5.722585	-0.574317
105	6	0	4.275578	-6.100195	-1.480203
106	6	0	3.154184	-5.286620	-1.671196
107	1	0	4.798678	-0.682605	-2.115366
108	1	0	6.585069	-2.008665	-3.192509
109	1	0	8.658326	-2.548908	-1.942682
110	1	0	8.947784	-1.744462	0.384302
111	1	0	7.181703	-0.419884	1.468710
112	1	0	4.853219	-1.652943	2.527182
113	1	0	5.357513	-1.604240	4.944310
114	1	0	5.539659	0.559419	6.136816
115	1	0	5.242248	2.685614	4.897796
116	1	0	4.780988	2.661133	2.487260
117	1	0	-1.105977	3.314998	1.376550
118	1	0	-2.932745	3.155317	3.009378
119	1	0	-2.476549	2.515515	5.347568

120	1	0	-0.153316	2.037930	6.068582
121	1	0	1.702272	2.188793	4.451219
122	1	0	6.708917	2.230629	0.518605
123	1	0	7.146588	4.431163	-0.326062
124	1	0	6.305347	6.572064	-1.595156
125	1	0	4.545624	7.695586	-2.814995
126	1	0	2.368690	6.620868	-3.204720
127	1	0	1.887161	4.413427	-2.380772
128	1	0	3.758337	2.117218	-3.459924
129	1	0	2.935061	1.319568	-5.572293
130	1	0	0.732378	0.249010	-5.773005
131	1	0	-0.667825	-0.119469	-3.839679
132	1	0	-0.958820	-0.021408	-1.252200
133	1	0	-0.374892	1.012693	0.831273
134	1	0	1.459172	8.382671	0.107736
135	1	0	-0.139754	6.724283	-0.806893
136	1	0	0.046945	4.355548	-0.191275
137	1	0	3.406163	5.285357	2.348644
138	1	0	3.237508	7.655886	1.674668
139	1	0	3.944453	-2.797015	0.513701
140	1	0	5.919783	-4.230208	0.836062
141	1	0	6.139375	-6.351525	-0.430619
142	1	0	4.378673	-7.018753	-2.043203
143	1	0	2.419971	-5.579069	-2.404566
144	1	0	-0.587356	-2.950189	-3.208453
145	1	0	-0.583612	-2.833962	-5.666843
146	1	0	1.551785	-2.559903	-6.897688
147	1	0	3.687326	-2.388884	-5.649061
148	1	0	3.700418	-2.543296	-3.189506
149	1	0	-5.336885	-0.298199	1.330955
150	1	0	-6.977609	-0.218762	3.173747
151	1	0	-6.406182	-1.180139	5.387638
152	1	0	-4.186492	-2.223367	5.749009
153	1	0	-2.546629	-2.310070	3.925351
154	1	0	0.205786	-5.257511	-2.543280
155	1	0	-1.342323	-6.910765	-1.836634
156	1	0	-2.880962	-7.922031	-0.114473
157	1	0	-3.806274	-7.851989	2.111459
158	1	0	-3.225481	-6.057763	3.686122
159	1	0	-1.731965	-4.284023	3.073903
160	1	0	1.644275	-5.080227	1.504458
161	1	0	3.429583	-5.488351	3.045951
162	1	0	3.952717	-3.916645	4.860151
163	1	0	2.686786	-1.883035	5.164724
164	1	0	0.790682	-0.326161	4.423743
165	1	0	-1.147678	0.100626	3.074142
166	1	0	-4.616050	-5.344157	-2.486857
167	1	0	-5.411550	-5.294415	-0.140379
168	1	0	-4.569344	-3.594874	1.418862
169	1	0	-2.162160	-1.911600	-1.737484
170	1	0	-3.006372	-3.638634	-3.284898
171	1	0	-5.286154	2.111198	0.714131
172	1	0	-3.584383	6.077387	0.983937
173	1	0	-6.586114	2.520513	2.786662
174	1	0	-4.922779	6.501893	3.047820
175	1	0	-1.646535	2.266783	-2.608763
176	1	0	-6.405787	4.715067	3.965926
177	6	0	-5.046048	3.405528	-2.718808
178	6	0	-5.912813	2.393073	-2.180839
179	1	0	-5.327654	4.447379	-2.745173
180	6	0	-3.909455	2.804665	-3.325278
181	6	0	-4.110858	1.298643	-3.237014
182	1	0	-3.424349	3.185593	-4.221396
183	8	0	-6.985168	2.473988	-1.540361
184	7	0	-5.295489	1.080602	-2.530004
185	8	0	-3.315569	0.418355	-3.651866
186	6	0	-5.909529	-0.191721	-2.298747
187	6	0	-5.769913	-1.230623	-3.247180
188	6	0	-6.419498	-2.459209	-3.041255
189	6	0	-7.200403	-2.676333	-1.889213
190	6	0	-7.334002	-1.641048	-0.944925
191	6	0	-6.699414	-0.402260	-1.145680
192	1	0	-5.168982	-1.073533	-4.134851
193	1	0	-6.321595	-3.243881	-3.787778
194	1	0	-7.707467	-3.626283	-1.740401
195	1	0	-7.955607	-1.783755	-0.064021
196	1	0	-6.868923	0.415386	-0.456263

TS$_{NMP}$*up-b*

Center Number	Atomic Number	Atomic Type	Coordinates (Angstroms) X	Y	Z
1	7	0	4.652847	-2.077073	-1.913491
2	6	0	4.111023	-3.311835	-2.277110
3	8	0	5.075184	-3.964181	-3.107047
4	6	0	6.351320	-3.296104	-2.910568
5	6	0	6.012290	-2.191058	-2.020994
6	6	0	2.719680	-3.512236	-2.797267
7	6	0	2.448132	-3.053862	-4.109489
8	6	0	1.215722	-3.331905	-4.722299
9	6	0	0.245669	-4.089992	-4.036935
10	6	0	0.514202	-4.547597	-2.734321
11	6	0	1.740261	-4.253667	-2.108096
12	8	0	7.390327	-3.678084	-3.451211
13	79	0	3.796625	-0.485733	-0.959972
14	15	0	3.232057	1.579395	0.107915
15	6	0	4.484267	1.888690	1.408683
16	6	0	5.842399	1.784025	1.073652
17	6	0	6.828108	2.049897	2.028205
18	6	0	6.463756	2.415728	3.326093
19	6	0	5.113419	2.492350	3.676194
20	6	0	4.125721	2.211835	2.727564
21	6	0	1.576729	1.648643	0.931149
22	6	0	1.281484	0.503793	1.819566
23	6	0	0.382162	0.587112	2.808556
24	6	0	-0.350952	1.840814	3.067688
25	6	0	-0.243649	2.927932	2.191814
26	6	0	0.673161	2.846302	1.023424
27	6	0	-0.994029	4.167604	2.527752
28	6	0	-1.719960	4.235459	3.656555
29	6	0	-1.793880	3.154779	4.545813
30	6	0	-1.160716	1.995407	4.296072
31	6	0	0.776545	4.009755	0.113016
32	6	0	-0.073710	4.182484	-0.935690
33	6	0	0.123324	5.364657	-1.827213
34	6	0	1.038085	6.304978	-1.553687
35	6	0	1.828254	6.222306	-0.407120
36	6	0	1.711980	5.123205	0.456985
37	6	0	2.503341	5.146208	1.713641
38	6	0	3.362317	6.150675	1.957964
39	6	0	3.519873	7.215405	1.059390
40	6	0	2.801015	7.289253	-0.073846
41	15	0	-1.588508	3.148278	-1.192518
42	6	0	-1.867905	2.979089	-2.996360
43	6	0	-3.171675	2.838254	-3.494785
44	6	0	-3.394523	2.718970	-4.869529
45	6	0	-2.315389	2.736194	-5.756693
46	6	0	-1.013170	2.863962	-5.267349
47	6	0	-0.790437	2.972680	-3.891524
48	6	0	3.417467	2.921263	-1.120279
49	6	0	2.760694	2.794870	-2.350352
50	6	0	2.969048	3.736388	-3.363572
51	6	0	3.840923	4.807184	-3.155641
52	6	0	4.513977	4.931587	-1.938484
53	6	0	4.310130	3.990268	-0.925076
54	79	0	-2.287089	1.097363	0.024295
55	15	0	-4.323072	-0.067310	0.788392
56	6	0	-4.528139	-0.323062	2.588249
57	6	0	-5.082767	-1.503328	3.111856
58	6	0	-5.221063	-1.665027	4.493167
59	6	0	-4.825891	-0.644378	5.360240
60	6	0	-4.293275	0.539544	4.845852
61	6	0	-4.140662	0.698026	3.464738
62	6	0	-4.776961	-1.607212	-0.104034
63	6	0	-3.850834	-2.332853	-0.793393
64	6	0	-4.300344	-3.512402	-1.592350
65	6	0	-5.641003	-3.893031	-1.534932
66	6	0	-6.575983	-3.083740	-0.738337
67	6	0	-6.134262	-1.996045	-0.089368

68	6	0	-3.391343	-4.308466	-2.455301
69	6	0	-3.856476	-5.378385	-3.124439
70	6	0	-5.201573	-5.769483	-3.038252
71	6	0	-6.084924	-5.083632	-2.291584
72	6	0	-2.392097	-2.018672	-0.728158
73	6	0	-1.640705	-2.369346	0.358255
74	6	0	-0.179465	-2.075854	0.366039
75	6	0	0.424124	-1.514233	-0.689298
76	6	0	-0.354781	-1.150122	-1.880880
77	6	0	-1.732209	-1.385168	-1.907308
78	6	0	-2.474311	-1.014012	-3.140518
79	6	0	-1.814883	-0.515273	-4.201034
80	6	0	-0.429098	-0.298668	-4.168944
81	6	0	0.297710	-0.568905	-3.072295
82	15	0	-2.466522	-3.071865	1.869457
83	6	0	-1.077819	-3.467594	3.013589
84	6	0	-0.077361	-4.414614	2.725869
85	6	0	0.913477	-4.701484	3.669753
86	6	0	0.917417	-4.047499	4.903597
87	6	0	-0.069503	-3.103701	5.195359
88	6	0	-1.064023	-2.818036	4.256177
89	6	0	-2.902542	-4.754766	1.265899
90	6	0	-4.085734	-5.340154	1.736229
91	6	0	-4.494578	-6.589358	1.260001
92	6	0	-3.723650	-7.262306	0.308963
93	6	0	-2.545678	-6.684144	-0.169513
94	6	0	-2.140704	-5.430744	0.298147
95	6	0	-5.650151	1.090131	0.283075
96	6	0	-5.754542	1.427045	-1.072694
97	6	0	-6.715912	2.346187	-1.501347
98	6	0	-7.586936	2.928031	-0.577334
99	6	0	-7.502003	2.583953	0.774305
100	6	0	-6.538701	1.666765	1.206492
101	6	0	-2.912959	4.307270	-0.683367
102	6	0	-3.648796	4.019111	0.474375
103	6	0	-4.682555	4.864693	0.884076
104	6	0	-4.995150	6.000302	0.133419
105	6	0	-4.272036	6.291042	-1.026633
106	6	0	-3.234082	5.448219	-1.436346
107	1	0	-5.087246	0.976214	-1.796312
108	1	0	-6.784059	2.608115	-2.549260
109	1	0	-8.329606	3.642051	-0.908706
110	1	0	-8.180227	3.031974	1.488870
111	1	0	-6.496271	1.419758	2.256442
112	1	0	-3.737599	1.622258	3.073543
113	1	0	-4.001887	1.334332	5.518776
114	1	0	-4.937957	-0.768290	6.429510
115	1	0	-5.636975	-2.581641	4.890953
116	1	0	-5.396038	-2.308442	2.465518
117	1	0	-0.036213	-4.919397	1.777017
118	1	0	1.686046	-5.423712	3.439466
119	1	0	1.690073	-4.265230	5.629419
120	1	0	-0.064148	-2.592901	6.149463
121	1	0	-1.827224	-2.088340	4.498448
122	1	0	-6.851756	-1.411674	0.472989
123	1	0	-7.626519	-3.353895	-0.690599
124	1	0	-7.121267	-5.404130	-2.246646
125	1	0	-5.534595	-6.639878	-3.589075
126	1	0	-3.177560	-5.953576	-3.741178
127	1	0	-2.347381	-4.040693	-2.546325
128	1	0	-3.548507	-1.153442	-3.203344
129	1	0	-2.365997	-0.267776	-5.099117
130	1	0	0.063546	0.103989	-5.044516
131	1	0	1.368050	-0.387590	-3.073818
132	1	0	1.490643	-1.326517	-0.661348
133	1	0	0.423240	-2.298847	1.233209
134	1	0	-4.042859	-8.227235	-0.062740
135	1	0	-1.956558	-7.197225	-0.918549
136	1	0	-1.250228	-4.983185	-0.115853
137	1	0	-4.696850	-4.824077	2.465805
138	1	0	-5.411498	-7.033406	1.625061
139	1	0	-3.420448	3.141748	1.063843
140	1	0	-5.243848	4.636289	1.780859
141	1	0	-5.799809	6.652114	0.447856
142	1	0	-4.520903	7.165976	-1.613049
143	1	0	-2.708826	5.674695	-2.351801
144	1	0	0.218730	3.052167	-3.526940

145	1	0	-0.175146	2.866455	-5.952170
146	1	0	-2.487535	2.644529	-6.821153
147	1	0	-4.403802	2.616985	-5.246640
148	1	0	-4.016546	2.842399	-2.823134
149	1	0	6.136407	1.511689	0.067675
150	1	0	7.874069	1.976857	1.760281
151	1	0	7.226986	2.624285	4.064403
152	1	0	4.831669	2.754773	4.687638
153	1	0	3.091532	2.254460	3.032470
154	1	0	-0.475168	5.476555	-2.722606
155	1	0	1.146676	7.147702	-2.225627
156	1	0	2.937854	8.133738	-0.742944
157	1	0	4.225890	8.002322	1.292311
158	1	0	3.938463	6.144055	2.874584
159	1	0	2.379830	4.368277	2.454101
160	1	0	-0.959636	5.031503	1.872019
161	1	0	-2.256281	5.146925	3.888203
162	1	0	-2.379962	3.256365	5.450014
163	1	0	-1.240323	1.174121	5.002134
164	1	0	0.227177	-0.268573	3.457788
165	1	0	1.851931	-0.407661	1.701265
166	1	0	3.998666	5.537173	-3.938773
167	1	0	5.190973	5.760490	-1.777589
168	1	0	4.839342	4.114878	0.007979
169	1	0	2.102704	1.954943	-2.528793
170	1	0	2.462558	3.631584	-4.314049
171	1	0	3.216828	-2.514666	-4.657213
172	1	0	1.925900	-4.594625	-1.095653
173	1	0	1.023960	-2.985392	-5.734742
174	1	0	-0.213756	-5.165672	-2.217759
175	1	0	6.705488	-1.430215	-1.697746
176	1	0	-0.695103	-4.338426	-4.522843
177	6	0	4.624868	-4.365049	-0.521386
178	6	0	3.944556	-3.675403	0.624184
179	1	0	4.206403	-5.295611	-0.887134
180	6	0	6.007679	-4.113777	-0.387762
181	6	0	6.241718	-3.231168	0.756515
182	1	0	6.823236	-4.675709	-0.818533
183	8	0	2.720923	-3.727550	0.895960
184	7	0	4.928654	-2.941251	1.344423
185	8	0	7.326329	-2.801906	1.196786
186	6	0	4.707228	-2.158128	2.534157
187	6	0	5.778596	-1.461971	3.150422
188	6	0	5.560824	-0.736911	4.333770
189	6	0	4.283601	-0.661460	4.918426
190	6	0	3.220002	-1.342330	4.300765
191	6	0	3.421738	-2.091511	3.128213
192	1	0	6.772813	-1.520892	2.731353
193	1	0	6.404746	-0.235858	4.801820
194	1	0	4.127340	-0.102033	5.837143
195	1	0	2.228052	-1.312873	4.745495
196	1	0	2.601599	-2.631626	2.678766

TS$_{NMP}$*up-b*

Center Number	Atomic Number	Atomic Type	Coordinates (Angstroms) X	Y	Z
1	6	0	7.709860	-1.011632	-1.076288
2	6	0	7.645383	0.147262	-1.872787
3	6	0	7.061948	1.328237	-1.380670
4	6	0	6.502955	1.354536	-0.080825
5	6	0	6.566453	0.190248	0.723826
6	6	0	7.180956	-0.972572	0.228289
7	7	0	5.886432	2.544926	0.422545
8	6	0	5.796260	2.919598	1.857002
9	6	0	5.105542	4.181839	1.910570
10	6	0	4.720866	4.581941	0.600970
11	6	0	5.320106	3.569338	-0.355960
12	6	0	2.816134	4.148963	0.303265
13	6	0	2.218147	5.139427	1.209205
14	8	0	2.217505	4.455227	2.495870
15	6	0	2.635378	3.135441	2.271327

16	7	0	2.687008	2.882803	0.942377
17	6	0	2.684147	2.247043	3.422436
18	6	0	3.287609	0.966938	3.353951
19	6	0	3.355920	0.156115	4.496090
20	6	0	2.842976	0.614820	5.725537
21	6	0	2.256464	1.894578	5.804797
22	6	0	2.179286	2.708920	4.666255
23	8	0	1.858530	6.308365	1.092242
24	79	0	2.692816	1.089990	-0.050944
25	15	0	2.735773	-1.007604	-1.222094
26	6	0	3.688558	-0.739768	-2.758896
27	6	0	4.286166	0.507095	-2.969342
28	6	0	4.969268	0.771994	-4.159493
29	6	0	5.048302	-0.207931	-5.152133
30	6	0	4.434423	-1.448459	-4.957963
31	6	0	3.747291	-1.710877	-3.768966
32	8	0	6.260692	2.204521	2.774440
33	8	0	5.256184	3.615777	-1.605965
34	6	0	1.103218	-1.664851	-1.819475
35	6	0	0.509004	-0.896124	-2.940645
36	6	0	-0.590285	-1.308361	-3.583255
37	6	0	-1.251048	-2.562718	-3.200246
38	6	0	-0.777033	-3.301528	-2.112468
39	6	0	0.416118	-2.822653	-1.353616
40	6	0	-1.429306	-4.612736	-1.836784
41	6	0	-2.440363	-5.046331	-2.608735
42	6	0	-2.903724	-4.296495	-3.697234
43	6	0	-2.362283	-3.107336	-4.009750
44	6	0	0.957824	-3.720874	-0.313008
45	6	0	0.436988	-3.774850	0.946038
46	6	0	1.018335	-4.757775	1.912026
47	6	0	1.955978	-5.640181	1.541558
48	6	0	2.409036	-5.692135	0.224437
49	6	0	1.919261	-4.788767	-0.729272
50	6	0	2.338057	-4.982946	-2.142577
51	6	0	3.227631	-5.938574	-2.462400
52	6	0	3.765120	-6.794799	-1.490977
53	6	0	3.394715	-6.713047	-0.201711
54	15	0	-1.078023	-2.834583	1.467580
55	6	0	-0.910864	-2.429827	3.249073
56	6	0	-2.058963	-2.319454	4.049520
57	6	0	-1.943732	-2.085805	5.422744
58	6	0	-0.681372	-1.957063	6.006824
59	6	0	0.465351	-2.045592	5.214626
60	6	0	0.351978	-2.261358	3.838108
61	6	0	3.661016	-2.220097	-0.217084
62	6	0	3.455182	-2.213682	1.168382
63	6	0	4.191524	-3.062121	1.998921
64	6	0	5.151947	-3.914797	1.450193
65	6	0	5.385190	-3.907809	0.072526
66	6	0	4.654187	-3.053494	-0.759183
67	79	0	-2.269083	-1.064840	0.179522
68	15	0	-4.431156	-0.094064	-0.546993
69	6	0	-4.893532	-0.490774	-2.273001
70	6	0	-5.005921	0.518517	-3.242551
71	6	0	-5.384574	0.201412	-4.550029
72	6	0	-5.664984	-1.122428	-4.896017
73	6	0	-5.555633	-2.131827	-3.936932
74	6	0	-5.150580	-1.821373	-2.635199
75	6	0	-4.834983	1.677351	-0.237021
76	6	0	-3.933076	2.528579	0.325673
77	6	0	-4.347708	3.922501	0.661195
78	6	0	-5.629888	4.342139	0.308161
79	6	0	-6.539969	3.381194	-0.336209
80	6	0	-6.134468	2.121725	-0.562512
81	6	0	-3.466546	4.891828	1.362572
82	6	0	-3.902299	6.139475	1.614527
83	6	0	-5.186567	6.559928	1.234453
84	6	0	-6.040973	5.729969	0.610282
85	6	0	-2.546415	2.086923	0.621311
86	6	0	-1.624361	1.932009	-0.374894
87	6	0	-0.344602	1.233465	-0.062306
88	6	0	-0.021683	0.895156	1.196906
89	6	0	-0.936478	1.210454	2.304351
90	6	0	-2.192662	1.747886	2.028076
91	6	0	-3.122609	1.964218	3.169590
92	6	0	-2.723585	1.700044	4.426028

93	6	0	-1.441388	1.203086	4.696940
94	6	0	-0.568193	0.945837	3.709283
95	15	0	-2.092876	2.322735	-2.141957
96	6	0	-0.515531	2.422134	-3.094185
97	6	0	0.691776	2.907195	-2.564280
98	6	0	1.835114	2.983458	-3.367531
99	6	0	1.779098	2.597222	-4.707754
100	6	0	0.576632	2.142815	-5.250793
101	6	0	-0.566110	2.064305	-4.449899
102	6	0	-2.384564	4.134266	-1.989819
103	6	0	-3.496637	4.683209	-2.642875
104	6	0	-3.763714	6.052426	-2.554553
105	6	0	-2.917289	6.884342	-1.817800
106	6	0	-1.808712	6.345716	-1.160168
107	6	0	-1.546538	4.974481	-1.235553
108	6	0	-5.661323	-0.948742	0.500650
109	6	0	-5.289014	-1.302165	1.803245
110	6	0	-6.182077	-1.987048	2.631267
111	6	0	-7.458922	-2.310700	2.164508
112	6	0	-7.844482	-1.943051	0.871900
113	6	0	-6.950838	-1.260197	0.040708
114	6	0	-2.357843	-4.147583	1.491156
115	6	0	-3.458138	-4.028325	0.630607
116	6	0	-4.471901	-4.989184	0.638966
117	6	0	-4.396735	-6.075278	1.513797
118	6	0	-3.310123	-6.196318	2.384250
119	6	0	-2.295110	-5.234303	2.378163
120	1	0	-4.304204	-1.048243	2.172546
121	1	0	-5.884284	-2.266109	3.633713
122	1	0	-8.150870	-2.841391	2.805477
123	1	0	-8.835952	-2.187990	0.513686
124	1	0	-7.268340	-0.981166	-0.954498
125	1	0	-5.061069	-2.615462	-1.905671
126	1	0	-5.775476	-3.156860	-4.204745
127	1	0	-5.966868	-1.365975	-5.906349
128	1	0	-5.468765	0.983920	-5.292990
129	1	0	-4.808370	1.550656	-2.994065
130	1	0	0.763504	3.203221	-1.533164
131	1	0	2.768087	3.339674	-2.954203
132	1	0	2.665998	2.653751	-5.325460
133	1	0	0.529113	1.850795	-6.291762
134	1	0	-1.494510	1.718222	-4.887068
135	1	0	-6.834892	1.436480	-1.020372
136	1	0	-7.545735	3.684060	-0.611145
137	1	0	-7.030174	6.079538	0.330990
138	1	0	-5.495448	7.574478	1.451676
139	1	0	-3.245890	6.838472	2.117006
140	1	0	-2.463679	4.609352	1.665442
141	1	0	-4.126656	2.340047	3.004979
142	1	0	-3.406848	1.872577	5.247819
143	1	0	-1.155356	1.008012	5.722571
144	1	0	0.406852	0.540777	3.944514
145	1	0	0.896299	0.354976	1.400297
146	1	0	0.316830	0.937538	-0.861183
147	1	0	-3.126478	7.943766	-1.747202
148	1	0	-1.165135	6.986945	-0.571916
149	1	0	-0.718887	4.576617	-0.670225
150	1	0	-4.159797	4.049021	-3.217238
151	1	0	-4.627477	6.468167	-3.056763
152	1	0	-3.528138	-3.196272	-0.055074
153	1	0	-5.315409	-4.890116	-0.032091
154	1	0	-5.182869	-6.819023	1.522015
155	1	0	-3.259294	-7.030832	3.071541
156	1	0	-1.486283	-5.328252	3.085286
157	1	0	1.249615	-2.312002	3.241819
158	1	0	1.442499	-1.946440	5.666838
159	1	0	-0.592038	-1.782001	7.070991
160	1	0	-2.833114	-2.010874	6.034755
161	1	0	-3.042520	-2.440845	3.618511
162	1	0	4.215075	1.275310	-2.213043
163	1	0	5.576706	-0.003231	-6.074110
164	1	0	4.483801	-2.203501	-5.731743
165	1	0	3.259901	-2.663861	-3.641252
166	1	0	0.687701	-4.773341	2.942252
167	1	0	2.346105	-6.335521	2.274970
168	1	0	3.819772	-7.400173	0.524068
169	1	0	4.483354	-7.547500	-1.790174

170	1	0	3.529597	-6.057336	-3.495231
171	1	0	1.917231	-4.370560	-2.930841
172	1	0	-1.097062	-5.238227	-1.015298
173	1	0	-2.903599	-6.000089	-2.389769
174	1	0	-3.713192	-4.687154	-4.300245
175	1	0	-2.736003	-2.551904	-4.864662
176	1	0	-0.972474	-0.735037	-4.420905
177	1	0	1.002089	0.006662	-3.278220
178	1	0	5.719431	-4.575666	2.092081
179	1	0	6.137886	-4.559846	-0.350989
180	1	0	4.870018	-3.057915	-1.814697
181	1	0	2.725260	-1.544611	1.602364
182	1	0	4.015894	-3.059587	3.066646
183	1	0	3.750262	0.631887	2.430952
184	1	0	1.744770	3.701287	4.724602
185	1	0	3.849245	-0.810947	4.441289
186	1	0	1.873381	2.258968	6.754132
187	1	0	2.921651	-0.004068	6.615646
188	1	0	2.703132	4.242552	-0.766251
189	1	0	5.039827	4.761041	2.819098
190	1	0	4.687287	5.614985	0.263492
191	1	0	7.025731	2.214939	-2.000295
192	1	0	6.208684	0.223600	1.744800
193	1	0	8.067479	0.144757	-2.874851
194	1	0	7.271185	-1.839986	0.878032
195	1	0	8.186583	-1.913607	-1.451669
196	1	0	5.432715	1.737870	-4.312954

[{(S_a)-Binap-Au}$_2$]-9

Center Number	Atomic Number	Atomic Type	Coordinates (Angstroms) X	Y	Z

9’

Center Number	Atomic Number	Atomic Type	Coordinates (Angstroms) X	Y	Z

Ylide-I

Center Number	Atomic Number	Atomic Type	Coordinates (Angstroms) X	Y	Z
1	79	0	0.770605	-0.595654	-0.039703
2	15	0	2.995799	0.272161	0.145609
3	6	0	-2.161255	0.013159	-0.043481
4	6	0	-1.876090	-2.219958	-0.089966
5	6	0	-3.277838	-2.031153	0.027029
6	8	0	-3.413846	-0.520189	0.060858
7	6	0	-2.004181	1.462955	-0.058034
8	6	0	-0.919257	2.119550	-0.696944
9	6	0	-3.012981	2.256730	0.549108
10	6	0	-0.828780	3.519715	-0.698111
11	1	0	-0.178266	1.541978	-1.244683
12	6	0	-2.913054	3.655909	0.550684
13	1	0	-3.861810	1.762870	1.011629
14	6	0	-1.818695	4.296931	-0.065227
15	1	0	-0.003993	4.006639	-1.213677
16	1	0	-3.690375	4.247638	1.026726
17	7	0	-1.236288	-0.975183	-0.123065
18	8	0	-4.283208	-2.754764	0.104491
19	1	0	-1.749677	5.381395	-0.068920

20	6	0	-1.200728	-3.551239	-0.126339
21	1	0	-0.846699	-3.822452	-1.133926
22	1	0	-0.347786	-3.606205	0.566603
23	1	0	-1.923826	-4.317006	0.173667
24	6	0	2.923859	2.058228	0.487941
25	1	0	3.910783	2.424801	0.679004
26	1	0	2.511918	2.567271	-0.358275
27	1	0	2.306582	2.232662	1.344353
28	6	0	3.864780	-0.571420	1.504153
29	1	0	3.905962	-1.621631	1.303503
30	1	0	4.859080	-0.184699	1.586104
31	1	0	3.340183	-0.403880	2.421556
32	6	0	3.889290	-0.011024	-1.414480
33	1	0	4.884119	0.373934	-1.330688
34	1	0	3.929004	-1.060884	-1.617253
35	1	0	3.380039	0.487389	-2.212696

Ylide-II

Center Number	Atomic Number	Atomic Type	Coordinates (Angstroms) X	Y	Z
1	79	0	-0.229699	0.461982	-0.011039
2	15	0	-0.205910	2.829364	-0.138783
3	6	0	1.016762	-2.311510	-0.162384
4	6	0	-1.361228	-2.349028	-0.034263
5	6	0	-2.570179	-1.598127	0.082849
6	8	0	-2.702412	-0.328513	0.190017
7	6	0	2.357959	-1.753494	-0.000967
8	6	0	2.684525	-0.673713	0.867081
9	6	0	3.429769	-2.378775	-0.700250
10	6	0	4.007418	-0.216666	0.987510
11	1	0	1.905861	-0.226113	1.478498
12	6	0	4.750332	-1.922456	-0.576402
13	1	0	3.210179	-3.220106	-1.355603
14	6	0	5.050033	-0.829643	0.263648
15	1	0	4.231323	0.602438	1.668590
16	1	0	5.545492	-2.415833	-1.131497
17	7	0	-0.152209	-1.672662	-0.010815
18	8	0	-3.726650	-2.402940	0.083004
19	1	0	6.073911	-0.477767	0.365699
20	6	0	-1.374084	-3.853685	-0.149943
21	1	0	-0.970502	-4.199773	-1.117028
22	1	0	-0.761785	-4.321745	0.639066
23	1	0	-2.396572	-4.221153	-0.061224
24	6	0	0.957119	3.571392	-1.414724
25	1	0	0.896210	4.666105	-1.403812
26	1	0	1.982049	3.257636	-1.196243
27	1	0	0.687273	3.200951	-2.408252
28	6	0	-1.889692	3.540536	-0.573177
29	1	0	-2.621175	3.199246	0.164825
30	1	0	-1.859356	4.636229	-0.592550
31	1	0	-2.196714	3.162145	-1.552603
32	6	0	0.261094	3.702798	1.459326
33	1	0	0.207309	4.790679	1.334531
34	1	0	-0.423960	3.392713	2.253991
35	1	0	1.278386	3.418878	1.745082
36	1	0	0.985593	-3.362913	-0.441478
37	6	0	-5.001626	-1.699328	0.229100
38	1	0	-5.041667	-1.159228	1.181730
39	1	0	-5.147658	-0.984009	-0.587936
40	1	0	-5.760551	-2.484926	0.197183

[{(S_a)-Binap-Au}$_2$]-I

Center Number	Atomic Number	Atomic Type	Coordinates (Angstroms) X	Y	Z
1	6	0	2.162078	-1.082225	-0.299861
2	6	0	0.708997	-1.293047	-0.036856

3	6	0	0.186392	-1.079342	1.183710
4	6	0	1.050397	-0.657046	2.297980
5	6	0	0.530881	-0.524188	3.673138
6	6	0	1.340638	-0.121768	4.666665
7	6	0	2.681448	0.208336	4.426929
8	6	0	3.217695	0.113135	3.197895
9	6	0	2.390878	-0.364734	2.057297
10	6	0	2.940503	-0.556305	0.689646
11	6	0	4.318865	-0.086104	0.406591
12	6	0	4.532272	1.105635	-0.214956
13	6	0	5.841191	1.520480	-0.540743
14	6	0	6.920043	0.769237	-0.266280
15	6	0	6.761557	-0.520851	0.426744
16	6	0	5.480815	-0.938351	0.786818
17	6	0	5.347094	-2.214936	1.533697
18	6	0	6.438906	-2.949753	1.812613
19	6	0	7.722603	-2.535077	1.423549
20	6	0	7.917498	-1.379286	0.763717
21	6	0	4.399450	-4.910527	-0.437208
22	6	0	5.653889	-4.992667	-1.046494
23	6	0	6.070781	-3.988681	-1.923966
24	6	0	5.229535	-2.907272	-2.201366
25	6	0	3.965020	-2.821660	-1.603645
26	6	0	3.559311	-3.826027	-0.705734
27	15	0	2.921316	-1.354660	-1.984575
28	6	0	1.651030	-2.180553	-3.034565
29	6	0	0.872550	-3.272838	-2.617669
30	6	0	-0.016989	-3.885207	-3.509569
31	6	0	-0.123320	-3.426846	-4.823571
32	6	0	0.666179	-2.359868	-5.253072
33	6	0	1.553922	-1.746292	-4.365049
34	15	0	3.136474	2.236484	-0.629494
35	6	0	3.570952	3.748239	0.301038
36	6	0	3.041789	3.908902	1.587510
37	6	0	3.315701	5.064530	2.323641
38	6	0	4.131256	6.061215	1.780957
39	6	0	4.677944	5.898866	0.504459
40	6	0	4.402627	4.744275	-0.235014
41	6	0	3.282251	2.682827	-2.398951
42	6	0	3.997760	1.871132	-3.293900
43	6	0	4.107079	2.232254	-4.639745
44	6	0	3.511994	3.409411	-5.099420
45	6	0	2.797557	4.220968	-4.215247
46	6	0	2.665083	3.849505	-2.873952
47	1	0	2.412725	3.138949	2.015114
48	1	0	2.895636	5.187092	3.313579
49	1	0	4.343144	6.956625	2.350694
50	1	0	5.314423	6.668513	0.087484
51	1	0	4.836537	4.635174	-1.219412
52	1	0	2.099008	4.482494	-2.203510
53	1	0	2.336015	5.132419	-4.571828
54	1	0	3.603277	3.691908	-6.140133
55	1	0	4.658658	1.601921	-5.325184
56	1	0	4.474134	0.961231	-2.959194
57	1	0	0.926686	-3.635938	-1.607267
58	1	0	-0.629178	-4.714477	-3.188262
59	1	0	-0.816050	-3.899375	-5.507928
60	1	0	0.590872	-2.007081	-6.273382
61	1	0	2.168027	-0.925907	-4.716028
62	1	0	5.992810	2.466290	-1.042976
63	1	0	7.909212	1.119668	-0.544900
64	1	0	8.921559	-1.079396	0.479694
65	1	0	8.574117	-3.158369	1.665111
66	1	0	6.326059	-3.884060	2.347723
67	1	0	4.372287	-2.568919	1.852922
68	1	0	4.259062	0.381389	3.054764
69	1	0	3.296825	0.547250	5.250571
70	1	0	0.947412	-0.039102	5.671635
71	1	0	-0.505202	-0.753341	3.882485
72	1	0	-0.878936	-1.186631	1.350909
73	1	0	0.042265	-1.562234	-0.839515
74	1	0	6.307249	-5.827661	-0.829583
75	1	0	4.085479	-5.677700	0.258411
76	1	0	2.618458	-3.760509	-0.180095
77	1	0	5.565636	-2.132086	-2.878570
78	1	0	7.047120	-4.046707	-2.387184
79	79	0	-1.844155	-3.017414	0.170781

80	79	0	0.789520	1.716764	0.100006
81	6	0	-2.215949	0.097912	-1.781052
82	6	0	-1.260954	-0.200298	-2.876367
83	6	0	-0.662122	0.765148	-3.584166
84	6	0	-0.934412	2.178372	-3.298521
85	6	0	-0.387178	3.241965	-4.167979
86	6	0	-0.699953	4.527846	-3.938058
87	6	0	-1.550840	4.894972	-2.887774
88	6	0	-2.091651	3.977014	-2.068241
89	6	0	-1.780679	2.529681	-2.244152
90	6	0	-2.418576	1.462170	-1.416807
91	6	0	-3.404351	1.900022	-0.406049
92	6	0	-3.027749	2.365776	0.819315
93	6	0	-4.090126	2.865810	1.746224
94	6	0	-5.374211	2.941303	1.371478
95	6	0	-5.765554	2.585087	0.082012
96	6	0	-4.821948	2.106854	-0.838038
97	6	0	-5.272891	1.889707	-2.237633
98	6	0	-6.565021	2.055308	-2.569023
99	6	0	-7.517675	2.462601	-1.624500
100	6	0	-7.174181	2.731896	-0.353482
101	6	0	-7.003170	-0.739121	0.086332
102	6	0	-6.844661	-0.438002	1.441621
103	6	0	-5.566563	-0.405922	2.004107
104	6	0	-4.447116	-0.667741	1.209840
105	6	0	-4.593967	-0.943722	-0.156049
106	6	0	-5.884042	-1.001158	-0.710476
107	15	0	-3.101551	-1.394860	-1.107836
108	6	0	-3.691562	-2.277860	-2.598249
109	6	0	-3.544027	-3.669715	-2.662066
110	6	0	-3.922761	-4.366769	-3.813084
111	6	0	-4.447827	-3.675643	-4.908256
112	6	0	-4.592304	-2.286706	-4.853094
113	6	0	-4.211242	-1.588339	-3.703633
114	15	0	-1.251646	2.578670	1.326216
115	6	0	-1.015501	4.389109	1.155666
116	6	0	-0.034196	4.857412	0.270993
117	6	0	0.200080	6.227528	0.133276
118	6	0	-0.542604	7.141289	0.884354
119	6	0	-1.515140	6.683421	1.777545
120	6	0	-1.749318	5.311836	1.918048
121	6	0	-1.153795	2.351953	3.145082
122	6	0	-0.182651	3.046579	3.883630
123	6	0	-0.135903	2.929807	5.275687
124	6	0	-1.054780	2.114296	5.940573
125	6	0	-2.009688	1.401828	5.211723
126	6	0	-2.049974	1.506606	3.818325
127	1	0	0.543359	4.162563	-0.321216
128	1	0	0.957344	6.579418	-0.555460
129	1	0	-0.360686	8.202904	0.779022
130	1	0	-2.081346	7.391413	2.368745
131	1	0	-2.479136	4.980357	2.639454
132	1	0	-2.783556	0.932980	3.272098
133	1	0	-2.717781	0.766643	5.726039
134	1	0	-1.020867	2.027133	7.018692
135	1	0	0.609984	3.475244	5.839016
136	1	0	0.524297	3.696226	3.387134
137	1	0	-3.130118	-4.214516	-1.823882
138	1	0	-3.804182	-5.441596	-3.857660
139	1	0	-4.738615	-4.215283	-5.800166
140	1	0	-4.992507	-1.750328	-5.703661
141	1	0	-4.310502	-0.515756	-3.682890
142	1	0	-3.837342	3.180414	2.750318
143	1	0	-6.109480	3.309151	2.076939
144	1	0	-7.933422	3.060392	0.350238
145	1	0	-8.549344	2.578232	-1.931474
146	1	0	-6.876601	1.881548	-3.591161
147	1	0	-4.562335	1.620628	-3.008579
148	1	0	-2.756878	4.312332	-1.279945
149	1	0	-1.782771	5.941885	-2.738162
150	1	0	-0.292576	5.297345	-4.581180
151	1	0	0.262414	2.984486	-4.999001
152	1	0	-0.004256	0.505252	-4.405428
153	1	0	-1.078936	-1.231379	-3.146374
154	1	0	-7.711510	-0.230515	2.055230
155	1	0	-7.994014	-0.767896	-0.347974
156	1	0	-6.036463	-1.238532	-1.751875

157	1	0	-3.462303	-0.657819	1.656617
158	1	0	-5.442853	-0.175433	3.054048
159	6	0	-0.521047	-4.257644	2.610001
160	6	0	-0.034575	-5.422905	0.745080
161	6	0	0.869536	-5.907327	1.726537
162	8	0	0.503901	-5.099322	2.950808
163	6	0	-1.177688	-3.502116	3.663913
164	6	0	-2.462074	-2.914974	3.511872
165	6	0	-0.539374	-3.421595	4.932300
166	6	0	-3.069576	-2.240733	4.581700
167	1	0	-3.008631	-3.035138	2.579603
168	6	0	-1.155310	-2.749629	5.997959
169	1	0	0.426752	-3.898357	5.062080
170	6	0	-2.420436	-2.150387	5.830486
171	1	0	-4.064516	-1.819430	4.458703
172	1	0	-0.655409	-2.701949	6.961893
173	7	0	-0.826351	-4.405893	1.294882
174	8	0	1.779565	-6.750270	1.775734
175	1	0	-2.902268	-1.646225	6.664014
176	6	0	-0.246136	-6.055633	-0.594364
177	1	0	-1.117088	-5.619572	-1.097598
178	1	0	0.629823	-5.970332	-1.250865
179	1	0	-0.439165	-7.131084	-0.478793

[{(S_a)-Binap-Au}$_2$]-Ib

Center Number	Atomic Number	Atomic Type	Coordinates (Angstroms) X	Y	Z
1	6	0	1.197680	2.179353	-0.167897
2	6	0	-0.120371	1.580408	-0.526137
3	6	0	-0.299445	0.943568	-1.691461
4	6	0	0.809562	0.818182	-2.647104
5	6	0	0.600023	0.258111	-3.998892
6	6	0	1.635742	0.155516	-4.849320
7	6	0	2.932922	0.532955	-4.472829
8	6	0	3.189300	1.052642	-3.259581
9	6	0	2.083015	1.263380	-2.288272
10	6	0	2.271478	1.970424	-0.988021
11	6	0	3.629642	2.471216	-0.632097
12	6	0	4.383524	1.862277	0.325908
13	6	0	5.643736	2.380510	0.692003
14	6	0	6.153660	3.487264	0.130987
15	6	0	5.411182	4.179505	-0.934562
16	6	0	4.176585	3.669652	-1.337999
17	6	0	3.479809	4.349251	-2.462034
18	6	0	4.025414	5.436357	-3.036789
19	6	0	5.256548	5.954993	-2.606884
20	6	0	5.948965	5.381387	-1.607335
21	6	0	1.743197	6.586309	-0.674260
22	6	0	2.620609	7.326419	0.121157
23	6	0	3.119897	6.778152	1.304707
24	6	0	2.737153	5.492224	1.696796
25	6	0	1.854946	4.742269	0.907046
26	6	0	1.368527	5.294953	-0.291150
27	15	0	1.422999	3.040970	1.468270
28	6	0	-0.293348	3.367881	2.055591
29	6	0	-1.262177	4.036040	1.284759
30	6	0	-2.511509	4.338172	1.833406
31	6	0	-2.816223	3.954291	3.141007
32	6	0	-1.865245	3.278890	3.907822
33	6	0	-0.605234	2.999893	3.371602
34	15	0	3.835994	0.308865	1.141414
35	6	0	5.260266	-0.798894	0.830034
36	6	0	5.436264	-1.296582	-0.467671
37	6	0	6.478836	-2.183442	-0.749046
38	6	0	7.360377	-2.568935	0.263775
39	6	0	7.205726	-2.058661	1.555601
40	6	0	6.162219	-1.172194	1.840281
41	6	0	3.768511	0.584712	2.950535
42	6	0	3.919835	1.865508	3.507691
43	6	0	3.863355	2.043246	4.892895
44	6	0	3.665970	0.944671	5.732330

45	6	0	3.517022	-0.331860	5.186073
46	6	0	3.552450	-0.509574	3.799711
47	1	0	4.757831	-1.002368	-1.258595
48	1	0	6.601777	-2.572806	-1.751394
49	1	0	8.166874	-3.257066	0.046386
50	1	0	7.895928	-2.349261	2.336952
51	1	0	6.075491	-0.779231	2.842240
52	1	0	3.434266	-1.503439	3.388466
53	1	0	3.370222	-1.183732	5.837116
54	1	0	3.629529	1.082284	6.805178
55	1	0	3.976417	3.033204	5.315563
56	1	0	4.074328	2.731343	2.882196
57	1	0	-1.072064	4.315797	0.262964
58	1	0	-3.245697	4.869446	1.243484
59	1	0	-3.788640	4.180448	3.559026
60	1	0	-2.098441	2.984594	4.922825
61	1	0	0.135261	2.505610	3.988078
62	1	0	6.223899	1.880470	1.457620
63	1	0	7.123202	3.858316	0.449093
64	1	0	6.901738	5.800029	-1.297906
65	1	0	5.657729	6.834595	-3.094017
66	1	0	3.500770	5.927023	-3.846723
67	1	0	2.521218	3.988928	-2.819432
68	1	0	4.208454	1.325693	-3.006980
69	1	0	3.745096	0.401500	-5.176442
70	1	0	1.470393	-0.246009	-5.840809
71	1	0	-0.389990	-0.061480	-4.310194
72	1	0	-1.268487	0.534100	-1.940311
73	1	0	-0.955971	1.639738	0.155813
74	1	0	2.919486	8.320931	-0.183460
75	1	0	1.371126	7.003910	-1.600822
76	1	0	0.734032	4.724166	-0.953220
77	1	0	3.131295	5.079314	2.616548
78	1	0	3.804122	7.349301	1.918585
79	79	0	-3.350691	1.119955	-0.857272
80	79	0	1.877446	-0.899814	0.164169
81	6	0	-1.830040	-1.473859	1.060244
82	6	0	-1.568778	-0.417202	2.071388
83	6	0	-0.731022	-0.615749	3.096147
84	6	0	-0.034681	-1.901441	3.251262
85	6	0	0.749659	-2.192533	4.470209
86	6	0	1.344355	-3.387701	4.620268
87	6	0	1.224419	-4.389794	3.648068
88	6	0	0.507899	-4.198939	2.526579
89	6	0	-0.164136	-2.893852	2.274427
90	6	0	-1.046422	-2.660425	1.094803
91	6	0	-1.203569	-3.765728	0.123021
92	6	0	-0.278421	-4.009005	-0.849261
93	6	0	-0.491752	-5.173681	-1.761118
94	6	0	-1.523099	-6.013678	-1.598780
95	6	0	-2.422134	-5.849440	-0.545309
96	6	0	-2.281281	-4.776781	0.347366
97	6	0	-3.204311	-4.719925	1.510239
98	6	0	-4.187865	-5.626392	1.642288
99	6	0	-4.359773	-6.662222	0.713155
100	6	0	-3.534298	-6.806312	-0.337628
101	6	0	-4.770495	-4.207855	-2.359419
102	6	0	-3.972744	-4.135510	-3.502365
103	6	0	-2.953107	-3.185297	-3.580846
104	6	0	-2.719777	-2.314887	-2.510590
105	6	0	-3.510235	-2.383867	-1.355645
106	6	0	-4.548454	-3.331632	-1.293402
107	15	0	-3.279606	-1.130213	-0.038358
108	6	0	-4.770416	-1.245907	1.024823
109	6	0	-6.014127	-0.912403	0.466760
110	6	0	-7.175675	-0.965663	1.242057
111	6	0	-7.102304	-1.338829	2.585798
112	6	0	-5.866261	-1.652803	3.155921
113	6	0	-4.702422	-1.600820	2.382752
114	15	0	1.317305	-3.068836	-0.983241
115	6	0	2.519234	-4.222660	-0.220758
116	6	0	3.266835	-3.785190	0.881069
117	6	0	4.217062	-4.623067	1.469434
118	6	0	4.430154	-5.904697	0.956674
119	6	0	3.695118	-6.346091	-0.147215
120	6	0	2.744616	-5.507679	-0.738392
121	6	0	1.846847	-3.066894	-2.740112

122	6	0	3.213044	-3.067588	-3.062202
123	6	0	3.624484	-3.092249	-4.397866
124	6	0	2.674463	-3.115425	-5.421869
125	6	0	1.312790	-3.095153	-5.109698
126	6	0	0.902220	-3.050092	-3.774366
127	1	0	3.106958	-2.799064	1.292234
128	1	0	4.787325	-4.277869	2.322205
129	1	0	5.167330	-6.553658	1.411221
130	1	0	3.868162	-7.335375	-0.550574
131	1	0	2.210840	-5.856156	-1.608774
132	1	0	-0.149136	-3.009353	-3.548017
133	1	0	0.575527	-3.095491	-5.901952
134	1	0	2.992812	-3.137729	-6.455910
135	1	0	4.679659	-3.099108	-4.638313
136	1	0	3.960662	-3.067857	-2.282270
137	1	0	-6.082966	-0.612578	-0.571445
138	1	0	-8.131233	-0.710190	0.802901
139	1	0	-8.001219	-1.375579	3.187289
140	1	0	-5.808715	-1.930998	4.200262
141	1	0	-3.761619	-1.832206	2.856490
142	1	0	0.193826	-5.360752	-2.578131
143	1	0	-1.641138	-6.843404	-2.285201
144	1	0	-3.687466	-7.626781	-1.032585
145	1	0	-5.167428	-7.369110	0.854473
146	1	0	-4.857797	-5.561708	2.490322
147	1	0	-3.083832	-3.960182	2.270573
148	1	0	0.430841	-5.011058	1.811426
149	1	0	1.713748	-5.342061	3.809015
150	1	0	1.921397	-3.585267	5.514628
151	1	0	0.839886	-1.442405	5.249535
152	1	0	-0.599297	0.160047	3.842080
153	1	0	-2.108024	0.523808	2.013140
154	1	0	-4.147262	-4.812414	-4.328465
155	1	0	-5.560917	-4.944551	-2.297660
156	1	0	-5.178229	-3.415205	-0.419879
157	1	0	-1.931613	-1.580984	-2.589133
158	1	0	-2.348744	-3.119715	-4.475892
159	6	0	-4.798195	3.741246	-0.851262
160	6	0	-3.138016	3.823956	-2.370228
161	6	0	-3.835744	5.050520	-2.521720
162	8	0	-4.940506	4.941092	-1.487225
163	6	0	-5.726961	3.386987	0.215830
164	6	0	-5.387355	2.512032	1.281431
165	6	0	-7.013267	3.988478	0.217636
166	6	0	-6.314108	2.221951	2.294165
167	1	0	-4.381396	2.105229	1.353765
168	6	0	-7.937765	3.688842	1.229175
169	1	0	-7.268758	4.683243	-0.576317
170	6	0	-7.598827	2.799374	2.269292
171	1	0	-6.028516	1.569100	3.115993
172	1	0	-8.920512	4.152366	1.210738
173	7	0	-3.744439	3.060330	-1.365721
174	8	0	-3.743470	6.059428	-3.238638
175	1	0	-8.315216	2.576659	3.055437
176	6	0	-1.964757	3.409209	-3.195573
177	1	0	-1.011854	3.476640	-2.646434
178	1	0	-2.068411	2.385098	-3.584357
179	1	0	-1.886045	4.085125	-4.053603

***tert*-Bu acrylate**

Center Number	Atomic Number	Atomic Type	Coordinates (Angstroms) X	Y	Z
1	6	0	-1.000082	-0.447706	0.000000
2	8	0	-0.162097	0.647696	0.000000
3	8	0	-0.620101	-1.636366	0.000000
4	6	0	1.348800	0.519533	0.000000
5	6	0	-2.419144	0.003075	0.000000
6	6	0	-3.436255	-0.884152	0.000000
7	1	0	-2.579986	1.077262	0.000000
8	1	0	-4.473868	-0.560259	0.000000

9	1	0	-3.235092	-1.953264	0.000000
10	6	0	1.797246	1.988815	0.000000
11	6	0	1.797246	-0.208578	1.281698
12	6	0	1.797246	-0.208578	-1.281698
13	1	0	2.893350	-0.200143	-1.338776
14	1	0	1.454926	-1.246416	-1.287817
15	1	0	1.404561	0.301958	-2.169405
16	1	0	2.892805	2.046737	0.000000
17	1	0	1.418856	2.506822	-0.888397
18	1	0	1.418856	2.506822	0.888397
19	1	0	2.893350	-0.200143	1.338776
20	1	0	1.404561	0.301958	2.169405
21	1	0	1.454926	-1.246416	1.287817

TS10

Center Number	Atomic Number	Atomic Type	Coordinates (Angstroms) X	Y	Z
1	7	0	3.803035	2.086858	0.512863
2	6	0	4.211051	2.695442	1.702129
3	8	0	4.511957	4.050431	1.391165
4	6	0	4.662432	4.142088	-0.041771
5	6	0	4.249945	2.834134	-0.532104
6	6	0	3.567112	2.468581	3.017810
7	6	0	3.543926	1.181440	3.606939
8	6	0	2.961212	0.985000	4.869523
9	6	0	2.391405	2.069984	5.564976
10	6	0	2.419063	3.354712	4.987717
11	6	0	3.008601	3.557105	3.727661
12	8	0	5.013062	5.183904	-0.614747
13	79	0	3.182380	0.138677	0.327116
14	15	0	2.408318	-2.097286	0.114333
15	6	0	0.821661	-2.217441	-0.819036
16	6	0	0.635040	-1.233028	-1.908725
17	6	0	-0.160740	-1.494964	-2.957849
18	6	0	-0.885334	-2.778018	-3.075020
19	6	0	-0.839870	-3.719844	-2.042513
20	6	0	-0.068489	-3.419056	-0.813011
21	6	0	-1.562840	-5.004345	-2.233367
22	6	0	-2.248863	-5.231619	-3.366725
23	6	0	-2.302688	-4.278659	-4.394093
24	6	0	-1.664442	-3.100034	-4.292243
25	6	0	-0.286853	-4.235704	0.401583
26	6	0	-1.285325	-3.955731	1.285084
27	6	0	-1.437214	-4.833632	2.486885
28	6	0	-0.624515	-5.877848	2.701992
29	6	0	0.366970	-6.217131	1.781568
30	6	0	0.538057	-5.461427	0.613229
31	6	0	1.525832	-5.944408	-0.384418
32	6	0	2.287451	-7.019957	-0.120991
33	6	0	2.155600	-7.735215	1.077835
34	6	0	1.244976	-7.389852	2.004274
35	15	0	-2.601064	-2.691493	0.941242
36	6	0	-3.556079	-2.453150	2.489057
37	6	0	-4.960984	-2.502117	2.496953
38	6	0	-5.667182	-2.291877	3.684902
39	6	0	-4.979184	-2.025917	4.870998
40	6	0	-3.584593	-1.954276	4.866479
41	6	0	-2.877662	-2.143313	3.675333
42	6	0	2.146634	-2.844055	1.765483
43	6	0	1.264460	-2.209795	2.649048
44	6	0	1.097815	-2.689738	3.951395
45	6	0	1.804971	-3.815770	4.376875
46	6	0	2.679738	-4.461543	3.500497
47	6	0	2.853026	-3.979207	2.199486
48	79	0	-2.409939	-0.468258	-0.150938
49	15	0	-3.820620	1.181184	-1.338965
50	6	0	-3.318173	1.549529	-3.057534
51	6	0	-3.318178	2.859826	-3.562472
52	6	0	-2.877653	3.111704	-4.865075
53	6	0	-2.447027	2.058180	-5.674826
54	6	0	-2.463811	0.750245	-5.185026

55	6	0	-2.899317	0.496360	-3.880885
56	6	0	-4.215561	2.763448	-0.488356
57	6	0	-3.381250	3.287863	0.449652
58	6	0	-3.734897	4.574732	1.114214
59	6	0	-4.918577	5.213978	0.746695
60	6	0	-5.775908	4.590310	-0.276126
61	6	0	-5.410171	3.427978	-0.840660
62	6	0	-2.872400	5.232462	2.130328
63	6	0	-3.239933	6.411226	2.664373
64	6	0	-4.434112	7.047356	2.289427
65	6	0	-5.262527	6.506165	1.378281
66	6	0	-2.088914	2.641592	0.773171
67	6	0	-0.959353	2.854721	-0.042254
68	6	0	0.314191	2.170478	0.301648
69	6	0	0.439258	1.486546	1.450685
70	6	0	-0.690365	1.382525	2.394324
71	6	0	-1.940201	1.899649	2.047312
72	6	0	-3.044120	1.783700	3.036123
73	6	0	-2.810570	1.253022	4.248540
74	6	0	-1.544587	0.764446	4.602672
75	6	0	-0.516771	0.790895	3.738552
76	15	0	-1.064547	4.141965	-1.378686
77	6	0	0.263998	3.636534	-2.549178
78	6	0	1.073325	4.608912	-3.161240
79	6	0	1.938785	4.256738	-4.200483
80	6	0	1.987369	2.936862	-4.653285
81	6	0	1.166779	1.969514	-4.070010
82	6	0	0.299282	2.319410	-3.029697
83	6	0	-0.218444	5.483383	-0.443523
84	6	0	-0.932841	6.659952	-0.180948
85	6	0	-0.352228	7.687144	0.568396
86	6	0	0.950808	7.548947	1.053094
87	6	0	1.675924	6.385485	0.784141
88	6	0	1.096659	5.355522	0.037353
89	6	0	-5.435011	0.328291	-1.441947
90	6	0	-5.991823	-0.183678	-0.261665
91	6	0	-7.195599	-0.892479	-0.298908
92	6	0	-7.852648	-1.090496	-1.515772
93	6	0	-7.308960	-0.574396	-2.695278
94	6	0	-6.103859	0.134302	-2.661863
95	6	0	-3.752687	-3.614283	-0.135495
96	6	0	-4.184863	-3.030996	-1.333733
97	6	0	-5.087964	-3.705250	-2.159431
98	6	0	-5.554396	-4.971742	-1.796965
99	6	0	-5.119732	-5.562727	-0.606603
100	6	0	-4.222378	-4.885939	0.225300
101	1	0	-5.484335	-0.045881	0.685215
102	1	0	-7.615964	-1.292492	0.614709
103	1	0	-8.783119	-1.642388	-1.544754
104	1	0	-7.820399	-0.726121	-3.636941
105	1	0	-5.705989	0.525411	-3.586831
106	1	0	-2.909015	-0.519319	-3.509264
107	1	0	-2.135313	-0.064814	-5.815522
108	1	0	-2.103619	2.254812	-6.682092
109	1	0	-2.866681	4.124901	-5.245313
110	1	0	-3.641882	3.691994	-2.955214
111	1	0	1.012090	5.646864	-2.862889
112	1	0	2.561808	5.010359	-4.664307
113	1	0	2.655486	2.665239	-5.460136
114	1	0	1.198011	0.953033	-4.435601
115	1	0	-0.363672	1.572279	-2.615347
116	1	0	-6.055664	3.003693	-1.598885
117	1	0	-6.699873	5.072888	-0.580005
118	1	0	-6.178788	7.020618	1.105103
119	1	0	-4.691775	7.995619	2.743758
120	1	0	-2.598679	6.883443	3.397794
121	1	0	-1.936142	4.779389	2.439675
122	1	0	-4.040106	2.143391	2.798956
123	1	0	-3.619141	1.191948	4.965872
124	1	0	-1.398346	0.338868	5.587229
125	1	0	0.442608	0.388796	4.036580
126	1	0	1.381587	1.017339	1.699075
127	1	0	1.164960	2.229785	-0.365240
128	1	0	1.399576	8.344270	1.633905
129	1	0	2.687766	6.282963	1.153499
130	1	0	1.681783	4.474610	-0.173580
131	1	0	-1.947878	6.773346	-0.541020

132	1	0	-0.914671	8.587897	0.777329
133	1	0	-3.817914	-2.059306	-1.632308
134	1	0	-5.419160	-3.249142	-3.083369
135	1	0	-6.250122	-5.495883	-2.439076
136	1	0	-5.481915	-6.543249	-0.326342
137	1	0	-3.904889	-5.347355	1.150484
138	1	0	-1.799898	-2.065882	3.678574
139	1	0	-3.051612	-1.738009	5.783109
140	1	0	-5.526575	-1.867046	5.790974
141	1	0	-6.748726	-2.333284	3.684966
142	1	0	-5.518035	-2.690461	1.590489
143	1	0	-2.232313	-4.659853	3.199725
144	1	0	-0.765723	-6.482360	3.589835
145	1	0	1.158043	-7.973452	2.916111
146	1	0	2.790450	-8.594627	1.252054
147	1	0	3.010421	-7.350271	-0.856131
148	1	0	1.623497	-5.451207	-1.341827
149	1	0	-1.549078	-5.766266	-1.460840
150	1	0	-2.776900	-6.168868	-3.488139
151	1	0	-2.869310	-4.499798	-5.289599
152	1	0	-1.718165	-2.388277	-5.109071
153	1	0	-0.244328	-0.768619	-3.756928
154	1	0	1.199385	-0.306115	-1.887447
155	1	0	1.671201	-4.190952	5.383045
156	1	0	3.218193	-5.342059	3.826165
157	1	0	3.516382	-4.514444	1.538943
158	1	0	0.704258	-1.346008	2.323065
159	1	0	0.423248	-2.188619	4.632581
160	1	0	4.005434	0.340205	3.094378
161	1	0	3.051496	4.548938	3.289104
162	1	0	2.966346	-0.006154	5.317924
163	1	0	1.993812	4.199893	5.523504
164	1	0	1.949514	1.920264	6.547001
165	6	0	6.725956	2.021819	0.540456
166	1	0	7.300059	2.857017	0.156409
167	6	0	6.197066	2.007101	1.839837
168	1	0	6.496586	2.785838	2.537231
169	6	0	3.649360	-3.087363	-0.801887
170	6	0	4.935657	-3.245346	-0.263393
171	6	0	3.380945	-3.588017	-2.086903
172	6	0	5.914459	-3.955043	-0.964789
173	1	0	5.178649	-2.823486	0.703617
174	6	0	4.364056	-4.291044	-2.789620
175	1	0	2.418834	-3.438375	-2.554807
176	6	0	5.628176	-4.479945	-2.226615
177	1	0	6.898492	-4.088349	-0.534674
178	1	0	4.147339	-4.683701	-3.774572
179	1	0	6.388952	-5.022297	-2.772719
180	6	0	4.146732	2.480962	-1.977598
181	1	0	5.041186	1.946521	-2.321705
182	1	0	3.273632	1.844622	-2.170312
183	1	0	4.072860	3.398350	-2.569589
184	6	0	6.600315	0.847795	-0.296858
185	8	0	7.265314	0.980484	-1.509033
186	8	0	5.935753	-0.199016	0.002937
187	1	0	5.958565	1.042962	2.282009
188	6	0	7.561660	-0.193509	-2.414475
189	6	0	8.347081	-1.254519	-1.619492
190	6	0	6.252826	-0.755430	-3.007365
191	6	0	8.432541	0.445814	-3.506946
192	1	0	9.271943	-0.822123	-1.220517
193	1	0	7.753641	-1.641823	-0.787458
194	1	0	8.615715	-2.088062	-2.280828
195	1	0	5.724711	0.018830	-3.577122
196	1	0	6.488051	-1.580955	-3.691818
197	1	0	5.599388	-1.122922	-2.212945
198	1	0	8.721907	-0.309057	-4.248890
199	1	0	7.886276	1.247716	-4.016380
200	1	0	9.340412	0.875545	-3.070671

TS10ent

Center   Atomic   Atomic   Coordinates (Angstroms)

Number	Number	Type	X	Y	Z
1	7	0	4.019208	2.468674	0.371615
2	6	0	5.221233	2.884532	-0.228390
3	8	0	5.625046	4.075910	0.437373
4	6	0	4.875084	4.174099	1.659240
5	6	0	3.897555	3.095324	1.570620
6	6	0	5.540996	2.858566	-1.678416
7	6	0	5.084358	1.840418	-2.546947
8	6	0	5.471425	1.812369	-3.895841
9	6	0	6.330046	2.803804	-4.407989
10	6	0	6.789893	3.823158	-3.552088
11	6	0	6.405445	3.851402	-2.200974
12	8	0	5.072917	5.057117	2.505702
13	79	0	3.180615	0.624109	-0.047048
14	15	0	2.598255	-1.547001	-0.841599
15	6	0	3.817267	-1.845047	-2.180864
16	6	0	5.181757	-1.712741	-1.879732
17	6	0	6.146777	-1.871900	-2.877518
18	6	0	5.756465	-2.156005	-4.187797
19	6	0	4.400496	-2.275385	-4.501224
20	6	0	3.432322	-2.113287	-3.505620
21	6	0	0.929556	-1.661196	-1.635331
22	6	0	0.613932	-0.539366	-2.556406
23	6	0	-0.405676	-0.606277	-3.421268
24	6	0	-1.253323	-1.807075	-3.480883
25	6	0	-1.077786	-2.838532	-2.552857
26	6	0	0.017388	-2.747215	-1.545679
27	6	0	-1.932500	-4.049301	-2.698721
28	6	0	-2.840954	-4.125224	-3.686872
29	6	0	-2.997472	-3.090600	-4.619140
30	6	0	-2.250119	-1.975826	-4.559381
31	6	0	0.206498	-3.888740	-0.624385
32	6	0	-0.548054	-4.037928	0.501226
33	6	0	-0.317842	-5.239747	1.359410
34	6	0	0.559007	-6.192538	1.014175
35	6	0	1.267946	-6.113809	-0.184314
36	6	0	1.097301	-5.014872	-1.038886
37	6	0	1.797442	-5.042748	-2.348795
38	6	0	2.628041	-6.054377	-2.654062
39	6	0	2.839985	-7.121279	-1.769367
40	6	0	2.204195	-7.189321	-0.587185
41	15	0	-1.966825	-2.919139	0.922731
42	6	0	-2.178164	-2.898123	2.744126
43	6	0	-3.457942	-2.752647	3.302492
44	6	0	-3.629606	-2.767332	4.689746
45	6	0	-2.524548	-2.926587	5.529584
46	6	0	-1.245298	-3.051250	4.981875
47	6	0	-1.072124	-3.015319	3.595471
48	6	0	2.899851	-2.851093	0.412296
49	6	0	2.316869	-2.726163	1.679854
50	6	0	2.628251	-3.636170	2.696060
51	6	0	3.517001	-4.683662	2.450795
52	6	0	4.102792	-4.818525	1.191487
53	6	0	3.802517	-3.904876	0.177282
54	79	0	-2.457380	-0.701774	-0.064984
55	15	0	-4.271641	0.831221	-0.716716
56	6	0	-4.460957	1.136513	-2.511429
57	6	0	-4.493497	2.438837	-3.035913
58	6	0	-4.655647	2.642935	-4.409023
59	6	0	-4.800481	1.550277	-5.266672
60	6	0	-4.775035	0.252366	-4.751920
61	6	0	-4.588709	0.044952	-3.381694
62	6	0	-4.444685	2.422318	0.184904
63	6	0	-3.474897	2.882294	1.024454
64	6	0	-3.734198	4.114879	1.830896
65	6	0	-4.926212	4.810933	1.626920
66	6	0	-5.907674	4.276686	0.670006
67	6	0	-5.653983	3.130850	0.020694
68	6	0	-2.792533	4.640197	2.855474
69	6	0	-3.087173	5.765877	3.530675
70	6	0	-4.278483	6.470603	3.299823
71	6	0	-5.181931	6.045302	2.399744
72	6	0	-2.161979	2.188149	1.156633
73	6	0	-1.202130	2.284905	0.187375
74	6	0	0.073410	1.528567	0.345939
75	6	0	0.348766	0.858211	1.472715

76	6	0	-0.622578	0.833085	2.576035
77	6	0	-1.874233	1.428630	2.408013
78	6	0	-2.849582	1.319327	3.525691
79	6	0	-2.487179	0.741880	4.684325
80	6	0	-1.204198	0.206082	4.865711
81	6	0	-0.295448	0.210710	3.875739
82	15	0	-1.555455	3.191700	-1.401338
83	6	0	0.095058	3.370830	-2.201435
84	6	0	1.217442	3.903882	-1.541737
85	6	0	2.403396	4.132666	-2.243490
86	6	0	2.490577	3.809103	-3.598998
87	6	0	1.389477	3.256675	-4.255648
88	6	0	0.193472	3.050557	-3.562546
89	6	0	-1.736095	4.920894	-0.787237
90	6	0	-2.620198	5.772583	-1.464051
91	6	0	-2.808962	7.087768	-1.029984
92	6	0	-2.109788	7.565126	0.080677
93	6	0	-1.224691	6.725887	0.760481
94	6	0	-1.043414	5.405941	0.336430
95	6	0	-5.778618	-0.059241	-0.180562
96	6	0	-5.798945	-0.600000	1.111732
97	6	0	-6.905029	-1.329799	1.555097
98	6	0	-8.004344	-1.512712	0.712673
99	6	0	-8.001848	-0.956240	-0.569349
100	6	0	-6.895440	-0.226865	-1.016185
101	6	0	-3.414982	-3.897832	0.375401
102	6	0	-4.284742	-3.343427	-0.573821
103	6	0	-5.417095	-4.046370	-0.991948
104	6	0	-5.690196	-5.309085	-0.460871
105	6	0	-4.832473	-5.866233	0.491801
106	6	0	-3.699617	-5.162710	0.913152
107	1	0	-4.951240	-0.462468	1.771114
108	1	0	-6.908836	-1.754508	2.550608
109	1	0	-8.860530	-2.079024	1.055446
110	1	0	-8.858916	-1.088525	-1.216881
111	1	0	-6.926339	0.208644	-2.003931
112	1	0	-4.570468	-0.965587	-2.995041
113	1	0	-4.895728	-0.593865	-5.415627
114	1	0	-4.935150	1.709203	-6.328698
115	1	0	-4.675944	3.649261	-4.806973
116	1	0	-4.394019	3.299628	-2.393307
117	1	0	1.192425	4.138415	-0.491424
118	1	0	3.253467	4.567733	-1.735727
119	1	0	3.409565	3.989951	-4.140722
120	1	0	1.455571	3.007618	-5.306754
121	1	0	-0.665938	2.660994	-4.093320
122	1	0	-6.408098	2.749266	-0.656356
123	1	0	-6.847833	4.794835	0.507149
124	1	0	-6.098058	6.606863	2.244349
125	1	0	-4.474067	7.375061	3.861662
126	1	0	-2.386497	6.141755	4.265371
127	1	0	-1.853934	4.135893	3.057439
128	1	0	-3.855219	1.713791	3.424569
129	1	0	-3.201541	0.685467	5.495807
130	1	0	-0.949540	-0.242147	5.817520
131	1	0	0.677751	-0.238635	4.034929
132	1	0	1.292886	0.338645	1.573245
133	1	0	0.798759	1.501304	-0.454257
134	1	0	-2.258043	8.582720	0.417887
135	1	0	-0.695233	7.090847	1.631023
136	1	0	-0.395005	4.763534	0.913880
137	1	0	-3.163781	5.418597	-2.330204
138	1	0	-3.496583	7.737207	-1.555951
139	1	0	-4.081782	-2.371320	-0.999042
140	1	0	-6.081655	-3.611478	-1.727376
141	1	0	-6.567974	-5.853594	-0.783747
142	1	0	-5.050149	-6.840266	0.910410
143	1	0	-3.067357	-5.597357	1.671771
144	1	0	-0.079464	-3.087076	3.187181
145	1	0	-0.385772	-3.156065	5.631166
146	1	0	-2.657731	-2.941336	6.603430
147	1	0	-4.620032	-2.661285	5.112921
148	1	0	-4.325577	-2.646635	2.667139
149	1	0	5.496685	-1.484067	-0.869646
150	1	0	7.196633	-1.769645	-2.635509
151	1	0	6.503748	-2.276574	-4.961266
152	1	0	4.098348	-2.485210	-5.519099

153	1	0	2.393412	-2.189019	-3.785089
154	1	0	-0.864343	-5.362060	2.286248
155	1	0	0.697556	-7.044572	1.668682
156	1	0	2.381310	-8.035527	0.070198
157	1	0	3.520443	-7.914245	-2.052476
158	1	0	3.138411	-6.051286	-3.608878
159	1	0	1.632393	-4.259695	-3.076353
160	1	0	-1.835709	-4.883629	-2.012010
161	1	0	-3.459298	-5.009865	-3.771857
162	1	0	-3.729894	-3.197223	-5.409136
163	1	0	-2.373660	-1.201434	-5.309713
164	1	0	-0.581052	0.211637	-4.111699
165	1	0	1.256827	0.335677	-2.572700
166	1	0	3.754828	-5.387600	3.237468
167	1	0	4.789573	-5.632559	0.999426
168	1	0	4.265385	-4.041653	-0.788491
169	1	0	1.631543	-1.917872	1.885890
170	1	0	2.191902	-3.523539	3.679488
171	1	0	4.406358	1.077290	-2.177055
172	1	0	6.758118	4.640559	-1.545793
173	1	0	5.104269	1.021358	-4.546461
174	1	0	7.449205	4.597591	-3.935981
175	1	0	6.633870	2.783389	-5.451350
176	6	0	6.574786	1.582532	0.623813
177	1	0	7.431242	2.163323	0.288088
178	6	0	6.329332	1.504222	2.006434
179	1	0	6.833412	2.157673	2.709256
180	6	0	2.824711	2.882681	2.587527
181	1	0	1.842824	3.210097	2.214038
182	1	0	2.752330	1.831532	2.891347
183	1	0	3.058335	3.481396	3.473282
184	1	0	6.337579	0.717373	0.006517
185	6	0	5.504046	0.457720	2.557586
186	8	0	5.570169	0.416350	3.942892
187	8	0	4.786237	-0.363443	1.885026
188	6	0	4.975230	-0.693156	4.768390
189	6	0	3.438526	-0.627348	4.676745
190	6	0	5.537362	-2.051274	4.302882
191	6	0	5.460456	-0.347470	6.184975
192	1	0	3.080124	0.348040	5.027386
193	1	0	3.116622	-0.775750	3.643044
194	1	0	2.993463	-1.405606	5.310754
195	1	0	6.632461	-2.044207	4.351595
196	1	0	5.171029	-2.846692	4.964868
197	1	0	5.235117	-2.272186	3.276274
198	1	0	5.073424	-1.077384	6.907391
199	1	0	6.554748	-0.357633	6.228011
200	1	0	5.117532	0.651842	6.474644

TS10exo

Center Number	Atomic Number	Atomic Type	Coordinates (Angstroms) X	Y	Z
1	7	0	4.061575	-1.173596	0.503602
2	6	0	4.620050	-0.570950	1.638480
3	8	0	5.304073	0.590748	1.187754
4	6	0	5.522868	0.435710	-0.235108
5	6	0	4.808579	-0.780241	-0.580274
6	6	0	3.932022	-0.442763	2.942020
7	6	0	3.371534	-1.576353	3.579992
8	6	0	2.766852	-1.458193	4.842338
9	6	0	2.718012	-0.208120	5.491867
10	6	0	3.281336	0.921146	4.865247
11	6	0	3.889784	0.806633	3.603366
12	8	0	6.144892	1.270390	-0.910615
13	79	0	2.380306	-2.293801	0.334274
14	15	0	0.354070	-3.497634	-0.040240
15	6	0	-0.880509	-2.503661	-0.985712
16	6	0	-0.323995	-1.577186	-1.998664
17	6	0	-1.040740	-1.198645	-3.069045
18	6	0	-2.418831	-1.686899	-3.285777
19	6	0	-3.051134	-2.488559	-2.330011

20	6	0	-2.338346	-2.832209	-1.076162
21	6	0	-4.420539	-2.983872	-2.626970
22	6	0	-5.027630	-2.643773	-3.777006
23	6	0	-4.397120	-1.823559	-4.723714
24	6	0	-3.154599	-1.350400	-4.525766
25	6	0	-3.102119	-3.376983	0.070079
26	6	0	-3.738238	-2.566024	0.961671
27	6	0	-4.468055	-3.199472	2.103903
28	6	0	-4.519096	-4.530438	2.254033
29	6	0	-3.933882	-5.380997	1.316602
30	6	0	-3.266856	-4.855036	0.201237
31	6	0	-2.787766	-5.809366	-0.829778
32	6	0	-2.907161	-7.134251	-0.638180
33	6	0	-3.512696	-7.656178	0.513683
34	6	0	-4.023719	-6.852934	1.462466
35	15	0	-3.935087	-0.738955	0.694552
36	6	0	-4.598714	-0.035048	2.252858
37	6	0	-5.746737	0.776366	2.264485
38	6	0	-6.210873	1.323602	3.464177
39	6	0	-5.532405	1.070306	4.658512
40	6	0	-4.380433	0.281074	4.651644
41	6	0	-3.903308	-0.250873	3.450197
42	6	0	-0.414809	-4.009075	1.539185
43	6	0	-0.775639	-3.014497	2.456624
44	6	0	-1.293255	-3.358876	3.708553
45	6	0	-1.459753	-4.702637	4.049827
46	6	0	-1.109686	-5.701998	3.138772
47	6	0	-0.588514	-5.359608	1.887026
48	79	0	-2.325208	0.933969	-0.196120
49	15	0	-2.369400	3.202044	-1.182582
50	6	0	-1.712013	3.313236	-2.883779
51	6	0	-1.004175	4.439095	-3.334547
52	6	0	-0.478770	4.466281	-4.629845
53	6	0	-0.665846	3.377473	-5.484956
54	6	0	-1.385580	2.262913	-5.048159
55	6	0	-1.910613	2.232547	-3.752974
56	6	0	-1.701432	4.592230	-0.179990
57	6	0	-0.752177	4.381417	0.773076
58	6	0	-0.213967	5.537840	1.545098
59	6	0	-0.712669	6.812034	1.275557
60	6	0	-1.756798	6.964858	0.247555
61	6	0	-2.199933	5.889835	-0.424256
62	6	0	0.868002	5.411013	2.556707
63	6	0	1.337129	6.505795	3.181934
64	6	0	0.826335	7.784393	2.907196
65	6	0	-0.153756	7.970667	2.004972
66	6	0	-0.179244	3.033916	1.001799
67	6	0	0.792543	2.513339	0.124229
68	6	0	1.328234	1.147627	0.368620
69	6	0	0.971649	0.455195	1.462757
70	6	0	0.052647	1.035763	2.459967
71	6	0	-0.540471	2.277819	2.224446
72	6	0	-1.441887	2.831000	3.268956
73	6	0	-1.617762	2.171290	4.426695
74	6	0	-0.994359	0.938779	4.669740
75	6	0	-0.207455	0.358328	3.748921
76	15	0	1.442915	3.613495	-1.224675
77	6	0	2.381845	2.452015	-2.300530
78	6	0	3.716704	2.701125	-2.666526
79	6	0	4.340140	1.913231	-3.638684
80	6	0	3.629253	0.894323	-4.276147
81	6	0	2.297340	0.653749	-3.933274
82	6	0	1.675254	1.433895	-2.954678
83	6	0	2.784625	4.388776	-0.238205
84	6	0	2.863929	5.786888	-0.195973
85	6	0	3.842839	6.413999	0.580458
86	6	0	4.749920	5.646724	1.316457
87	6	0	4.679153	4.251664	1.275791
88	6	0	3.697688	3.623087	0.504488
89	6	0	-4.159494	3.555734	-1.295864
90	6	0	-4.934519	3.449733	-0.132632
91	6	0	-6.316015	3.654138	-0.184478
92	6	0	-6.931352	3.966366	-1.399240
93	6	0	-6.164357	4.079691	-2.561985
94	6	0	-4.781777	3.875562	-2.513884
95	6	0	-5.349610	-0.655325	-0.458142
96	6	0	-5.257678	0.157601	-1.595443

97	6	0	-6.337397	0.260766	-2.476133
98	6	0	-7.510647	-0.457501	-2.230096
99	6	0	-7.605098	-1.277050	-1.101138
100	6	0	-6.528594	-1.375414	-0.214145
101	1	0	-4.469058	3.195389	0.811724
102	1	0	-6.909956	3.564653	0.715904
103	1	0	-8.001790	4.120377	-1.439789
104	1	0	-6.642163	4.321509	-3.502476
105	1	0	-4.211663	3.965254	-3.427158
106	1	0	-2.465680	1.365146	-3.422594
107	1	0	-1.532129	1.422015	-5.712450
108	1	0	-0.255436	3.398485	-6.486115
109	1	0	0.077924	5.330189	-4.969008
110	1	0	-0.836292	5.288047	-2.689071
111	1	0	4.270890	3.523545	-2.237664
112	1	0	5.369207	2.106458	-3.912791
113	1	0	4.110599	0.290955	-5.034734
114	1	0	1.746325	-0.130936	-4.433835
115	1	0	0.633983	1.264443	-2.719490
116	1	0	-2.954332	6.038802	-1.186208
117	1	0	-2.160111	7.947656	0.023174
118	1	0	-0.527309	8.971068	1.808821
119	1	0	1.233613	8.639059	3.432169
120	1	0	2.131054	6.400449	3.910389
121	1	0	1.299878	4.442880	2.788530
122	1	0	-1.950108	3.777606	3.117197
123	1	0	-2.262833	2.594766	5.186009
124	1	0	-1.166151	0.436181	5.612994
125	1	0	0.248678	-0.600819	3.962530
126	1	0	1.382974	-0.530058	1.635165
127	1	0	2.038355	0.702296	-0.316815
128	1	0	5.506784	6.132892	1.918097
129	1	0	5.384100	3.657771	1.842740
130	1	0	3.654304	2.545748	0.483256
131	1	0	2.157253	6.390308	-0.751883
132	1	0	3.893941	7.494422	0.616467
133	1	0	-4.349988	0.705924	-1.803673
134	1	0	-6.261089	0.891170	-3.352546
135	1	0	-8.345029	-0.381065	-2.914945
136	1	0	-8.514279	-1.832381	-0.911035
137	1	0	-6.619203	-2.001309	0.663172
138	1	0	-3.001816	-0.847345	3.452993
139	1	0	-3.850058	0.091171	5.575609
140	1	0	-5.894767	1.491361	5.587297
141	1	0	-7.095775	1.946821	3.466893
142	1	0	-6.280778	1.002992	1.353234
143	1	0	-4.996815	-2.591480	2.826001
144	1	0	-5.052341	-4.946022	3.100553
145	1	0	-4.501481	-7.288446	2.335177
146	1	0	-3.586607	-8.729833	0.630705
147	1	0	-2.541698	-7.814562	-1.397016
148	1	0	-2.367905	-5.447165	-1.757602
149	1	0	-4.944177	-3.617954	-1.918941
150	1	0	-6.028565	-3.005485	-3.975419
151	1	0	-4.923048	-1.569162	-5.635063
152	1	0	-2.691041	-0.725603	-5.281904
153	1	0	-0.587095	-0.554208	-3.813444
154	1	0	0.705022	-1.244183	-1.904651
155	1	0	-1.865860	-4.969317	5.016772
156	1	0	-1.250290	-6.743115	3.398772
157	1	0	-0.347308	-6.152277	1.196323
158	1	0	-0.664577	-1.972921	2.192926
159	1	0	-1.565468	-2.585526	4.414421
160	1	0	3.441797	-2.555425	3.109501
161	1	0	4.346758	1.667141	3.126826
162	1	0	2.357207	-2.340119	5.329598
163	1	0	3.261072	1.886213	5.365789
164	1	0	2.264310	-0.119577	6.475878
165	6	0	6.808141	-1.966440	0.626002
166	1	0	6.483708	-2.775779	-0.020248
167	6	0	6.271133	-1.798374	1.911752
168	1	0	6.812502	-1.156362	2.605304
169	6	0	0.723814	-4.989022	-1.037840
170	6	0	1.634738	-5.932898	-0.539727
171	6	0	0.211468	-5.151781	-2.335652
172	6	0	1.968255	-7.061037	-1.294739
173	1	0	2.080828	-5.797803	0.437706

174	6	0	0.545214	-6.281557	-3.088529
175	1	0	-0.443599	-4.413337	-2.774430
176	6	0	1.417228	-7.239528	-2.565817
177	1	0	2.658365	-7.793550	-0.896615
178	1	0	0.133855	-6.409944	-4.081361
179	1	0	1.676527	-8.112390	-3.150799
180	6	0	4.680668	-1.336781	-1.960523
181	1	0	4.978299	-2.392556	-1.996402
182	1	0	3.651294	-1.266560	-2.343235
183	1	0	5.344146	-0.783757	-2.630451
184	6	0	8.001289	-1.230672	0.175867
185	8	0	8.288039	-1.551034	-1.144403
186	8	0	8.672180	-0.438245	0.872200
187	1	0	5.720888	-2.620260	2.364910
188	6	0	9.466768	-0.954191	-1.882702
189	6	0	9.354195	0.582084	-1.908457
190	6	0	10.768733	-1.439733	-1.217514
191	6	0	9.297407	-1.549373	-3.289792
192	1	0	8.354363	0.889636	-2.233392
193	1	0	9.533572	1.009021	-0.919303
194	1	0	10.097208	0.983619	-2.610738
195	1	0	10.801309	-2.535948	-1.188205
196	1	0	11.630759	-1.084946	-1.797347
197	1	0	10.853143	-1.053135	-0.198371
198	1	0	10.116118	-1.216952	-3.939851
199	1	0	9.303760	-2.644818	-3.249291
200	1	0	8.347974	-1.222280	-3.730186

TS10exoent

Center Number	Atomic Number	Atomic Type	Coordinates (Angstroms) X	Y	Z
1	7	0	4.426882	0.762987	-0.224946
2	6	0	5.588901	0.906374	-1.013283
3	8	0	6.102988	2.201913	-0.740466
4	6	0	5.528412	2.645756	0.502891
5	6	0	4.550899	1.622221	0.838070
6	6	0	5.749139	0.472290	-2.422524
7	6	0	5.297169	-0.787722	-2.877130
8	6	0	5.483408	-1.186380	-4.210305
9	6	0	6.152934	-0.338579	-5.113708
10	6	0	6.637499	0.904800	-4.662540
11	6	0	6.443129	1.308279	-3.330835
12	8	0	5.835513	3.716756	1.047292
13	79	0	3.103225	-0.776035	-0.362558
14	15	0	1.745705	-2.736600	-0.669288
15	6	0	2.596357	-3.716514	-1.968194
16	6	0	3.943391	-4.058953	-1.772540
17	6	0	4.641046	-4.777979	-2.746668
18	6	0	4.000715	-5.155792	-3.928624
19	6	0	2.663638	-4.810661	-4.138297
20	6	0	1.963577	-4.089170	-3.166593
21	6	0	0.036321	-2.402747	-1.304854
22	6	0	-0.011993	-1.472210	-2.461716
23	6	0	-1.111950	-1.340591	-3.212786
24	6	0	-2.322091	-2.116493	-2.904868
25	6	0	-2.371997	-2.916170	-1.758727
26	6	0	-1.171083	-3.027069	-0.881016
27	6	0	-3.599362	-3.732158	-1.542708
28	6	0	-4.614448	-3.680884	-2.422411
29	6	0	-4.547502	-2.882979	-3.572146
30	6	0	-3.468056	-2.128964	-3.838949
31	6	0	-1.240893	-3.968430	0.259035
32	6	0	-1.828840	-3.631578	1.443257
33	6	0	-1.875763	-4.655085	2.531115
34	6	0	-1.444561	-5.908573	2.336414
35	6	0	-0.955990	-6.313104	1.094607
36	6	0	-0.867258	-5.398700	0.034680
37	6	0	-0.435129	-5.916331	-1.289573
38	6	0	-0.067947	-7.201595	-1.430200
39	6	0	-0.103709	-8.096545	-0.351550
40	6	0	-0.527491	-7.712767	0.864711

41	15	0	-2.738851	-2.035429	1.709450
42	6	0	-2.604429	-1.566919	3.477319
43	6	0	-3.647201	-0.862297	4.098879
44	6	0	-3.572529	-0.542493	5.457620
45	6	0	-2.455087	-0.922518	6.205085
46	6	0	-1.403671	-1.605581	5.588500
47	6	0	-1.471067	-1.909166	4.225785
48	6	0	1.759846	-3.788152	0.832765
49	6	0	1.507537	-3.198602	2.078719
50	6	0	1.638033	-3.945002	3.254836
51	6	0	2.015459	-5.287533	3.194987
52	6	0	2.277419	-5.882460	1.960028
53	6	0	2.159968	-5.136486	0.783920
54	79	0	-2.716899	-0.050016	0.219467
55	15	0	-4.080272	1.818616	-0.621012
56	6	0	-4.459491	1.790596	-2.410827
57	6	0	-4.194351	2.896038	-3.235334
58	6	0	-4.505410	2.851573	-4.597155
59	6	0	-5.095064	1.709507	-5.143368
60	6	0	-5.366947	0.608309	-4.328747
61	6	0	-5.035980	0.642061	-2.970528
62	6	0	-3.621706	3.526282	-0.126589
63	6	0	-2.437104	3.802206	0.488245
64	6	0	-2.183587	5.187784	0.991709
65	6	0	-3.130537	6.179173	0.731493
66	6	0	-4.366758	5.814461	0.022122
67	6	0	-4.574320	4.542859	-0.350329
68	6	0	-0.976420	5.571616	1.771629
69	6	0	-0.810590	6.842918	2.179147
70	6	0	-1.759193	7.836213	1.891816
71	6	0	-2.880085	7.557729	1.203919
72	6	0	-1.389980	2.755961	0.669565
73	6	0	-0.626444	2.320454	-0.378460
74	6	0	0.354776	1.218942	-0.156791
75	6	0	0.591520	0.734872	1.067955
76	6	0	-0.123153	1.278501	2.230862
77	6	0	-1.137913	2.218504	2.039431
78	6	0	-1.885719	2.682528	3.238346
79	6	0	-1.515862	2.274377	4.464862
80	6	0	-0.445180	1.387766	4.650560
81	6	0	0.229912	0.874290	3.608049
82	15	0	-0.943201	2.941824	-2.107432
83	6	0	0.553487	2.408653	-3.041387
84	6	0	1.863928	2.659679	-2.597874
85	6	0	2.955492	2.299784	-3.389348
86	6	0	2.755382	1.662369	-4.615474
87	6	0	1.457584	1.402527	-5.060623
88	6	0	0.361540	1.787823	-4.283110
89	6	0	-0.511454	4.726884	-1.935745
90	6	0	-1.188717	5.649622	-2.745047
91	6	0	-0.913607	7.016340	-2.644577
92	6	0	0.046925	7.471872	-1.739029
93	6	0	0.730356	6.560885	-0.931131
94	6	0	0.448491	5.194324	-1.020667
95	6	0	-5.673151	1.597749	0.252128
96	6	0	-5.640681	1.390459	1.637191
97	6	0	-6.823825	1.168173	2.346572
98	6	0	-8.049749	1.166100	1.676733
99	6	0	-8.093052	1.397290	0.299085
100	6	0	-6.910092	1.617531	-0.413604
101	6	0	-4.491188	-2.558296	1.583488
102	6	0	-5.297620	-1.979673	0.593417
103	6	0	-6.646853	-2.325306	0.487492
104	6	0	-7.202238	-3.249598	1.375114
105	6	0	-6.408407	-3.824407	2.371483
106	6	0	-5.057789	-3.478147	2.479938
107	1	0	-4.694855	1.393211	2.163912
108	1	0	-6.789710	0.996450	3.414627
109	1	0	-8.966174	0.993052	2.225766
110	1	0	-9.044393	1.407665	-0.216852
111	1	0	-6.972771	1.812282	-1.473889
112	1	0	-5.251436	-0.217436	-2.349288
113	1	0	-5.830482	-0.273537	-4.751036
114	1	0	-5.343402	1.678768	-6.196291
115	1	0	-4.294489	3.705615	-5.227680
116	1	0	-3.747138	3.792834	-2.835937
117	1	0	2.056006	3.114364	-1.641316

118	1	0	3.955432	2.525254	-3.050648
119	1	0	3.603823	1.379121	-5.224092
120	1	0	1.299953	0.917253	-6.015025
121	1	0	-0.640235	1.612387	-4.654253
122	1	0	-5.506812	4.300524	-0.844809
123	1	0	-5.122986	6.567580	-0.177420
124	1	0	-3.600165	8.344197	0.999647
125	1	0	-1.584273	8.848074	2.234587
126	1	0	0.078481	7.108855	2.736684
127	1	0	-0.210371	4.840375	2.004926
128	1	0	-2.725015	3.362836	3.139216
129	1	0	-2.057041	2.635517	5.330112
130	1	0	-0.172133	1.094008	5.655943
131	1	0	1.039340	0.171251	3.780448
132	1	0	1.312917	-0.059262	1.200514
133	1	0	0.885228	0.776724	-0.987626
134	1	0	0.257822	8.530349	-1.659614
135	1	0	1.463652	6.915311	-0.218366
136	1	0	0.953009	4.519064	-0.345608
137	1	0	-1.930191	5.310672	-3.456621
138	1	0	-1.444366	7.722046	-3.270288
139	1	0	-4.880959	-1.267563	-0.104111
140	1	0	-7.260832	-1.875232	-0.281981
141	1	0	-8.247995	-3.516286	1.294512
142	1	0	-6.842221	-4.531626	3.066455
143	1	0	-4.472648	-3.907349	3.278067
144	1	0	-0.645987	-2.417487	3.758168
145	1	0	-0.529620	-1.885658	6.161911
146	1	0	-2.398644	-0.677947	7.257730
147	1	0	-4.384034	-0.006217	5.932066
148	1	0	-4.528132	-0.580431	3.540457
149	1	0	4.453898	-3.765415	-0.863678
150	1	0	5.679234	-5.038312	-2.586620
151	1	0	4.541597	-5.711091	-4.683838
152	1	0	2.169871	-5.098033	-5.057561
153	1	0	0.936858	-3.826766	-3.365844
154	1	0	-2.264909	-4.396328	3.507757
155	1	0	-1.499034	-6.618360	3.152981
156	1	0	-0.553893	-8.433672	1.676625
157	1	0	0.204449	-9.122664	-0.506833
158	1	0	0.254663	-7.557428	-2.400480
159	1	0	-0.427487	-5.269674	-2.156751
160	1	0	-3.691471	-4.379561	-0.677177
161	1	0	-5.500940	-4.276404	-2.244483
162	1	0	-5.381883	-2.879306	-4.261757
163	1	0	-3.435911	-1.533452	-4.746108
164	1	0	-1.095818	-0.694248	-4.082859
165	1	0	0.883295	-0.927576	-2.747189
166	1	0	2.109615	-5.865028	4.105329
167	1	0	2.570291	-6.923272	1.912676
168	1	0	2.363205	-5.626366	-0.156836
169	1	0	1.225479	-2.158349	2.142977
170	1	0	1.454797	-3.481319	4.215169
171	1	0	4.821045	-1.471486	-2.180315
172	1	0	6.815933	2.265381	-2.982694
173	1	0	5.129325	-2.160922	-4.539647
174	1	0	7.168857	1.560827	-5.347306
175	1	0	6.308086	-0.646845	-6.144383
176	6	0	6.887590	-0.228240	0.114936
177	1	0	7.811689	0.088463	-0.367291
178	6	0	6.683055	0.175052	1.445871
179	1	0	5.951048	-0.310921	2.083506
180	6	0	3.670239	1.712702	2.041296
181	1	0	2.828641	2.407449	1.891432
182	1	0	3.278846	0.729875	2.330751
183	1	0	4.263575	2.097810	2.877649
184	1	0	6.502266	-1.193318	-0.211214
185	6	0	7.614981	1.086710	2.121782
186	8	0	7.199794	1.318993	3.430026
187	8	0	8.647257	1.579353	1.615796
188	6	0	7.988583	2.205386	4.369018
189	6	0	9.362093	1.557120	4.633137
190	6	0	8.107791	3.624365	3.780862
191	6	0	7.115445	2.194026	5.634463
192	1	0	9.238971	0.536134	5.016140
193	1	0	9.959782	1.523998	3.718624
194	1	0	9.906258	2.143374	5.384931

195	1	0	7.126910	3.994031	3.461902
196	1	0	8.509119	4.299399	4.548828
197	1	0	8.770295	3.638526	2.913072
198	1	0	7.618126	2.739735	6.442580
199	1	0	6.148649	2.673222	5.439754
200	1	0	6.935247	1.164726	5.966362

TS11

Center Number	Atomic Number	Atomic Type	Coordinates (Angstroms) X	Y	Z
1	6	0	5.431289	-3.561001	-3.952496
2	6	0	5.958871	-2.987384	-2.791299
3	6	0	5.119931	-2.415382	-1.831240
4	6	0	3.731456	-2.431495	-1.998314
5	6	0	3.184687	-3.000105	-3.250946
6	6	0	4.053820	-3.563005	-4.184379
7	15	0	2.647653	-1.605785	-0.772802
8	6	0	2.926666	-2.344329	0.880786
9	6	0	2.238844	-1.841532	1.925204
10	6	0	2.464417	-2.299953	3.229439
11	6	0	3.415243	-3.294827	3.464315
12	6	0	4.129266	-3.826694	2.390277
13	6	0	3.885087	-3.354133	1.097100
14	79	0	3.096936	0.723540	-0.655571
15	8	0	5.771000	0.561823	-0.593833
16	6	0	6.436201	1.550290	-0.107854
17	8	0	7.270369	1.391284	0.999103
18	6	0	7.756205	0.046863	1.470625
19	6	0	6.588617	-0.772095	2.062145
20	7	0	3.411958	2.756127	-0.475275
21	6	0	3.613948	3.467663	0.634030
22	8	0	3.832696	4.807931	0.343435
23	6	0	3.827228	4.942359	-1.118974
24	6	0	3.773633	3.559206	-1.604926
25	6	0	3.484095	3.097176	2.042004
26	6	0	3.862889	1.817164	2.512084
27	6	0	3.746621	1.504673	3.875133
28	6	0	3.241144	2.456587	4.782884
29	6	0	2.871805	3.735954	4.320264
30	6	0	3.003030	4.062062	2.961574
31	8	0	3.942789	6.038978	-1.666212
32	6	0	3.165700	3.262917	-2.954340
33	6	0	0.915538	-1.988461	-1.270518
34	6	0	0.306142	-1.068259	-2.257117
35	6	0	-0.653482	-1.483612	-3.099483
36	6	0	-1.149838	-2.876430	-3.076375
37	6	0	-0.702559	-3.777336	-2.104738
38	6	0	0.280823	-3.328756	-1.090886
39	6	0	-1.227106	-5.167519	-2.143020
40	6	0	-2.111631	-5.530073	-3.087892
41	6	0	-2.561177	-4.622198	-4.057402
42	6	0	-2.123392	-3.351621	-4.085723
43	6	0	0.496868	-4.140044	0.122092
44	6	0	-0.304829	-3.978057	1.269928
45	6	0	-0.006290	-4.831538	2.453759
46	6	0	0.980383	-5.739615	2.435475
47	6	0	1.745232	-5.961442	1.287821
48	6	0	1.510318	-5.227090	0.115924
49	6	0	2.288806	-5.586383	-1.096029
50	6	0	3.241838	-6.531777	-1.035614
51	6	0	3.511851	-7.224754	0.152873
52	6	0	2.814199	-6.988171	1.277186
53	15	0	-1.858164	-2.973187	1.254779
54	6	0	-3.047842	-4.125041	0.488357
55	6	0	-3.842704	-3.694003	-0.576103
56	6	0	-4.776674	-4.558020	-1.153376
57	6	0	-4.924380	-5.868495	-0.684942
58	6	0	-4.142020	-6.342919	0.372687
59	6	0	-3.203042	-5.509354	0.979244
60	6	0	-2.437906	-2.837855	2.989198
61	6	0	-3.766708	-3.131189	3.341031

62	6	0	-4.191001	-2.996694	4.666174
63	6	0	-3.295959	-2.565084	5.647838
64	6	0	-1.979244	-2.253736	5.302094
65	6	0	-1.558217	-2.367864	3.973867
66	79	0	-2.289948	-0.785896	0.119249
67	15	0	-4.246524	0.521206	-0.702864
68	6	0	-5.659077	-0.608090	-0.441336
69	6	0	-5.845374	-1.202290	0.896687
70	6	0	-6.868516	-2.126577	1.098569
71	6	0	-7.716436	-2.474727	0.043951
72	6	0	-7.555688	-1.901727	-1.222926
73	6	0	-6.536827	-0.975759	-1.468584
74	6	0	-4.227458	0.928662	-2.485472
75	6	0	-4.546125	2.214010	-2.952982
76	6	0	-4.466142	2.506372	-4.317634
77	6	0	-4.079164	1.516955	-5.224308
78	6	0	-3.778290	0.231799	-4.767607
79	6	0	-3.852446	-0.061650	-3.402485
80	6	0	-4.722645	2.032539	0.230190
81	6	0	-3.810418	2.721640	0.966376
82	6	0	-4.230657	3.950654	1.698286
83	6	0	-5.561123	4.359655	1.609391
84	6	0	-6.498021	3.562183	0.799083
85	6	0	-6.064188	2.465475	0.156891
86	6	0	-5.981366	5.593058	2.308744
87	6	0	-5.081206	6.303942	3.011314
88	6	0	-3.739033	5.902115	3.102415
89	6	0	-3.294823	4.787701	2.493697
90	6	0	-2.385132	2.319037	0.989053
91	6	0	-1.485323	2.691088	-0.134526
92	6	0	-0.081495	2.210155	-0.091662
93	6	0	0.401995	1.598102	1.001884
94	6	0	-0.432030	1.384102	2.204712
95	6	0	-1.793415	1.700497	2.196130
96	6	0	-2.575083	1.475014	3.439470
97	6	0	-1.960299	1.039949	4.552334
98	6	0	-0.587922	0.752501	4.565477
99	6	0	0.165636	0.882303	3.461292
100	15	0	-2.090837	3.950673	-1.358874
101	6	0	-1.269643	5.404168	-0.581014
102	6	0	-2.078264	6.454965	-0.127576
103	6	0	-1.509853	7.557691	0.515939
104	6	0	-0.127067	7.621853	0.703068
105	6	0	0.688615	6.585727	0.241870
106	6	0	0.122953	5.479934	-0.400239
107	6	0	-0.997070	3.655349	-2.814192
108	6	0	-0.504761	4.697078	-3.521030
109	6	0	0.207207	4.490324	-4.710207
110	6	0	0.393315	3.194287	-5.196751
111	6	0	-0.138465	2.114930	-4.490533
112	6	0	-0.845404	2.346859	-3.305999
113	6	0	6.404713	2.892690	-0.595653
114	6	0	5.732626	3.197085	-1.808781
115	6	0	8.451822	-0.686189	0.306465
116	6	0	8.763362	0.429786	2.567186
117	1	0	-5.184361	-0.936573	1.711908
118	1	0	-7.004792	-2.576423	2.073476
119	1	0	-8.507759	-3.194302	0.209282
120	1	0	-8.225867	-2.183268	-2.024815
121	1	0	-6.443594	-0.556214	-2.459634
122	1	0	-3.617388	-1.059032	-3.056397
123	1	0	-3.484479	-0.534215	-5.472248
124	1	0	-4.015332	1.745169	-6.280264
125	1	0	-4.700423	3.501929	-4.671573
126	1	0	-4.842168	2.998398	-2.272525
127	1	0	-0.674903	5.714367	-3.194530
128	1	0	0.601242	5.335604	-5.259293
129	1	0	0.939158	3.029309	-6.116486
130	1	0	-0.013169	1.109025	-4.866609
131	1	0	-1.289818	1.513195	-2.779806
132	1	0	-6.774022	1.910913	-0.443335
133	1	0	-7.537019	3.865275	0.712158
134	1	0	-7.012487	5.928366	2.252267
135	1	0	-5.396625	7.207953	3.516583
136	1	0	-3.045147	6.506672	3.672640
137	1	0	-2.246889	4.519506	2.579153
138	1	0	-3.640899	1.677021	3.467355

139	1	0	-2.537891	0.897196	5.456817
140	1	0	-0.130811	0.396731	5.480002
141	1	0	1.216291	0.628160	3.499590
142	1	0	1.434384	1.277333	1.018048
143	1	0	0.579240	2.369299	-0.934969
144	1	0	0.312602	8.475475	1.202385
145	1	0	1.759581	6.641866	0.381827
146	1	0	0.774062	4.699840	-0.763815
147	1	0	-3.152693	6.410594	-0.255916
148	1	0	-2.142307	8.359676	0.873990
149	1	0	-3.735301	-2.691555	-0.966282
150	1	0	-5.386886	-4.211030	-1.977222
151	1	0	-5.650883	-6.523048	-1.148568
152	1	0	-4.267805	-7.358772	0.723918
153	1	0	-2.607862	-5.874340	1.805118
154	1	0	-0.541135	-2.108293	3.714059
155	1	0	-1.287874	-1.911706	6.061064
156	1	0	-3.624352	-2.465080	6.674193
157	1	0	-5.215008	-3.225831	4.931419
158	1	0	-4.482421	-3.453190	2.598283
159	1	0	-0.605228	-4.756137	3.351731
160	1	0	1.163208	-6.332060	3.323912
161	1	0	3.040520	-7.551709	2.177661
162	1	0	4.289790	-7.977591	0.159471
163	1	0	3.806686	-6.772678	-1.927319
164	1	0	2.081075	-5.106909	-2.042968
165	1	0	-0.907316	-5.899490	-1.408602
166	1	0	-2.489940	-6.544360	-3.099017
167	1	0	-3.274765	-4.954410	-4.800595
168	1	0	-2.482577	-2.677750	-4.855858
169	1	0	-1.045497	-0.795835	-3.839374
170	1	0	0.688957	-0.056430	-2.344367
171	1	0	3.592632	-3.654125	4.469455
172	1	0	4.860609	-4.607025	2.555636
173	1	0	4.428615	-3.798809	0.279225
174	1	0	1.499238	-1.070982	1.762850
175	1	0	1.907302	-1.879816	4.056320
176	1	0	4.296203	1.095843	1.827216
177	1	0	2.746896	5.054810	2.604196
178	1	0	4.062411	0.527432	4.232619
179	1	0	2.497875	4.479698	5.018942
180	1	0	3.155037	2.213043	5.838790
181	1	0	6.001322	4.126118	-2.312919
182	1	0	6.956185	3.646770	-0.045834
183	1	0	5.555427	-1.979647	-0.944717
184	1	0	2.124012	-2.992867	-3.455620
185	1	0	7.029964	-2.983274	-2.635139
186	1	0	3.660320	-3.996252	-5.094784
187	1	0	6.098025	-3.998748	-4.684123
188	1	0	3.443563	2.263292	-3.310242
189	1	0	2.072579	3.319999	-2.906607
190	1	0	3.513742	3.999275	-3.686010
191	1	0	5.560984	2.354240	-2.479643
192	1	0	7.746716	-0.911548	-0.497633
193	1	0	9.259792	-0.066203	-0.099102
194	1	0	8.890015	-1.624931	0.669166
195	1	0	8.272936	1.023001	3.347131
196	1	0	9.190006	-0.472137	3.024274
197	1	0	9.577543	1.028496	2.145147
198	1	0	6.968394	-1.725570	2.452395
199	1	0	6.124809	-0.224333	2.891028
200	1	0	5.837126	-0.973733	1.294492

TS11ent

Center Number	Atomic Number	Atomic Type	Coordinates (Angstroms) X	Y	Z
1	7	0	-3.734740	2.667918	-0.671593
2	6	0	-4.846917	3.169649	-0.125331
3	8	0	-5.336322	4.244281	-0.863195
4	6	0	-4.450857	4.429999	-2.007085
5	6	0	-3.557128	3.264154	-1.970592

6	6	0	-5.525042	2.900607	1.144320
7	6	0	-4.985287	2.055105	2.143937
8	6	0	-5.659741	1.863192	3.357989
9	6	0	-6.890870	2.504984	3.596803
10	6	0	-7.433522	3.352663	2.610885
11	6	0	-6.758370	3.554867	1.398010
12	8	0	-4.613321	5.350134	-2.809398
13	79	0	-2.938983	0.814280	-0.165265
14	15	0	-2.514975	-1.393478	0.645818
15	6	0	-3.781837	-1.627539	1.955418
16	6	0	-5.121333	-1.347491	1.645227
17	6	0	-6.119710	-1.489719	2.611941
18	6	0	-5.786323	-1.900681	3.903956
19	6	0	-4.453858	-2.165438	4.229305
20	6	0	-3.453001	-2.025077	3.262838
21	6	0	-0.878652	-1.658938	1.475581
22	6	0	-0.526244	-0.620445	2.477213
23	6	0	0.472956	-0.796076	3.350521
24	6	0	1.263431	-2.036118	3.337939
25	6	0	1.053183	-2.993935	2.341032
26	6	0	-0.020675	-2.783044	1.328379
27	6	0	1.848384	-4.251095	2.415582
28	6	0	2.738822	-4.434918	3.405903
29	6	0	2.930970	-3.472455	4.406183
30	6	0	2.237084	-2.322190	4.412853
31	6	0	-0.258039	-3.859861	0.342445
32	6	0	0.501194	-3.986711	-0.782933
33	6	0	0.226936	-5.132394	-1.702619
34	6	0	-0.695475	-6.060576	-1.413812
35	6	0	-1.415810	-6.008278	-0.220734
36	6	0	-1.206965	-4.961614	0.689199
37	6	0	-1.929631	-5.018770	1.986116
38	6	0	-2.809765	-6.004672	2.230824
39	6	0	-3.055301	-7.017698	1.293140
40	6	0	-2.405360	-7.058038	0.117463
41	15	0	1.975564	-2.917350	-1.133056
42	6	0	2.208074	-2.801221	-2.948231
43	6	0	3.499358	-2.668395	-3.482647
44	6	0	3.689426	-2.610237	-4.866239
45	6	0	2.591245	-2.682776	-5.726594
46	6	0	1.301154	-2.793878	-5.201943
47	6	0	1.110040	-2.832994	-3.817862
48	6	0	-2.891385	-2.652722	-0.632801
49	6	0	-2.284949	-2.548458	-1.892048
50	6	0	-2.650564	-3.414413	-2.928102
51	6	0	-3.617517	-4.397507	-2.711320
52	6	0	-4.227860	-4.510309	-1.461283
53	6	0	-3.873496	-3.639584	-0.427203
54	79	0	2.563973	-0.789554	-0.014609
55	15	0	4.438985	0.612711	0.758982
56	6	0	4.611541	0.787901	2.573215
57	6	0	4.672464	2.049881	3.186849
58	6	0	4.820255	2.154258	4.572722
59	6	0	4.921612	1.001546	5.354599
60	6	0	4.866231	-0.256642	4.751308
61	6	0	4.693504	-0.364076	3.367941
62	6	0	4.697451	2.254134	-0.024984
63	6	0	3.765418	2.812226	-0.847452
64	6	0	4.086534	4.091107	-1.551912
65	6	0	5.298962	4.723161	-1.273789
66	6	0	6.240208	4.079666	-0.343733
67	6	0	5.929398	2.899712	0.213460
68	6	0	3.185362	4.728883	-2.548236
69	6	0	3.533647	5.892129	-3.127391
70	6	0	4.744672	6.531736	-2.821211
71	6	0	5.614384	6.003643	-1.942487
72	6	0	2.432555	2.179747	-1.056112
73	6	0	1.457827	2.240203	-0.099390
74	6	0	0.171279	1.521288	-0.325731
75	6	0	-0.098964	0.931853	-1.498125
76	6	0	0.885635	0.963652	-2.588055
77	6	0	2.148102	1.514492	-2.361161
78	6	0	3.138828	1.454354	-3.468660
79	6	0	2.781914	0.962637	-4.668128
80	6	0	1.487984	0.476371	-4.905537
81	6	0	0.560887	0.443529	-3.932907
82	15	0	1.801761	3.045026	1.546257

83	6	0	0.143719	3.198948	2.337456
84	6	0	-0.971421	3.765587	1.693513
85	6	0	-2.174354	3.932142	2.384437
86	6	0	-2.282747	3.521011	3.714620
87	6	0	-1.183281	2.951441	4.359284
88	6	0	0.027550	2.803352	3.677194
89	6	0	2.016923	4.803186	1.037399
90	6	0	2.922354	5.592300	1.760079
91	6	0	3.141688	6.925786	1.403025
92	6	0	2.453113	7.483304	0.323457
93	6	0	1.549216	6.705588	-0.403338
94	6	0	1.337792	5.367632	-0.056870
95	6	0	5.914912	-0.304320	0.183959
96	6	0	5.927426	-0.768708	-1.137667
97	6	0	7.007311	-1.516990	-1.613528
98	6	0	8.088907	-1.794161	-0.773880
99	6	0	8.095323	-1.313324	0.538386
100	6	0	7.014847	-0.565962	1.017716
101	6	0	3.371054	-3.993498	-0.633604
102	6	0	4.254505	-3.537755	0.354676
103	6	0	5.348627	-4.316155	0.739490
104	6	0	5.569706	-5.556110	0.135650
105	6	0	4.698589	-6.014681	-0.856576
106	6	0	3.604090	-5.235393	-1.244658
107	1	0	5.093543	-0.557337	-1.795051
108	1	0	7.004462	-1.882979	-2.632087
109	1	0	8.924821	-2.374731	-1.141867
110	1	0	8.939156	-1.518396	1.184325
111	1	0	7.052618	-0.189952	2.029414
112	1	0	4.650007	-1.344950	2.912984
113	1	0	4.952046	-1.149607	5.356607
114	1	0	5.045069	1.083147	6.426684
115	1	0	4.862877	3.129792	5.039570
116	1	0	4.605469	2.955855	2.604470
117	1	0	-0.930453	4.065347	0.660936
118	1	0	-3.024085	4.379893	1.886960
119	1	0	-3.217418	3.646027	4.245729
120	1	0	-1.264445	2.637458	5.391772
121	1	0	0.882092	2.390863	4.198468
122	1	0	6.653620	2.439641	0.874104
123	1	0	7.195841	4.546709	-0.125664
124	1	0	6.546672	6.516842	-1.727405
125	1	0	4.983641	7.469582	-3.306276
126	1	0	2.861803	6.350146	-3.842140
127	1	0	2.234041	4.276196	-2.805855
128	1	0	4.152067	1.815083	-3.326084
129	1	0	3.508686	0.940206	-5.470181
130	1	0	1.238109	0.101079	-5.889596
131	1	0	-0.426891	0.042057	-4.139053
132	1	0	-1.049034	0.436878	-1.649457
133	1	0	-0.564298	1.452422	0.461677
134	1	0	2.625511	8.514873	0.045417
135	1	0	1.029736	7.132224	-1.251559
136	1	0	0.679098	4.773868	-0.672660
137	1	0	3.460577	5.174075	2.600812
138	1	0	3.845621	7.526831	1.963784
139	1	0	4.091553	-2.584462	0.835840
140	1	0	6.023921	-3.957288	1.505532
141	1	0	6.417841	-6.159096	0.432763
142	1	0	4.876444	-6.970793	-1.331489
143	1	0	2.962089	-5.593753	-2.034243
144	1	0	0.109223	-2.896050	-3.427561
145	1	0	0.447866	-2.832390	-5.866506
146	1	0	2.738527	-2.640905	-6.797869
147	1	0	4.688587	-2.514834	-5.270994
148	1	0	4.361382	-2.629371	-2.832212
149	1	0	-5.391606	-1.015831	0.653792
150	1	0	-7.149837	-1.271743	2.361372
151	1	0	-6.558633	-2.006257	4.654670
152	1	0	-4.194988	-2.475374	5.233456
153	1	0	-2.433098	-2.224422	3.550461
154	1	0	0.777453	-5.232518	-2.629677
155	1	0	-0.864225	-6.871924	-2.111498
156	1	0	-2.610398	-7.862858	-0.582408
157	1	0	-3.775072	-7.791323	1.528498
158	1	0	-3.335350	-6.023028	3.177157
159	1	0	-1.743727	-4.277844	2.751800

160	1	0	1.722295	-5.033223	1.674412
161	1	0	3.313927	-5.351632	3.438501
162	1	0	3.647180	-3.665232	5.194705
163	1	0	2.386954	-1.605282	5.213862
164	1	0	0.673256	-0.038399	4.100076
165	1	0	-1.128297	0.280436	2.545406
166	1	0	-3.895228	-5.070087	-3.512281
167	1	0	-4.975919	-5.273869	-1.292078
168	1	0	-4.360198	-3.755811	0.529635
169	1	0	-1.544958	-1.784102	-2.077103
170	1	0	-2.191434	-3.319735	-3.903058
171	1	0	-4.026411	1.570044	1.985038
172	1	0	-7.169981	4.216678	0.643539
173	1	0	-5.224488	1.221561	4.120450
174	1	0	-8.378650	3.858423	2.789257
175	1	0	-7.414452	2.353087	4.537004
176	6	0	-5.710805	1.439090	-2.679816
177	1	0	-6.687451	1.892800	-2.770747
178	6	0	-4.573530	2.100138	-3.226420
179	1	0	-4.757899	2.866239	-3.981711
180	6	0	-2.176251	3.392570	-2.578751
181	1	0	-1.439188	3.726330	-1.837552
182	1	0	-1.835427	2.448548	-3.019427
183	1	0	-2.212048	4.139737	-3.379132
184	1	0	-3.720163	1.456119	-3.441323
185	6	0	-5.551034	0.149359	-2.085518
186	8	0	-4.398402	-0.395752	-1.894934
187	8	0	-6.609042	-0.643620	-1.644634
188	6	0	-8.087261	-0.383338	-1.733384
189	6	0	-8.472910	0.841118	-0.879101
190	6	0	-8.528149	-0.268519	-3.206125
191	6	0	-8.656844	-1.668103	-1.103335
192	1	0	-8.061432	0.737949	0.131841
193	1	0	-8.113624	1.783715	-1.300651
194	1	0	-9.565829	0.901426	-0.801063
195	1	0	-8.218974	-1.161875	-3.761157
196	1	0	-9.621729	-0.192739	-3.257686
197	1	0	-8.104100	0.607125	-3.704849
198	1	0	-9.753757	-1.646759	-1.113291
199	1	0	-8.314803	-2.546003	-1.662094
200	1	0	-8.315206	-1.766556	-0.066637

7. References

[1] Fisk, J. S.; Mosey, R. A.; Tepe, J. J. *Chem. Soc. Rev.* **2007**, *36*, 1432-1440.
[2] Hewlett, N. M.; Hupp, C. D.; Tepe, J. J. *Synthesis* **2009**, 2825-2839.
[3] Alba, A-N. R.; Ríos, R. *Chem. Asian J.* **2011**, *6*, 720-734.
[4] El-Mekabaty, A. *Int. J. Modern Org. Chem.* **2013**, *2*, 40-66.
[5] Melhado, A. D.; Luparia, M.; Toste, F. D. *J. Am. Chem. Soc.* **2007**, *129*, 12638-12639.
[6] Melhado, A. D.; Amarante, G. W.; Wang, J.; Luparia, M.; Toste, F. D. *J. Am. Chem. Soc.* **2011**, *133*, 3517-3527.
[7] Svensson, M.; Humbel, S.; Morokuma, K. *J. Chem. Phys.* **1996**, *105*, 3654-3661.
[8] Vreven, T.; Morokuma, K. *J. Comput. Chem.* **2000**, *21*, 1419-1432.
[9] Dapprich, S.; Komaromi, I.; Byun, K. S.; Morokuma, K.; Frisch, M. J. *J. Mol. Struc. (Theochem)* **1999**, 461-462.
[10] Gaussian09, Revision A.02, Gaussian Inc.: Wallingford, CT, 2009.
[11] Becke, A. D. *J. Chem. Phys.* **1983**, *98*, 5648-5652.
[12] Becke, A. D. *Phys. Rev. A* **1998**, *38*, 3098-3100.
[13] Kohn, W.; Becke, A. D.; Parr, R. G. *J. Phys. Chem.* **1996**, *100*, 12974-12980.
[14] Lee, C. ; Yang, W.; Parr, R. G. *Phys. Rev. B.* **1998**, *37*, 785-789.
[15] Vosko, S. H.; Wilk, L.; Nusair, M. *Can. J. Phys.* **1980**, *58*, 1200-1211.
[16] Stephens, P. J.; Devlin, F. J.; Chavalowski, C. F.; Frisch, M. J. *J. Phys. Chem.* **1994**, *98*, 11623-11627.
[17] May, P. J.; Wadt, W. R. *J. Chem. Phys.* **1985**, *82*, 299-303.
[18] Rappé, A. K.; Casewit, C. J.; Colwell, K. S.; Goddard III, W. A.; Skid, W. M. *J. Am. Chem. Soc.* **1992**, *114*¸ 10024-10035.
[19] Zhao, Y.; Truhlar, D. G. *Theoretical Chem. Acc*. **2008**, *120*, 215-241.
[20] Zhao, Y.; Truhlar, D. G., *J. Chem. Theory Comput*. **2009**, *4*, 1849-1868.
[21] Zhao, Y.; Truhlar, D. G., *Acc. Chem. Res*. **2008**, *41*, 157-167
[22] Glendening, E. D.; Weinhold, F. *J. Comp. Chem*. **1998**, *19*, 593-609.
[23] Glendening, E. D.; Weinhold, F. *J. Comp. Chem.* **1998**, *19*, 610-627.
[24] Glendening, E. D.; Badenhoop,J. K.; Weinhold, F. *J. Comp. Chem*. **1998**, *19*, 628-646.
[25] Martín-Rodríguez, M.; Nájera, C.; Sansano, J. M. *Synlett* **2012**, 62-65.
[26] Wheaton, C. A.; Jennings, M. C.; Puddephatt, R. J. *J. Am. Chem. Soc.* **2006**, *128*, 15370-15371.
[27] Wheaton, C. A.; Puddephatt, R. J. *Angew. Chem. Int. Ed.* **2007**, *46*, 4461-4463.
[28] Wheaton, C. A.; Jennings, M. C.; Puddephatt, R. J. *Z. Naturforsch* **2009**, *64b*, 1569-1577.
[29] Oderaotoshi, Y.; Cheng, W.; Fujitomi, S.; Kasano, Y.; Minakata, S.; Komatsu M. *Org. Lett.* **2003**, *5*, 5043–5046.
[30] Fukuzawa, S.; Oki, H. *Org. Lett.* **2008**, *10*, 1747–1750.
